# *In Situ* Forming, Enzyme-Responsive Peptoid-Peptide Hydrogels: An Advanced Long-Acting Injectable Drug Delivery System

**DOI:** 10.1021/jacs.4c03751

**Published:** 2024-06-26

**Authors:** Sophie M. Coulter, Sreekanth Pentlavalli, Yuming An, Lalitkumar K. Vora, Emily R. Cross, Jessica V. Moore, Han Sun, Ralf Schweins, Helen O. McCarthy, Garry Laverty

**Affiliations:** † Biofunctional Nanomaterials Group, School of Pharmacy, Queen’s University Belfast, Medical Biology Centre, 97 Lisburn Road, Belfast, Co. Antrim BT9 7BL, N. Ireland; ‡ Large Scale Structures Group, Institut Laue − Langevin, 71 Avenue des Martyrs, CS 20156, Grenoble Cedex 9, 38042, France

## Abstract

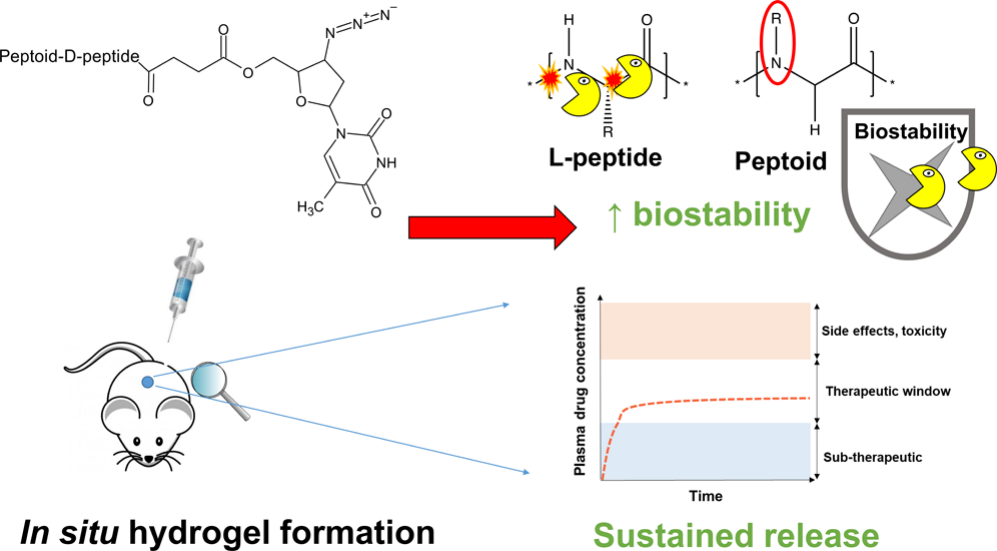

Long-acting drug delivery systems are promising platforms to improve patient adherence to medication by delivering drugs over sustained periods and removing the need for patients to comply with oral regimens. This research paper provides a proof-of-concept for the development of a new optimized *in situ* forming injectable depot based on a tetrabenzylamine-tetraglycine-d-lysine-O-phospho-d-tyrosine peptoid-D-peptide formulation ((*N*Phe)_4_GGGGk(AZT)y(p)-OH). The chemical versatility of the peptoid-peptide motif allows low-molecular-weight drugs to be precisely and covalently conjugated. After subcutaneous injection, a hydrogel depot forms from the solubilized peptoid-peptide-drug formulation in response to phosphatase enzymes present within the skin space. This system is able to deliver clinically relevant concentrations of a model drug, the antiretroviral zidovudine (AZT), for 35 days in Sprague–Dawley rats. Oscillatory rheology demonstrated that hydrogel formation began within ∼30 s, an important characteristic of *in situ* systems for reducing initial drug bursts. Gel formation continued for up to ∼90 min. Small-angle neutron scattering data reveal narrow-radius fibers (∼0.78–1.8 nm) that closely fit formation via a flexible cylinder elliptical model. The inclusion of non-native peptoid monomers and D-variant amino acids confers protease resistance, enabling enhanced biostability to be demonstrated *in vitro*. Drug release proceeds via hydrolysis of an ester linkage under physiological conditions, releasing the drug in an unmodified form and further reducing the initial drug burst. Subcutaneous administration of (*N*Phe)_4_GGGGk(AZT)y(p)-OH to Sprague–Dawley rats resulted in zidovudine blood plasma concentrations within the 90% maximal inhibitory concentration (IC_90_) range (30–130 ng mL^–1^) for 35 days.

## 1 Introduction

Peptide-based hydrogel systems have gained significant attention in recent years within several healthcare applications, including as drug delivery platforms, topical antimicrobial agents, tissue engineering/cell scaffolds and wound healing.^1^ The popularity of these materials is due to the unique chemical and functional versatility of peptides, which can be tailored to undergo self-assembly to form supramolecular hydrogels in response to physiological stimuli including pH, salt strength and the presence of specific enzymes. Peptides are highly amenable to modification at the molecular scale, enabling fine-tuning of desired functional properties, for example hydrogel formation in response to physiological stimuli, improved mechanical strength, sustained drug release and antimicrobial properties.^2,3^ Peptide hydrogels can also be designed with improved biocompatibility and biodegradability compared to synthetic polymeric systems, making them attractive candidates for novel biomedical technologies. However, there are limitations associated with the peptide hydrogel approach. For example, naturally occurring L-α enantiomeric forms of peptide amino acid building blocks are more easily recognized by proteolytic enzymes and are prone to rapid degradation *in vivo*. This results in rapid breakdown and clearance from the body, reducing their potential use as long-acting drug delivery platforms.^4^ Attempts to enhance the biostability of native peptides to improve their bioavailability and enhance their pharmacokinetic profile have resulted in the study of non-natural peptide-like molecules, termed peptide-mimetics.^5^ These efforts focus on manipulation of the chemical structure of amino acids to create non-native peptide analogues. These include D-amino acids,^6,7^ β-amino acids,^8^ γ-amino acids,^9^ and peptoids.^10^ The clinical promise of peptide-mimetic hydrogels as long-acting drug delivery platforms is highlighted by the success of degarelix (Firmagon), a hormonal therapy utilized for the treatment of advanced castration-sensitive prostate cancer.^11^ It is a synthetic peptide-mimetic composed of 10 amino acid units, 5 of which are D-amino acids. The degarelix molecule provides both the hydrogel forming ability and active ingredient. Firmagon is injected as a solution that forms a gel depot upon subcutaneous administration in response to physiological temperature and the presence of salts (ionic strength). This approach enables the sustained release of degarelix to clinically relevant concentrations for 28 days.

D-peptides offer the most popular approach for developing peptide-mimetic designs with improved biostability. However, similar to β-homo and γ-amino acid building blocks, they are relatively expensive to synthesize, limiting their wider large-scale manufacture.^12^ Peptoids, first reported by Zuckermann and colleagues in 1992, are oligomers of *N*-substituted glycines in which the side chain, commonly referred to as the R-group, is appended to the amide nitrogen rather than the α-carbon as in natural L-α peptides.^13^ This modification renders the backbone achiral and conformationally flexible, allowing the properties to be tuned exclusively via side-chain variation.^14^ Their synthesis, primarily by a submonomer method and bromo-acetylation, offers reduced cost and ease of synthesis relative to other peptide-mimetics, and their pharmaceutical use warrants further study.^15,16^ Improved resistance to proteolysis is also provided by the peptoid backbone, enabling the potential use of these materials as biostable long-acting drug delivery platforms.^17^ Peptoids are readily synthesized from a wide variety of commercially available primary amines, and the synthetic protocols for their manufacture are well established.^18^ They have been reported throughout the literature as the building blocks of various nanostructures,^19^ including nanosheets,^20,21^ nanoribbons,^22,23^ nanotubes,^24,25^ and nanofibers.^26,27^ However, their use as hydrogels has been relatively unexplored, particularly within the field of drug delivery. The lack of rotation around the peptoid motif and reduced hydrogen bond capacity mean that it is difficult to create true hydrogels from peptoid-only molecules in water. However, their ability to self-assemble in water to form stable nanosheets via hydrophobic and electrostatic interactions between peptoid side chain groups has been previously established.^28−30^ Wu and colleagues investigated the formation of hydrogels from peptoids alone but were unsuccessful, owing to the lack of hydrogen bonding.^31^ These researchers successfully formed hydrogels based on peptoid-peptide hybrids. They achieved this by substituting a tetra-phenylalanine (Phe-Phe-Phe-Phe) peptide sequence, a well-established peptide gelating motif, for a corresponding peptoid variant (*N*Phe)_4_ and adding several tripeptide sequences (RGD, YSV, VPP, GGG). This was sufficient to bestow hydrogel-forming ability to peptoid-peptide hybrids at pH 7.4 in phosphate buffered saline (PBS) at a concentration of 10 mg mL^–1^.

We initially focused on the synthesis of a predominantly peptoid sequence (*N*Phe)_4_(*N*Lys)Y(p)-OH combined with a covalently attached phosphate group (p) to increase solubility in water and act as a potential phosphatase enzyme trigger for gelation upon removal.^32^ However, this approach was unable to achieve gelation in response to phosphatase enzyme, up to our maximum tested concentration of 5% w/v. Therefore, to optimize the ability of the peptoid molecule to form hydrogels, we introduced peptide monomers sequentially to create a monoglycine containing (*N*Phe)_4_(*N*Lys)Y(p)G-OH molecule and triglycine (*N*Phe)_4_(*N*Lys)Y(p)GGG-OH, neither of which were able to gelate (Table S1). A final hydrogelating peptoid-peptide template was initially discovered, comprising a peptoid-L-peptide, tetrabenzylamine-tetraglycine-l-lysine-O-phospho-l-tyrosine ((*N*Phe)_4_GGGGKY(p)-OH) and its peptoid-D-peptide variant (*N*Phe)_4_GGGGky(p)-OH which was hypothesized to provide superior biostability.

The HIV/AIDS antiretroviral zidovudine was selected as a model low-molecular-weight drug due to the wide availability of data relating to its clinical use and in drug delivery research. Zidovudine is also sparingly soluble in water making its pharmaceutical formulation challenging within aqueous systems.^33^ HIV/AIDS also remains a significant global health concern and there is significant scope for the use of long-acting injectable platforms to improve treatment and prevention strategies. The most recent (2021) statistics from the World Health Organization indicate that there were ∼38.4 million people worldwide living with HIV/AIDS, with ∼1.5 million new HIV infections that year.^34^ UNAIDS outlined its Fast-Track strategy in 2014, with the goal of ending the AIDS epidemic by 2030. They aim to achieve this goal by eliminating new infections and improving the standard of care for current HIV/AIDS patients.^35^ As this deadline approaches, considerable progress has been made to meet this target. Global access to antiretroviral therapy has improved dramatically in the past decade. An estimated 75% of people living with HIV/AIDS received therapy in 2021, compared to only 25% in 2010.^36^ Currently licensed oral antiretroviral therapy options are highly efficacious at maintaining undetectable viral loads for several years. However, nonadherence to oral drug dosage regimens, primarily due to pill fatigue, remains a key barrier to achieving effective control of infection.^37^ Adequate adherence to therapeutic regimens is imperative for reducing the risk of viral rebound, disease progression, antimicrobial resistance and ultimately treatment failure.^38,39^ There is a clear need for novel antiretroviral therapy delivery options to overcome these issues. Current efforts are focused on simplifying existing therapies, including reducing the number of drugs required and increasing the length of the dosing interval.^37^ Recent efforts to improve patient adherence have focused on the development of long-acting injectables to increase the dosing interval, maintain drug concentrations within active therapeutic limits, reduce fluctuations associated with oral dosing, improve patient quality of life and potentially reduce some of the side effects experienced with oral dosing. There is also an urgent need for more discrete HIV/AIDS treatment and prevention strategies, with several at-risk groups preferring long-acting formulations, including injections, to daily dosing with tablet(s).^40,41^ In January 2021, ViiV Healthcare’s Cabenuva (rilpivirine, cabotegravir) became the first Food and Drug Administration (FDA)-approved intramuscular long-acting injectable for HIV treatment in adults.^42^ This was shortly followed by the licensing of long-acting Apretude (cabotegravir) suspension for HIV prevention in at-risk groups because of its superiority compared to daily oral PrEP in reducing HIV incidence in randomized clinical trials (HIV Prevention Trials Network [HPTN] 083 and HPTN 084).^43^

The development of long-acting formulations tends to focus on the use of: water-insoluble drugs as suspensions in which drug particles are suspended in an aqueous medium;^44^ microspheres were drugs are encapsulated within biodegradable polymers;^45^ oil-based injections whereby hydrophobic drugs are dissolved in an oily medium and precipitate upon injection;^46^ or preformed implants composed of nonbiodegradable polymers.^47^ While successfully implemented clinically, there are several challenges to their wider use as long-acting drug delivery platforms. For example, suspension-based approaches can be prone to Ostwald ripening, a process in which particles interact and accumulate to increasing sizes, leading to flocculation and breaking of suspensions.^48^ This process is accelerated by temperature cycling, which means that the worldwide distribution of pharmaceutical suspensions, for example for HIV prevention in sub-Saharan Africa, is more challenging.^49,50^

**Figure 1:**
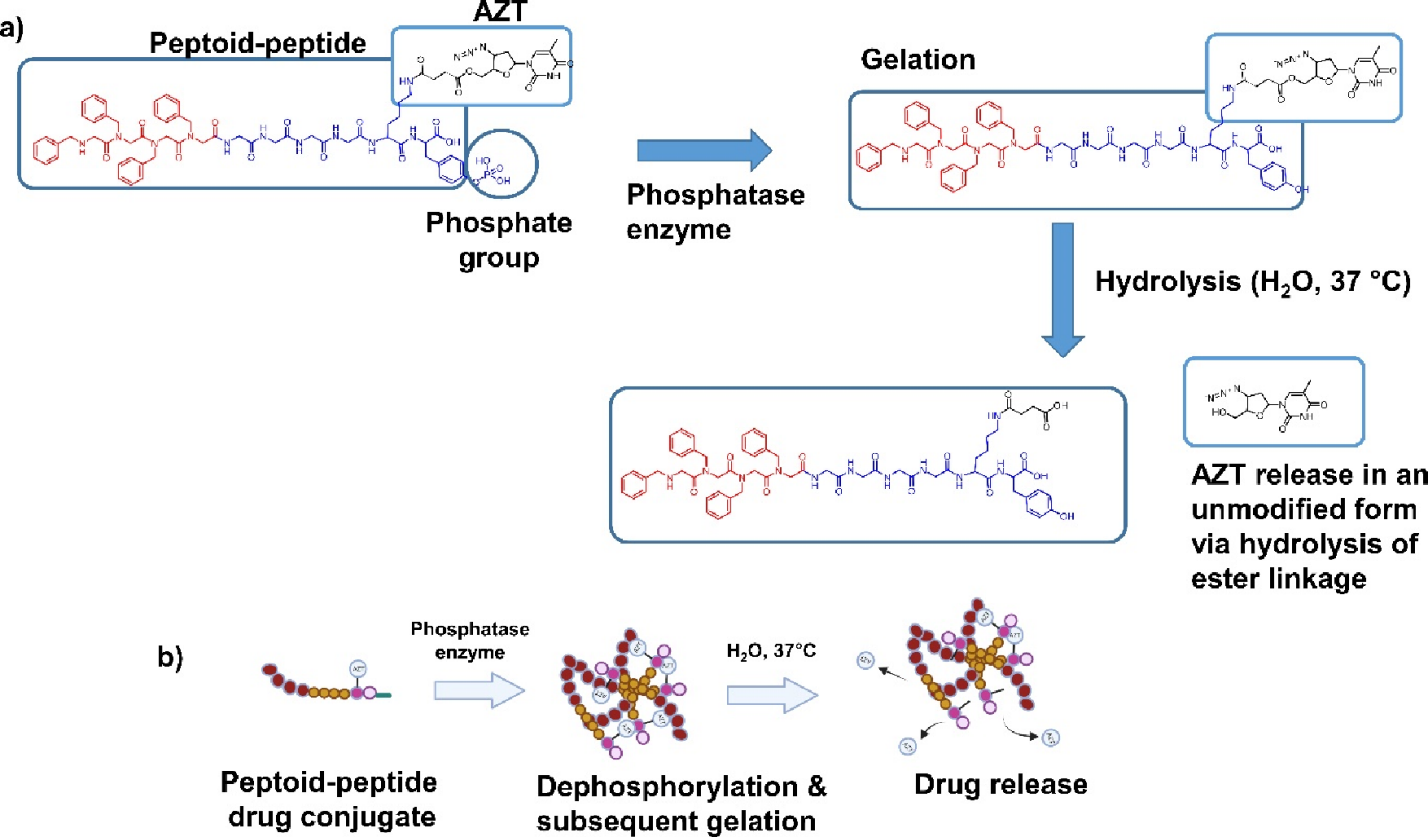
(a) Chemical structure of the peptoid-peptide hydrogelating template. (*N*Phe)_4_GGGGKY(p)-OH was covalently attached to the antiretroviral drug zidovudine (AZT), via an ester linkage at the lysine (K) position. The addition of the endogenous enzyme phosphatase results in the removal of the attached phosphate group (p) by dephosphorylation at the tyrosine position (Y), enabling gelation to occur. Hydrolysis of the drug-ester linkage enables the drug (AZT) to be released in an unmodified form. (b) A representation of the assembly of peptoid-peptide monomers into a cross-linked hydrogel network via the removal of the phosphate group, enabling sustained release of the drug by hydrolysis of the drug-ester linkage into the surrounding environment.

This paper aims to develop an alternative long-acting drug delivery platform to overcome some of these formulation issues, providing wider choice and the potential to tune the chemical structure to functional requirements. One major advantage of peptide and peptide-mimetic hydrogels is that they are amenable to manipulation at the molecular scale relative to standard formulation approaches and synthetic polymers, allowing them to tune features such as drug release and viscoelastic and mechanical properties.^2^ This approach led us to develop a peptoid-peptide drug delivery system with the aim of developing a fully soluble formulation capable of undergoing enzyme-responsive hydrogelation *in situ*, triggered by the presence of endogenous phosphatase enzymes within the subcutaneous skin space (Figure 1), creating a drug releasing hydrogel depot system. The inclusion of L-α or d-lysine provides a primary amino group that facilitates the precise covalent attachment of drug(s) via a labile ester linkage, enabling drug(s) to be released in an unmodified form under physiological conditions via hydrolysis of the drug-ester bond. We hypothesized that chemical attachment of a drug alongside a diffusion barrier provided by rapid hydrogel formation would reduce the initial drug burst. This work serves as a proof-of-concept for the use of non-native peptide-mimetic peptoid-peptide hybrids as a novel long-acting drug delivery platform for the systemic delivery of low-molecular-weight drugs.

## 2 Results and Discussion

The properties characterized below provide important proof-of-concept data for the development of a peptoid-peptide drug delivery system as a new class of *in situ* forming long-acting injectable. Compared with synthetic peptides and alternative peptide-mimetic approaches e.g., D-peptides, β-homo and γ-peptides,^12,15,16^ peptoids are easier and less expensive to synthesize. Therefore, these compounds are attractive molecules for upscaled manufacture and clinical translation as pharmaceuticals. The solid-phase sub monomer method used to synthesize peptoids enables *N*-substituted glycines to be conjugated sequentially, allowing “bottom-up” control over the monomer sequence, overall chain length, side-chain chemistry and functional properties.^51−53^ Additionally, these materials hold promise as alternative hydrogel platforms for wider industrial applications.^54^ The following results outline the creation of an initial peptoid-peptide motif (*N*Phe)_4_(*N*Lys)Y(p)-OH, which evolved into the final peptoid-D-peptide molecule (*N*Phe)_4_GGGGky(p)-OH, which forms hydrogels in response to phosphatase enzymes and demonstrates superior biostability for long-acting drug delivery applications. Our initial goal for this technology was to develop a system capable of providing prolonged drug release for at least 28 days.

### 2.1 Peptoid-Peptide Synthesis, Drug Conjugation, Purification, Identification, and Formulation

The original designed sequence was composed predominantly of peptoid monomers with a single phosphorylated l-tyrosine, (*N*Phe)_4_(*N*Lys)Y(p)-OH. Our original aim was to develop a novel peptoid-based platform that could form supramolecular hydrogels in response to phosphatase enzymes. To our knowledge, a peptoid-only hydrogel does not exist in the literature. Phosphorylating an *N*Tyr to create (*N*Phe)_4_(*N*Lys)(*N*Tyr(p))-OH was not possible to efficiently synthesize to high purity. Peptoids have previously been demonstrated to self-assemble into nanosheets via hydrophobic interactions and electrostatic interactions between side chains. These forces were not sufficient to drive the formation of supramolecular hydrogels, likely due to the reduced flexibility and increased steric bulk of the peptoids relative to their corresponding peptide sequence.^30^ Low-molecular-weight tripeptoid “organo hydrogels” have been recently demonstrated to form using polar organic solvents such as dimethyl sulfoxide (DMSO) and methanol in combination with water.^55^ Given the range of phosphorylated L-, D-, and β-peptide hydrogels that form due to the kinetics of enzyme-instructed self-assembly,^56,57^ we believed that gelation may be possible by using a predominantly peptoid molecule. However, (*N*Phe)_4_(*N*Lys)Y(p)-OH was unable to form hydrogels in the presence of 3.98 U mL^–1^ of alkaline phosphatase enzyme up to 5% w/v (Table S1). Therefore, a series of sequentially modified peptoid-peptides with increased rotational flexibility and reduced steric bulk were synthesized. Additional peptoid-peptides were subsequently synthesized to increase the presence of glycine (G) residues, (*N*Phe)_4_(*N*Lys)Y(p)G-OH and (*N*Phe)_4_(*N*Lys)Y(p)GGG-OH (Table S1). Glycine was chosen as the simplest amino acid, it possesses a single hydrogen atom as its side chain, providing a high degree of flexibility and reduced steric hindrance.^58^ Several studies, including a seminal paper from the Stupp group,^59^ have utilized multiple (≥3) glycine as a flexible linker region between more hydrophilic head groups (e.g., KY(p)-OH) and a more rigid hydrophobic sequence region (e.g., *N*Phe_4_).^60−62^ The inclusion of glycine within low molecular weight peptide/peptide-like systems has also demonstrated an improved propensity for the gelation due to a higher degree of rotational flexibility compared to other amino acids due to decreased steric bulk.^62,63^

Hydrogel formation was achieved by replacing *N*Lys with L-α lysine (K) and separating the peptoid and peptide portions with four glycine residues, creating the peptoid-L-peptide (*N*Phe)_4_GGGGKY(p)-OH. The lysine residue offers an amino side chain, providing a functional group for precise drug conjugation. The inclusion of a phosphate group (p) on the tyrosine moiety acts as an enzymatic trigger for gelation *in vivo* once dephosphorylated by endogenous phosphatase enzymes. A peptoid-peptide sequence in which l-tyrosine and l-lysine were switched with their D-amino acid enantiomers was synthesized ((*N*Phe)_4_GGGGky(p)-OH) in order to study whether enhanced proteolytic stability could be obtained. The model low-molecular-drug weight drug zidovudine (AZT) was then attached to peptoid-L-peptide and peptoid-D-peptide. The purity of the products was analyzed by HPLC (>95%, Figures S19–S22) and each molecule was identified and confirmed via ^1^H NMR (Figures S2–S8) and electrospray ionization (ESI) mass spectrometry (Figures S16–S18). The retention of the phosphate group after synthesis was confirmed by ^31^P NMR (Figures S9–S15).^64^

### 2.2 Gelation Propensity and Mechanical Characterization

Our drug delivery system relies on enzyme-instructed self-assembly to drive supramolecular hydrogel formation, triggered by dephosphorylation of the phosphate group on the peptoid-peptide by phosphatase enzymes. In practice, a drug-releasing hydrogel depot should be formed within the subcutaneous skin space.^63^ Several concentrations (10–30 U L^–1^),^65^ (0.02 U mL^–1^, 1 U mL^–1^, and 20 U mL^–1^),^64,66^ of alkaline phosphatase have been studied to afford enzyme-responsive gelation of phosphorylated peptide precursors. We chose a stock solution of 1000 U mL^–1^ alkaline phosphatase enzyme, with the addition of 2 μL to each peptoid-peptide solution equivalent to 2 U of enzyme in 502 μL or 3.98 U mL^–1^ (Table S2).^63^

The ability of each synthesized sequence to form hydrogels was initially screened by a vial inversion assay. This approach also provided a general assessment of the critical/minimum gelation concentration (% w/v) and is a quick test of a formulation’s propensity to form hydrogels (Table S1).^2^ The initial sequence, (*N*Phe)_4_(*N*Lys)Y(p)-OH (Figure S1a), did not form hydrogels up to 5% w/v in response to the addition of alkaline phosphatase enzyme. Similarly, the following peptoid-peptide conjugates did not form hydrogels at concentrations up to 5% w/v; when a single terminal glycine was added to (*N*Phe)_4_(*N*Lys)Y(p)-OH (Figure S1a) to form (*N*Phe)_4_(*N*Lys)Y(p)G-OH (Figure S1b), and when three terminal glycines were attached to (*N*Phe)_4_(*N*Lys)Y(p)-OH (Figure S1a) to form (*N*Phe)_4_(*N*Lys)Y(p)GGG-OH (Figure S1c). Gelation was not observed for any of these sequences, likely owing to the reduced ability of the peptoid-peptide sequences to participate in hydrogen bonding with water (Table S1, Figure S23). Hydrogel formation relies on a delicate balance between hydrophilicity (water solubility) and hydrophobicity (water insolubility).^67^ It was hypothesized that sequentially increasing the number of glycine residues within the peptoid-peptide sequence would provide a means to achieve such balance by acting as a flexible spacer between the bulky hydrophobic (*N*Phe)_4_ and more hydrophilic KY(p)-OH sequence.^59,68^

Wu and colleagues previously reported that the ability of peptoid sequences to form supramolecular hydrogels in water could be improved by the addition of the short tripeptide sequences RGD, YSV, VPP and GGG. The tetra-peptoid sequence (*N*Phe)_4_ linked to tripeptides formed hydrogels via a heating–cooling process in pH 7.4 PBS at a concentration of 1% w/v.^31^ This work also led us to unsuccessful hydrogel formation via a similar heating (70 °C) – cooling (25 °C) formulation step prior to repeated administration of 3.98 U mL^–1^ alkaline phosphatase enzyme. We used this approach for peptoid-peptide molecules that failed to originally demonstrate propensity to gelate (Table S1). The difficulty in designing peptoid-only hydrogel sequences has been recently highlighted by the Lau and Tuttle groups.^69^ They utilized peptoid computational dynamics in order to discover the minimal di- or tripeptoid sequence that may inform self-assembly and nanostructure formation, as successfully outlined previously for tripeptides.^70^ The tripeptoid Nf-Nke-Nf demonstrated the ability to form nanofibers similar to the nanotubes of the dipeptide FF,^71^ but no hydrogel formation was observed.

**Figure 2:**
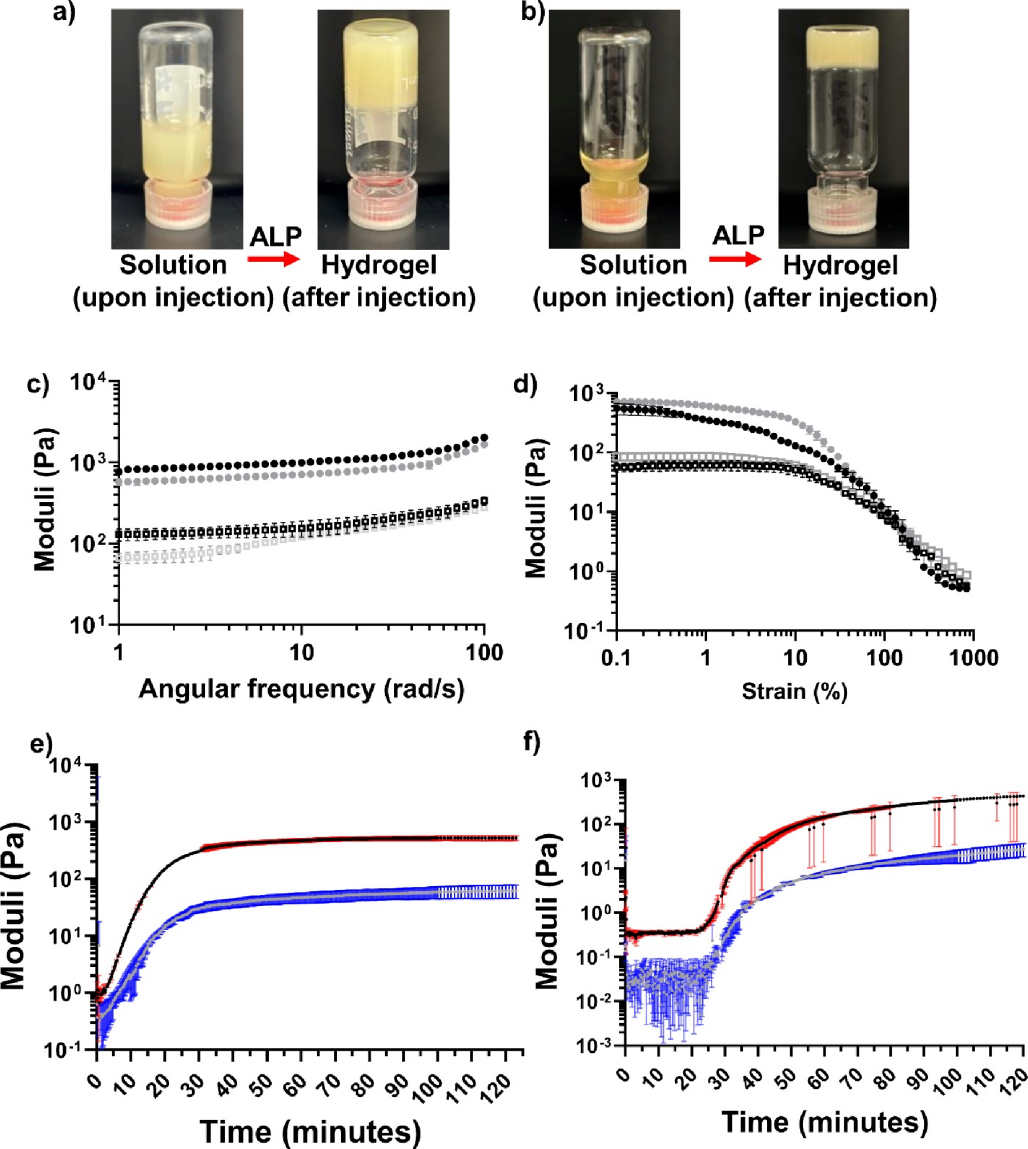
Vial inversion assay for (a) peptoid-D-peptide 2% w/v (*N*Phe)_4_GGGGky(p)-OH and the peptoid-D-peptide drug conjugate (b) 5% w/v (*N*Phe)_4_GGGGk(AZT)y(p)-OH in response to alkaline phosphatase (ALP) activity, providing an initial assessment of the propensity to form hydrogels. (c–f) Several important rheological data relating to peptoid-D-peptides, 5% w/v (*N*Phe)_4_GGGGky(p)-OH and 5% w/v (*N*Phe)_4_GGGGk(AZT)y(p)-OH. Means ± standard deviations (SDs) plotted for each (*n* = 3). (c) Frequency sweeps for 5% w/v peptoid-D-peptide hydrogels. (d) Strain sweeps for 5% w/v peptoid-D-peptide gels. In (c) and (d), the peptoid-D-peptide (*N*Phe)_4_GGGGky-OH is presented in black, and the peptoid-D-peptide with zidovudine-attached (*N*Phe)_4_GGGGk(AZT)y-OH is presented in gray. The filled circles represent the storage modulus (*G*′), and the open squares represent the loss modulus (*G*″). Rheological time sweeps to 120 min for (e) 5% w/v (*N*Phe)_4_GGGGky(p)-OH and (f) 5% w/v (*N*Phe)_4_GGGGk(AZT)y(p)-OH. In (e) and (f), the black lines represent the storage modulus (*G*′), and the gray lines represent the loss modulus (*G*″) with the red and blue areas donating SDs for *G*′ and *G*″ respectively. The full results relating to rheological analysis outlined in Figures S25–S28 and Table S3.

Wang and colleagues performed studies examining the position and number of glycine residues and the effect that this modification had on gelation of the peptide sequence NapFFY(p)-OH.^62^ They found that placing a glycine residue between Nap and FF in the sequence reduced the minimum gelation concentration and proposed that this was due to glycine acting as a linker to separate the bulky groups of Nap and FF. The authors also varied the number of glycine residues (between one and four) within the sequence, which influenced the gelation ability, with an odd number of glycines producing gels at a lower minimum gelation concentration. The authors attributed this to more efficient molecular packing of the peptide derivatives. The position of glycine residues is therefore important, and the sequence was further modified to place four glycine residues between the peptoid and peptide portions of the sequence as a flexible linker, thereby creating the peptoid-L-peptide (*N*Phe)_4_GGGGKY(p)-OH. This demonstrated a propensity to gelate, even after the covalent attachment of the drug (AZT), because of the presence of additional glycines and possibly due to the separation of the bulky portion (*N*Phe)_4_ and hydrophilic KY(p)-OH moiety (Figures S24). A peptoid-D-peptide was subsequently produced (*N*Phe)_4_GGGGky(p)-OH, which was hypothesized to improve proteolytic resistance and biostability, important attributes for long-acting drug delivery. Both peptoid-L-peptide and peptoid-D-peptides demonstrated a minimum gelation concentration of 2% w/v (Figure 2a). A concentration of 5% w/v improved the rheological characteristics (Figures 2b–f, S28, Table S3), this higher concentration was therefore selected for further characterization, i.e., *in vitro* and *in vivo* drug delivery.

A potential area for future study would be to correlate dephosphorylation to gelation propensity, to see if lack of gelation relates to low dephosphorylation conversions or supramolecular interactions.^72^ This has been previously studied by the Xu and Ulijn groups using LC-MS, albeit for peptides where hydrogel formation has been established and in solution, at concentrations below their respective minimum gelation concentrations e.g. 500 μM.^73,74^ A similar study at relevant hydrogel forming concentrations (2–5% w/v) may also provide insight as to how dephosphorylation and supramolecular gelation effects ester hydrolysis and drug release.^75,76^

Oscillatory rheological assessment is an important tool for demonstrating hydrogel formation and characterizing the mechanical properties important for injectable *in situ* forming hydrogel depots. Our drug delivery system is partly defined by drug diffusion from the hydrogel matrix, and hydrolysis of the peptoid-peptide-drug chemical linker. Reproducing supramolecular gels’ mechanical properties is difficult, especially for peptide-based systems. Experimental parameters and formulation factors are particularly important, as highlighted recently by the Adams and Draper groups.^77,78^ Subtle experimental influences e.g. handling and temperature, can lead to issues with reproducibility. Gelation behavior was readily reproducible for our peptoid-peptide systems (*n* = 3) so long as the formulation steps of Table S2 and outlined rheological methods are closely followed (Supporting Information, Section S.4.). Loss (*G*″) and storage (*G*′) moduli, critical strain (%) and oscillatory time sweeps were performed to determine the rheological changes with time and the potential for rapid *in situ* hydrogel depot formation upon exposure to 3.98 U mL^–1^ phosphatase enzyme. Rheological measurements of the original peptoid-L-peptide (*N*Phe)_4_GGGGKY(p)-OH motif are outlined in Figure S25. Hydrogel formation occurs when the storage modulus (*G*′) is at least 1 order of magnitude greater than the loss modulus (*G*″).^79^ This was observed for frequency sweeps conducted within the linear viscoelastic region (LVR) for both peptoid-L-peptides (Figure S25a) and peptoid-D-peptides (Figure 2c) with and without covalent attachment of zidovudine. This indicated good viscoelasticity for the formed hydrogels. The dynamic frequency sweeps also demonstrated that the moduli were largely independent of the frequency applied for peptoid-L-peptides (Figure S25a), although a slight frequency dependence was observed for peptoid-D-peptides, suggesting that a weak elastic matrix may exist within these hydrogels.^80^

Analysis of frequency sweeps demonstrated that *G*′ had values spanning an order of magnitude similar to those reported for peptide-based hydrogels,^81^ from ∼650 Pa (2% w/v (*N*Phe)_4_GGGGk(AZT)y(p)-OH) to ∼5.8 KPa (5% w/v (*N*Phe)_4_GGGGKY(p)-OH). Unsurprisingly *G*′ increased with increasing concentration of peptoid-D-peptide (*G*′ = ∼1054 Pa at 2% w/v compared to ∼2030 Pa at 5% w/v). Interestingly peptoid-L-peptide gels without drug were significantly stiffer (Figure S25a), demonstrated through higher *G*′ values, than their peptoid-D-peptide counterparts (Figure 2c). This is different to previous observations for comparing the effect of single L- or d-enantiomers of peptide-based gels. For example, the Xu group reported that the chirality of hydrogelators caused negligible differences in viscoelastic properties, with the l- and d-enantiomeric variants of NapFFKY(p)-OH displaying similar gel stiffness and strength.^64^ An interesting area for further study would be whether peptoid-L-peptides and peptoid-D-peptides enantiomeric mixtures afford more rigid hydrogels than L and D forms alone, as recently highlighted by the Schneider group with their MAX1 peptide.^82^ As previously observed for low-molecular-weight L-α/D-peptide hydrogels, covalent attachment of the model drug zidovudine via an ester linkage significantly reduces gel stiffness for both peptoid-L-peptides and peptoid-D-peptides.^63^ The attachment of zidovudine to the peptoid-peptides and the subsequent formulation of this gel may have resulted in the formation of different types of entanglements within the gel network. The attachment of drugs may interfere with intra- and intermolecular bonding, therefore impacting molecular packing and the nature of the hydrogel network as observed previously by Chen and colleagues.^83^ In this study, the covalent attachment of ketoprofen to a low molecular weight tetrapeptide hydrogelator interfered with intra- and intermolecular bonding. Upon assembly, ketoprofen’s two hydrophobic phenyl groups were present within the predominantly hydrophilic plane of the tetra peptide sequence, disrupting molecular packing and reducing the hydrogel’s mechanical properties.

**Figure 3:**
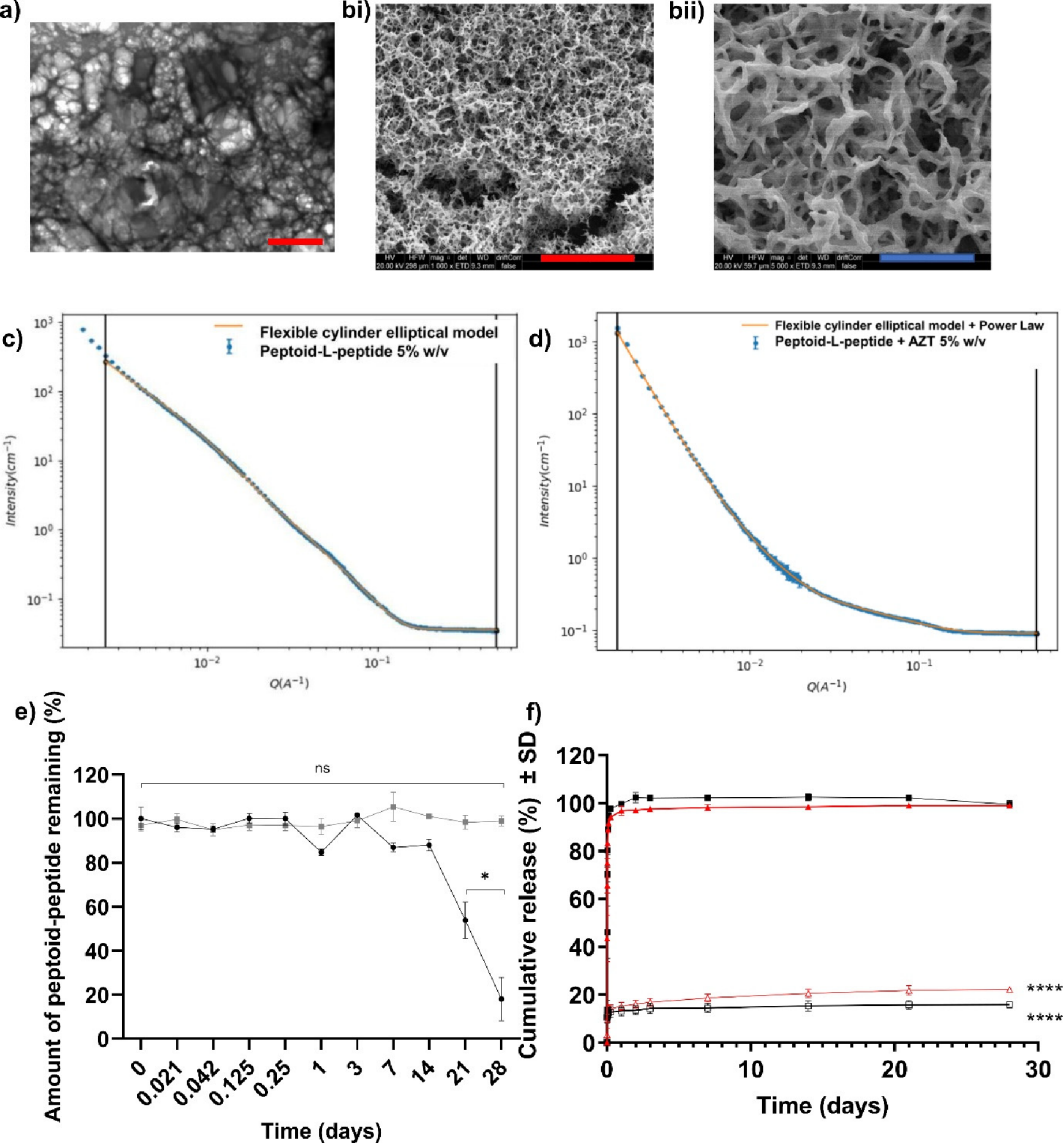
(a) TEM image showing the fibrous architecture of 5% w/v (*N*Phe)_4_GGGGk(AZT)y-OH at 2000× magnification; red scale bar = 5 μm. (b) SEM images (HV = 20 kV) showing the fibrous architecture of 5% w/v (*N*Phe)_4_GGGGKY-OH hydrogels at (i) 1000× magnification and (ii) 5000× magnification; red scale bar = 100 μm, blue scale bar = 20 μm. (c,d) SANS data for 5% w/v peptoid-L-peptide hydrogels, (c) (*N*Phe)_4_GGGGKY-OH and (d) (*N*Phe)_4_GGGGK(AZT)Y-OH (dotted line). The solid line represents the model data for the flexible cylinder elliptical model alone (c) and for the case in which the power law was applied (d). Scattering data were collected over a wide Q range [Q = 4πsin(θ/2)/λ] of 0.001 to 0.5 Å^–1^ and three sample–detector distances (1.4 m, 8 m, 39 m). (e) The biostability of the parent peptoid-L-peptide (*N*Phe)_4_GGGGKY(p)-OH (black filled circles) and peptoid-D-peptide (*N*Phe)_4_GGGGky(p)-OH (gray filled squares) after incubation with the broad-spectrum protease proteinase K for 28 days. The values represent means ± SDs (*n* = 3). Key: ns: no significant difference (*p* > 0.05); *p ≤ 0.05 difference between the proteinase K-treated peptoid-L-peptide (*N*Phe)_4_GGGGKY(p)-OH and the negative, nontreated, peptoid-peptide only control. (f) Cumulative percentage (%) of zidovudine (AZT) released from physically encapsulated and chemically conjugated (*N*Phe)_4_GGGGKY-OH and (*N*Phe)_4_GGGGky-OH in PBS over 28 days (*n* = 3). Key: black filled squares (*N*Phe)_4_GGGGKY-OH + AZT (peptoid-L-peptide, drug physically mixed), black unfilled squares (*N*Phe)_4_GGGGK(AZT)Y-OH (peptoid-L-peptide, chemically conjugated drug), red filled triangles (*N*Phe)_4_GGGGky-OH + AZT (peptoid-D-peptide, drug physically mixed), red unfilled triangles (*N*Phe)_4_GGGGk(AZT)y-OH (peptoid-D-peptide, chemically conjugated drug). ****: *p* ≤ 0.0001 difference between drug release from zidovudine chemically conjugated to the peptoid-peptide hydrogel and the respective physically encapsulated control.

Rapid gelation is important for *in situ* formation of drug delivery depots to reduce the diffusion of drug(s) from the depot and potential burst release. Quick gelation after injectable administration is also important for our system in order to reduce the availability of the peptoid-peptide ester linkage with the drug to water within the subcutaneous space, providing a diffusion barrier to rapid drug cleavage by hydrolysis.^84^ There are several ways in which time for gelation can be defined: a) the time at which the storage modulus (*G*′) and loss modulus (*G*″) cross indicating greater solid-like behavior; b) the time point at which *G*′ is greater by 1 order of magnitude than *G*″; and c) the time at which *G*′ and *G*″ begin to stabilize/plateau. Based on a) and b) at 5% w/v (*N*Phe)_4_GGGGKY(p)-OH, gelation of the peptoid-L-peptide complex begins within 10 s (first observable time point) after the addition of phosphatase enzyme (Figures S25c), stabilizing at ∼80 min and forming a gel with a stiffness of 5064.35 Pa after 250 min (Figure S25e, Table S3). Quick gelation was also demonstrated for 5% w/v (*N*Phe)_4_GGGGK(AZT)Y(p)-OH (Figure S25d). The attachment of drug caused an increase in gelation time. *G*′ is an order of magnitude greater than *G*″ after ∼1 min, and reaching a plateau at ∼135 min (Figures S25d, f, Table S3). Peptoid-D-peptides (Figures 2e, 2f, S26c, S27c, Table S3) demonstrated a similar speed of gelation, plateauing ∼70–90 min. The attachment of drug once again increased gelation time by ∼10–20 min in this case. Increased gel stiffness over a longer period of time, i.e., up to 250 min, is likely due to gradual removal of phosphate groups on the peptoid-peptide structure. As previously observed with phosphorylated peptide hydrogels,^63,85^ removal of all phosphate groups is not required to achieve gelation; rather, the gel stiffness increases over time with continued removal of phosphate groups and possible organization of fiber entanglements, as observed in the SANS data (Figure 3c, d), and/or with the formation of additional networks or entanglement of the initial gel network.

Evidence of at least two networks can be identified in the (*N*Phe)_4_GGGGK(AZT)Y-OH strain sweep where the deformation of the gel occurs at both ∼4% and ∼17.5% strain (Figure S25b). This would imply that the weaker network breaks under ∼4% strain whereas the stronger second network or entanglement breaks at ∼17.5% strain. This second network seems to also exist for peptoid-L-peptides alone ((*N*Phe)_4_GGGGKY-OH: ∼5.78% and ∼36.5% strain) and for the peptoid-D-peptide variants with (∼1.1% and ∼8.36% strain) and without (∼0.527% and ∼4.81% strain) drug (Figure 2d).^83^

In our previously reported work studying low-molecular-weight D- and L-peptide hydrogels, the conjugation of zidovudine to peptide sequences resulted in an increase in gel strength measured by the breakage strain/flow point.^63^ This was not observed for the peptoid-peptide-AZT conjugates. Complete deformation, where *G*′ and *G*″ intersect, was observed at a lower strain (∼44%) for drug-attached peptoid-L-peptide (*N*Phe)_4_GGGGK(AZT)Y-OH (Figure S25b) than peptoid-L-peptide alone (∼92%). Complete deformation was higher for peptoid-D-peptide. Both peptoid-D-peptide alone and peptoid-D-peptide-AZT gel networks were disrupted at a strain of ∼192%.

### 2.3 Microscopy and SANS

Imaging techniques, such as SEM and TEM in combination with SANS, are complementary techniques for revealing the detailed structural features within fibrous networks.^86^ Microscopic examination of the peptoid-peptide hydrogels by SEM and TEM revealed a three-dimensional interweaving random entanglement of fibers (Figures 3a, b), consistent with our previous observations for low-molecular-weight peptide hydrogels.^63,87,88^ While microscopy enables fiber structures to be visualized, SANS offers information about the molecular packing and structure of bulk samples at a 1–100 nm scale without the need for drying or the potential formation of artifacts.^89,90^ Currently only the peptoid-L-peptides are studied here, given the demand on beam-time at central facilities such as ILL. Figure 3c shows that 5% w/v (*N*Phe)_4_GGGGKY-OH closely fits the flexible cylinder elliptical model with a fiber radius of 1.825 nm. Figure 3d shows that 5% w/v (*N*Phe)_4_GGGGK(AZT)Y-OH also closely fits the flexible cylinder elliptical model with the power law applied with a narrower fiber radius of 0.781 nm. These findings are similar to previous SANS observations with low-molecular-weight NapFF peptide gels.^89,90^ A summary of the parameters extracted from these fits is outlined in Table S4. SANS spectra demonstrated that the compositions of the gel fibers with and without the addition of the drug zidovudine were similar at low Q (Figures 3c, d). As observed previously with L-α/D-peptide NapFFKY(p)-OH hydrogels,^63^ differences in mechanical/rheological properties are likely driven by changes in how these fibers entangle rather than by changes in their composition or underlying molecular arrangement e.g., secondary structures. The large lengths of these fibers are also evidence of entangled fibers. How these peptoid-peptides are formulated and the gelation process should therefore have a significant impact on the nature of fiber entanglement and the resulting mechanical and functional (e.g., drug release) properties. The design of processes, for example, freeze-drying to a powder formulation and reconstitution in a water-based solvent prior to injection, is likely to be important not only to their future clinical use but also to how these gels form and their fundamental behavior.

### 2.4 Biostability

Proteinase K is utilized as an *in vitro* biostability indicator since it is a broad-spectrum protease against both aliphatic and aromatic peptide-based substrates.^91^ While degradation of peptide and peptide-mimetic-based hydrogels *in vivo* is desired, tailored to the required dosage interval, premature degradation of the hydrogel matrix in the case of long-acting drug delivery applications would result in inappropriate release rates and/or dose dumping.^92^ The data (Figure 3e) demonstrated that the peptoid-L-peptide (*N*Phe)_4_GGGGKY(p)-OH possesses superior biostability relative to L-α peptides, which are well documented to degrade within hours of exposure to proteases. This severely limits the use of L-α peptides in clinical applications, especially within the area of long-acting drug delivery.^93^ The peptoid-L-peptide structure remains stable for the first 2 weeks and then begins to degrade, with 18.1% ± 9.8 remaining at the termination of the study, in line with our initial 28 day dosing interval. The site of degradation is likely the peptide portion of the sequence, which is composed of L-α amino acids. The peptoid-D-peptide (*N*Phe)_4_GGGGky(p)-OH remained stable throughout the duration of the study, with 98.97 ± 2.18% remaining after 28 days. This result demonstrated the promising biostability profile of the peptoid-D-peptides, suggesting that it may be possible to extend the dosage interval beyond the initial 28 days for this platform. For any future clinical application, tailoring the degradation profile, by modifying the peptoid-peptide chemical structure to the drug release rate observed in animal and human studies and the required dosage interval, will be important.

### 2.5 *In Vitro* Drug Release

The *in vitro* drug release profiles displayed in Figure 3f consist of an initial burst release and then a plateau with slower drug diffusion through the hydrogel matrix, which is typical of *in situ* forming depots.^94^ A large amount of drug burst release was observed upon physical encapsulation of zidovudine in combination with (*N*Phe)_4_GGGGKY-OH and (*N*Phe)_4_GGGGky-OH, with 102.2% and 97.5%, respectively, of the loaded drug being released in the first 72 h. This is typical of physically encapsulated drug release systems, which are prone to drug leakage and a high burst release.^8,95^ The release profile was monitored over the entire 28 day profile. As the majority of the drug load is spent within the first 72 h for physically encapsulated forms, clinical application would likely result in an increased risk of initial toxicity and subtherapeutic levels of drug for the remainder of the dosing interval. Physically encapsulated systems are more applicable to acute drug delivery applications where rapid treatment, such as pain relief, is required at a high dose for a shorter time period. Zidovudine is conjugated to the lysine residue of each peptoid-peptide via an ester linkage. Drugs are released from the hydrogel systems in an unmodified form via simple hydrolysis of the drug-ester linkage under physiological conditions. In practice, diffusion through the peptoid-peptide hydrogel matrix and into the surrounding fluid would enable uptake into the systemic circulation. Figure 3f shows that burst release still persists from the chemically conjugated systems but is significantly lower than that from physical encapsulation. The drug release for (*N*Phe)_4_GGGGKY-OH was reduced by approximately ∼88% (from 102.2% to 14.2%) within the first 72 h. A similar trend was observed for (*N*Phe)_4_GGGGky-OH, in which drug release in the first 72 h was reduced by 81% (from 97.5% to 16.9%). At the final time point (28 days), only ∼20% of the drug had been released in peptoid-peptide formulations were zidovudine was covalently attached (15.9% peptoid-L-peptide, 22.3% peptoid-D-peptide). This indicates that a longer dosing interval may be achieved; however, release rates *in vivo* are likely to increase; therefore, there is an obvious need to conduct preliminary studies in small animals and follow-on pharmacokinetic studies in large animals, e.g., macaques and clinical trials in humans. There appears to be a link between gelation times for peptoid-L-peptide and peptoid-D-peptide hydrogels, defined by rheological time sweeps (Figures 2f, S25c–f and Table S3). The majority of % cumulative drug released for both (*N*Phe)_4_GGGGK(AZT)Y-OH (11.04%) and (*N*Phe)_4_GGGGk(AZT)y-OH (9.5%) is within the first 30 min when the peptoid-peptides are beginning to gel and both *G*′ and *G*″ are stabilizing *in situ*. It is well established that rapid gel formation minimizes burst release of drug and this is especially relevant to our system,^96^ whereby the presence of a gel should act as a diffusional barrier, reducing the ability of water to hydrolyze the covalent ester linkage between peptoid-peptide and drug.

KinetDS software was utilized to further understand the drug release kinetics of these novel peptoid-peptide hydrogel platforms. Various kinetic models, including zero-order, first-order, Korsmeyer–Peppas, Hixson–Crowell, Higuchi and Weibull models were applied to determine the mechanism of drug release. The respective *r*^2^ values obtained from model fitting are shown in Table S5 for peptoid-L-peptides and peptoid-D-peptides with zidovudine (i) encapsulated and (ii) covalently attached via an ester linkage. For injectable hydrogel-forming depots quantified by cumulative release, zero-order, first-order and Korsmeyer–Peppas models tend to be the most relevant for establishing the mechanism of drug release kinetics. The Higuchi model applies to drug release driven mainly by diffusion only and it is accepted that this model should not be used when hydrogel swelling may be a factor in release.^97−99^ Hixson–Crowell is generally not suited for hydrogels and is applied to drug delivery systems that undergo significant changes in diameter and surface area e.g., from particles and tablets.^100^ The Weibull model demonstrated the highest *r*^2^ values, followed closely by Korsmeyer–Peppas for covalently attached drugs in the peptoid-L-peptide (*N*Phe)_4_GGGGK(AZT)Y-OH and peptoid-D-peptide (*N*Phe)_4_GGGGk(AZT)y-OH; the *r*^2^ values were 0.84 and 0.86 for both the Weibull and Korsmeyer–Peppas models, respectively, in these systems. Korsmeyer–Peppas tends to be more relevant to three-dimensional drug delivery systems such as peptoid-peptide hydrogels and warrants a stronger case for support. The Weibull model is associated more with nanoparticles and heterogeneous formulations; however its application in model injectable hydrogels is increasing.^101,102^ According to the Korsmeyer–Peppas model (Table S6), the diffusion exponent *n* is slightly greater than 1 for physically encapsulated drug forms of peptoid-L-peptide (*N*Phe)_4_GGGGK(AZT)Y-OH and peptoid-D-peptide (*N*Phe)_4_GGGGk(AZT)y-OH and is slightly less than 1 for chemically attached drug forms. This indicates super case II transport (*n* > 0.85), whereby the drug is mainly released through the erosion of the polymer matrix and/or possibly hydrolysis of the ester-drug linkage where present.^99,103^ Super case II transport has been previously demonstrated for Fmoc-diphenylalanine peptide hydrogels physically encapsulated with the nonsteroidal anti-inflammatory drug (NSAID) indomethacin.^104^ When the Weibull model is applied, the value of the shape factor β is most useful for providing information on the mechanism of drug release. The β values of the drug-conjugated peptoid-peptides hydrogels were calculated to be between 0.75 and 1 ((*N*Phe)_4_GGGGKY-OH β = 0.9550, (*N*Phe)_4_GGGGk(AZT)y-OH β = 0.9748) indicating a combination of gradual drug release by diffusion (Fickian diffusion) and hydrogel erosion/polymer relaxation (case II transport).^101^ For the drug-encapsulated peptoid-peptides hydrogels (Table S7), both (*N*Phe)_4_GGGGKY-OH (β = 1.169) and (*N*Phe)_4_GGGGky-OH (β = 1.088) possessed β values of >1, indicating a more complex combined drug release mechanism.^102,105^

### 2.6 Cell Cytotoxicity

**Figure 4:**
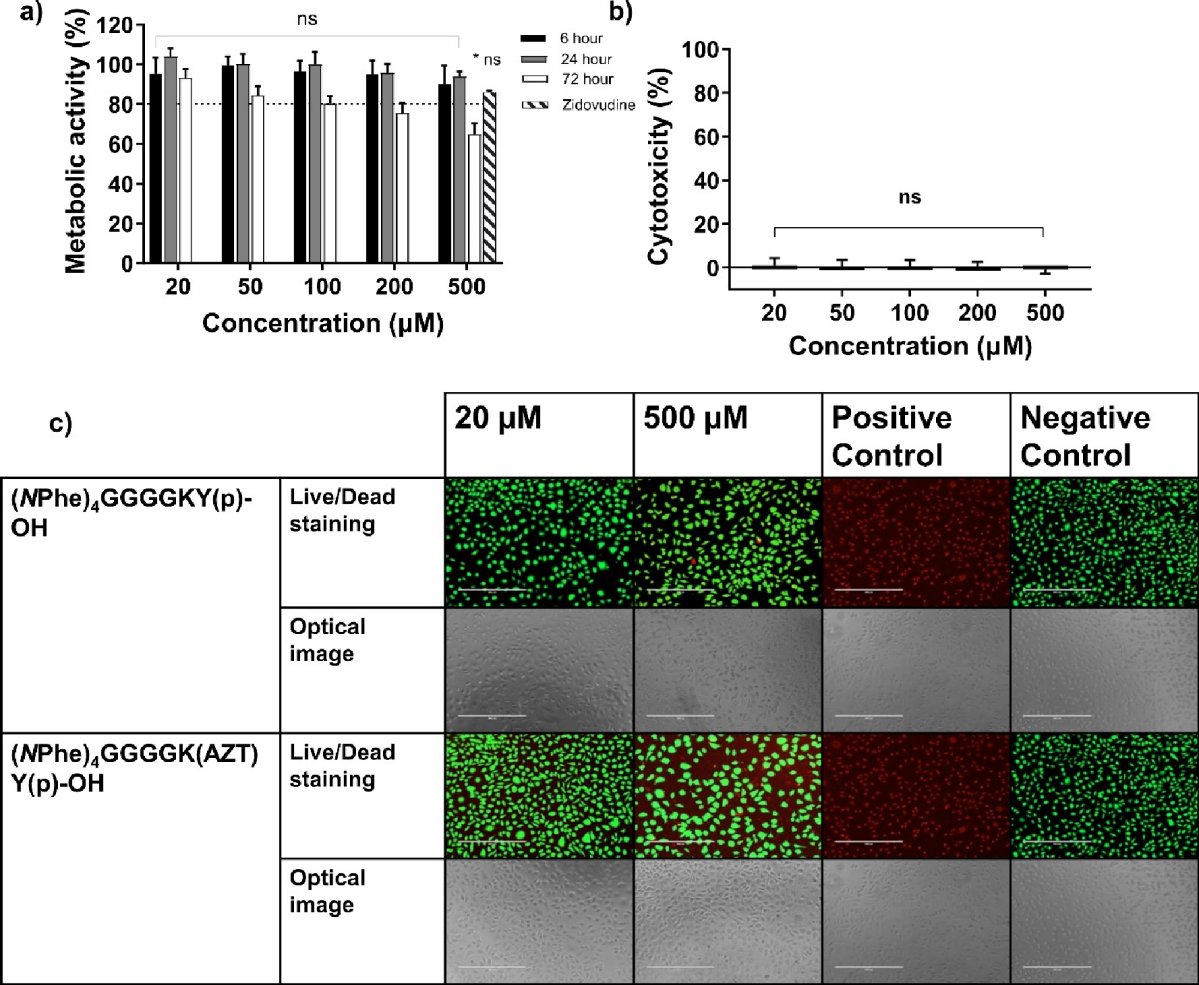
Cellular cytotoxicity of fully solubilized (*N*Phe)_4_GGGGK(AZT)Y(p)-OH was measured using (a) a MTS viability assay (6, 24, and 72 h), (b) a LDH toxicity assay (6 h) and (c) Live/Dead staining of (*N*Phe)_4_GGGGKY(p)-OH and (*N*Phe)_4_GGGGK(AZT)Y(p)-OH (24 h, scale bar: 400 μm). The means ± SDs are provided for nine replicates in (a) and (b). ns: not significant (*p* > 0.05), *p ≤ 0.05; **p ≤ 0.01; ***p ≤ 0.001; ****p ≤ 0.0001, difference between the peptoid-peptides and the negative control (media only). The cytotoxicity study utilized 70% v/v ethanol as the positive control (100% kill).

The *in vitro* cell cytotoxicity profiles of the peptoid-peptides were tested using three separate tests (i) MTS, (ii) LDH and (iii) Live/Dead staining assays. Fully solubilized peptoid-peptide formulations were tested at concentrations ranging from 20–500 μM for up to 72 h. The majority of the assays demonstrated a trend whereby peptoid-peptides displayed no significant cytotoxicity up to 500 μM. This is similar to the cytotoxicity profile for other solubilized peptides capable of forming hydrogels.^32,63,92,106^ According to the results of the MTS cell viability assay, treatment with 500 μM peptoid-D-peptide (*N*Phe)_4_GGGGky(p)-OH significantly reduced cell metabolic activity at all time points (6, 24, and 72 h, Figure S30). Treatment with 500 μM peptoid-L-peptide (*N*Phe)_4_GGGGKY(p)-OH significantly reduced cell metabolic activity only after 72 h of exposure to NCTC 929 cells (Figure S29). This trend was supported by results of the Live/Dead assay conducted over 24 h. Peptoid-L-peptide, at 500 μM, had no observable cytotoxic effects on live cells, as determined by the presence of green fluorescent dye (Figures 4c, S33). Peptoid-D-peptide again demonstrated cytotoxicity after 24 h of exposure to 500 μM concentrations, as shown by the presence of red fluorescent stain (Figure S34). The covalent attachment of zidovudine did significantly impact cell metabolic activity, and cell toxicity was once again demonstrated only after 72 h for 500 μM peptoid-L-peptide (*N*Phe)_4_GGGGK(AZT)Y(p)-OH (Figure 4a). These findings were consistent with the results obtained for Live/Dead assays (24 h, Figure 4c) and the cytotoxicity for (*N*Phe)_4_GGGGK(AZT)Y(p)-OH tested via LDH release (6 h, Figure 4b). The LDH assay demonstrated significant cytotoxicity for peptoid-L-peptide (*N*Phe)_4_GGGGKY(p)-OH at all concentrations tested (20–500 μM, Figure S31); however, the cytotoxicity was still below the 20% cell cytotoxicity threshold (80% cell viability) widely employed within research,^107^ and the minimum 70% cell viability value set by the ISO official standards for biomaterial testing.^108^ The peptoid-D-peptide (Figure S32) demonstrated significant toxicity only at 500 μM, in line with the results obtained for MTS assays at the 6 h time point (Figure S30a). The LDH results, particularly those for peptoid-L-peptide, demonstrated the importance in performing more than one assay to determine the cytotoxicity of new chemical entities.

### 2.7 *In Vivo* Plasma Drug Concentration Studies

Sprague–Dawley rats were used as a small animal *in vivo* model to assess the drug absorption, by measuring the plasma concentration of zidovudine, after subcutaneous administration of 5% w/v peptoid-D-peptide (*N*Phe)_4_GGGGk(AZT)y(p)-OH. The peptoid-D-peptide variant was chosen for *in vivo* investigation to confer enhanced resistance to proteolysis (Figure 3e), an important characteristic of long-acting drug delivery platforms.

**Figure 5:**
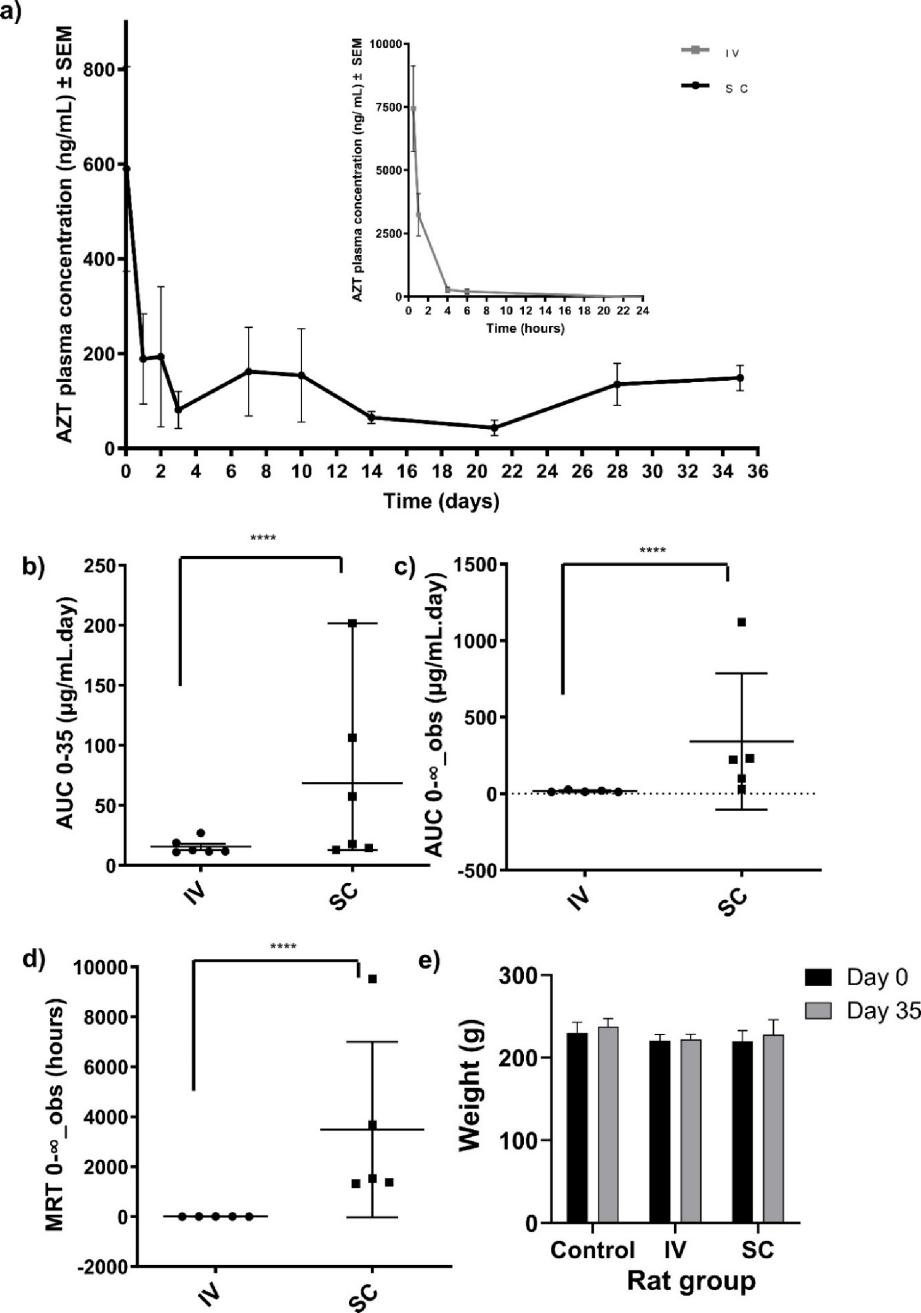
(a) *In vivo* plasma concentration of the drug zidovudine (AZT) measured across 35 days in Sprague–Dawley rats (*n* = 6) after intravenous (IV) administration of AZT and subcutaneous (SC) administration of (*N*Phe)_4_GGGGk(AZT)y(p)-OH. Statistical analysis and representation of (b) AUC_0–35_, (c) AUC_0-∞,obs_ and (d) MRT_0-∞,obs_ in rats from both cohorts. Each point on the graph represents one rat (total *n* = 6 per group). (Key for Figures b–d: ****p ≤ 0.0001). (e) Weights of Sprague–Dawley rats (*n* = 6) at the start (day 0) and the end of the experiment (day 35) in the control, intravenous and subcutaneous administration groups.

Figure 5a shows the plasma concentration of zidovudine over time and additional pharmacokinetic parameters (drug half-life [*t*_1/2_], time to maximum concentration [*T*_max_], maximum concentration [*C*_max_], area under curve [AUC] (Figures 5b, c), and mean residence time [MRT] (Figure 5d)) are presented in Table S8. Figure 5a shows that when zidovudine was administered subcutaneously as part of the hydrogel system, the plasma concentration initially peaked at 590 ng mL^–1^ 1 h after administration. This concentration falls within the first 72 h of treatment with zidovudine, for which the IC_90_ range is 30–130 ng mL^–1^, up to the final time point of 35 days.^109^ Blood plasma concentrations of zidovudine were also found to be within four times its respective IC_90_ value (120 ng mL^–1^), a pharmacokinetic benchmark for HIV protection.^110^ The initial peak in the plasma zidovudine concentration is likely due to burst release from the system upon administration during the gelation process as discussed in section 2.5 and displayed in Figure 3f.

For comparison, intravenously administered zidovudine served as a control. It exhibited a significantly higher plasma peak (7,500 ng mL^–1^), which was 12.5 times greater than the chemically conjugated zidovudine. However, this concentration rapidly diminished to undetectable levels within 6 h. PKSolver 2.0 facilitated the calculation of pharmacokinetic parameters shown in Figures 5b–d and Table S8. Intriguingly, the AUC_0-∞_ for subcutaneously administered zidovudine was approximately 20 times higher than the intravenous route. The *C*_max_ decreased by 17 times, and the MRT was remarkably extended, from approximately 1.58 to 3483.2 h, presenting notable pharmacokinetic advantages. The reduced *C*_max_ minimizes the risk of peak-related toxicities, especially important for drugs with a narrow therapeutic window. Meanwhile, the extended MRT indicates a sustained drug release profile, which is crucial for chronic conditions like HIV/AIDS to maintain consistent drug levels, thereby improving treatment efficacy and patient compliance.^111^ This could lead to reduced dosing frequency, enhancing patient adherence–a significant benefit in lifelong prevention/treatment for diseases such as HIV/AIDS.^112^ These findings are particularly relevant for the development of new drug formulations, where maintaining therapeutic drug concentrations for extended periods is key to therapeutic efficacy. In the context of HIV/AIDS treatment and prevention and diseases with medication adherence issues e.g., psychoses and tuberculosis, a longer dosage interval than 35 days is necessary. For example, Apretude (cabotegravir: HIV prevention) and Cabenuva (rilpivirine, cabotegravir: HIV treatment) are licensed for use and administered at a dosage intervals of every two months.^113^ Therefore, matching or improving this dosage interval would be important from a clinical and commercial perspective and a feasible way to achieve this would be to use drugs with increased potency e.g., cabotegravir with the peptoid-peptide system. There is also the potential to expand the use of this system to other indications, to include several low molecular weight drugs utilized within peptide hydrogels previously, most notably the NSAIDs naproxen, ibuprofen, indomethacin,^7,88^ and the anticancer drugs doxorubicin and taxol.^32,80^ Their compatibility e.g., propensity for gelation, with our peptoid-peptide system would have to be initially established.

During the study, the rats in both the subcutaneous and intravenously administered test groups were monitored for differences in weight and behavior compared to those in a healthy, untreated (negative) control group of rats. All the treatments were well tolerated, with no deaths or serious adverse effects observed and no apparent signs of irritation or infection at the injection site in those receiving intravenous or subcutaneous injections. There were no significant differences in weight between the experimental and control cohorts (Figure 5e), suggesting that the treatments were well tolerated during the 35 day study. These results support our proof-of-concept and suggest that the inclusion of non-native peptoids within the peptide sequence can improve retention, suggesting that this platform has significant potential as an effective long-acting drug delivery system. Given the *in vitro* biostability profile of the peptoid-D-peptide (Figure 3e), it would be prudent to provide insight into its *in vivo* stability and to elucidate the potential host immune response to peptoid-peptide hydrogels,^114^ and possible inflammation at the injection site e.g., erythema, edema,^110,115^ in future work.

## 3 Conclusions

The use of low-molecular-weight peptoid-peptides materials provides a new paradigm for the design and development of peptide-like hydrogels for use in healthcare applications. These materials are especially promising for use as long-acting injectables because of their biostability, ability to undergo *in situ* hydrogelation in response to physiological triggers e.g., enzymes, and capacity to reduce burst/sustained drug release. These characteristics can be built into the chemical structure and design of the peptoid-peptide formulation. Recently, licensed long-acting injectables have focused on the area of HIV/AIDS treatment and prevention. We demonstrated that peptoid-D-peptide can systemically deliver drugs (zidovudine) to rats at clinically relevant concentrations (IC_90_) for 35 days. Therefore, our system holds promise for use not only in HIV/AIDS infection but also in other areas where sustained drug release may be beneficial e.g., oncology, tuberculosis, malaria, ocular and CNS delivery, substance abuse and mental health disorders. Future work will focus on establishing *in vivo* safety/tolerability, efficacy and pharmacokinetics in large animals. For example, in the context of HIV/AIDS, clinically relevant cynomolgus macaque models of SIV can be generated and tested using more potent antiretroviral drugs (cabotegravir), and the ability to upscale manufacture to current Good Manufacturing Practice (cGMP). We envisage clinical peptoid-peptide products to be formulated as powders for injection to provide sufficient pharmaceutical stability and shelf life. Therefore, optimizing the freeze-dried preparation and sterilization of this powder formulation and ensuring that it is readily reconstituted in buffer/water prior to administration as a sterile product will be important. Peptoid-peptides also hold significant promise as new materials for wider use in areas where synthetic and peptide hydrogels are being applied e.g., 3D cell culture, biosensors, wound healing, 3D printing, and stem cell and gene cell delivery.

## 4 Experimental Section

### 4.1 Peptoid-Peptide Synthesis, Drug Conjugation, Purification, Identification, and Formulation

To create a carboxylic acid-terminated peptoid-peptide molecule, 2-chlorotrityl chloride or primarily Wang resin was employed as the solid-phase support. Peptide sequences, e.g., glycine (G), tyrosine (Y) and lysine (K) were incorporated into the peptoid-peptide sequence using standard Fmoc solid-phase protocols.^87^ Solid-phase submonomer synthesis, involving a series of repeated bromo-acetylation and displacement steps was utilized to create the peptoid chain, e.g., (*N*Phe)_4_ sequence, as previously outlined by the Zuckermann group.^51^ The synthetic steps are fully outlined within the Supporting Information (S.1, S.2).

### 4.2 Hydrogel Formulation, Gelation Propensity, and Mechanical Characterization

The propensity for synthesized peptoid-peptide molecules to gelate was tested using a vial inversion assay in HPLC glass vials across a range of concentrations (0.1–5% w/v). When a propensity to gelate was observed, the hydrogel formation and mechanical properties of peptoid-peptides were characterized via oscillatory rheology using an Anton Paar MCR302 rheometer (Anton Paar, St Albans, UK). Methods employed are fully outlined within the Supporting Information (S.3, S.4).

### 4.3 Microscopy

The structure of the formed hydrogels was studied at the nanoscale using scanning electron microscopy (SEM) performed on a JEOL JSM 6500 F SEM (JEOL, Freising, Germany). Microscopy and sample preparation are fully outlined within Section S.3 of the Supporting Information.

### 4.4 Small-Angle Neutron Scattering

Small-angle neutron scattering (SANS) was utilized to probe hydrogel fiber properties at the macroscopic scale and to investigate how molecular packing and long-range networks form for peptoid-L-peptide hydrogels ((*N*Phe)_4_GGGGKY-OH) with and without the covalent addition of zidovudine. SANS measurements were performed using the D11 instrument at the Institut Laue – Langevin (ILL), Grenoble, France. A full outline of methods and parameters are provided in Section S.5 of the Supporting Information. A summary of the fitting parameters is shown in Table S4. The data and the fits are shown in Figure 3c and d.

### 4.5 Biostability

The *in vitro* biostability of peptoid-L-peptide (*N*Phe)_4_GGGGKY(p)-OH and peptoid-D-peptide

(*N*Phe)_4_GGGGky(p)-OH was tested using the broad-spectrum protease proteinase K at several time points for up to 28 days as outlined fully within the Supporting Information (S.6).

### 4.6 Cell Cytotoxicity

Peptoid-peptide cell cytotoxicity studies were performed using the International Standard (ISO) murine fibroblast subcutaneous connective tissue NCTC 929 (ATCC CCL 1) cell line.^108,116^ Cell cytotoxicity was assessed using three separate assays, outlined fully in Section S.7 of the Supporting Information: (i) 3-(4,5-dimethylthiazol-2-yl)-5-(3-carboxymethoxyphenyl)-2-(4-sulfophenyl)-2H-tetrazolium (MTS) colorimetric assay, (ii) lactate dehydrogenase (LDH) release and (iii) a Live/Dead assay. The cytotoxicity of each peptoid-peptide was tested after 6 (LDH, MTS), 24 (Live/Dead, MTS) and 72 h (MTS) of exposure.

### 4.7 *In Vitro* Drug Release

Zidovudine release from peptoid-L-peptide (*N*Phe)_4_GGGGKY(p)-OH and peptoid-D-peptide (*N*Phe)_4_GGGGky(p)-OH was tested over several time points up to 28 days (1, 2, 4, and 8 h; 1, 2, and 3 days and then weekly intervals of 7, 14, 21, and 28 days) in PBS (pH 7.4, 37 °C). Both covalently attached ((*N*Phe)_4_GGGGK(AZT)Y(p)-OH and (*N*Phe)_4_GGGGk(AZT)y(p)-OH) and physically mixed/encapsulated zidovudine (AZT) were tested as outlined in S.8 (Supporting Information).

### 4.8 *In Vivo* Plasma Drug Concentration Studies

The plasma concentration of zidovudine in Sprague–Dawley rats was evaluated for 35 days after subcutaneous administration of zidovudine covalently attached to phosphorylated peptoid-D-peptide (*N*Phe)_4_GGGGk(AZT)y(p)-OH. The study was approved by Queen’s University Belfast’s Ethics Committee (HM_2022_08) under the UK Home Office Project License (PPL2903). Female Sprague–Dawley rats (*n* = 18, aged 8–10 weeks, mean weight = 220 g) were purchased from Envigo and acclimatized for 1 week prior to experimentation. The rats were separated into three groups (*n* = 6 for each group), namely, two zidovudine groups (intravenous zidovudine control, subcutaneous (*N*Phe)_4_GGGGk(AZT)y(p)-OH) and a third untreated healthy control group. The methods employed and sample size calculation are outlined in section S.9 (Supporting Information).

### 4.9 Statistical Analysis

All the statistical analyzes were performed using Microsoft Excel 2021 and GraphPad Prism 10.1.2. Standard deviations (SDs) were obtained at each experimental concentration tested based on three replicates for biostability and *in vitro* drug release, six replicates for *in vivo* drug plasma concentrations and nine replicates for quantitative cell cytotoxicity assays. The Kruskal–Wallis test was used when the data were shown to be non-normally distributed according to the Kolmogorov and Smirnov test. Biostability was compared using a Kruskal–Wallis test with Dunn’s posthoc test to identify individual differences in the biostability compared to nontreated (100%) peptoid-peptide-only controls. Cell cytotoxicity (MTS and LDH assays) and oscillatory rheology (frequency sweeps: impact of L or D enantiomer and drug attachment on storage modulus (*G*′), three replicates) were also compared using a Kruskal–Wallis test with Dunn’s posthoc test. Peptoid-peptides were compared to negative controls (media only). The Kruskal–Wallis test was also employed for *in vitro* drug release assays to compare the release of physically encapsulated and covalently conjugated zidovudine release. A Dunn’s posthoc test identified individual differences in the data (within the same or across different time points). A probability of *p* ≤ 0.05 denoted significance in all cases. KinetDS 3.0 (SourceForge Media, La Jolla, CA, USA) was utilized to model *in vitro* drug release kinetics.^117^ PKSolver 2.0 was used to assess pharmacokinetic parameters with a noncompartmental model used to analyze plasma zidovudine concentration data obtained from the two zidovudine formulations, intravenous bolus injection and subcutaneous (*N*Phe)_4_GGGGk(AZT)y(p)-OH injection.^118^

## Data Availability

Data will be made available on request.

## Supporting Information Available

Materials and apparatus, synthesis methods and analysis, peptoid-peptide chemical structures, ^1^H and ^31^P NMRs, mass spectra, HPLC traces, formulation method, vial inversion assay results, rheology frequency, strain and time sweeps, SANS parameters, biostability method, cell cytotoxicity methods and data, *in vitro* drug release methods and modeling, *in vivo* drug release, calibration curves and PK parameters (PDF)

## References

[ref1] Mondal S.; Das S.; Nandi A. K. A review on recent advances in polymer and peptide hydrogels. Soft Matter 2020, 16 (6), 1404–1454. 10.1039/C9SM02127B. 31984400

[ref2] Du X.; Zhou J.; Shi J.; Xu B. Supramolecular hydrogelators and hydrogels: from soft matter to molecular biomaterials. Chem. Rev. 2015, 115 (24), 13165–13307. 10.1021/acs.chemrev.5b00299. 26646318 PMC4936198

[ref3] Cross E. R.; Coulter S. M.; Pentlavalli S.; Laverty G. Unravelling the antimicrobial activity of peptide hydrogel systems: Current and future perspectives. Soft Matter 2021, 17 (35), 8001–8021. 10.1039/D1SM00839K. 34525154 PMC8442837

[ref4] Gentilucci L.; Tolomelli A.; Squassabia F. Peptides and peptidomimetics in medicine, surgery and biotechnology. Curr. Med. Chem. 2006, 13 (20), 2449–2466. 10.2174/092986706777935041. 16918365

[ref5] Gentilucci L. S. New trends in the development of opioid peptide analogues as advanced remedies for pain relief. Current Topics in Medicinal Chemistry 2004, 4 (1), 19–38. 10.2174/1568026043451663. 14754374

[ref6] Melchionna M.; Styan K. E.; Marchesan S. The unexpected advantages of using D-amino acids for peptide self-assembly into nanostructured hydrogels for medicine. Curr. Top. Med. Chem. 2016, 16 (18), 2009–2018. 10.2174/1568026616999160212120302. 26876522 PMC5374841

[ref7] Li J.; Kuang Y.; Gao Y.; Du X.; Shi J.; Xu B. D-amino acids boost the selectivity and confer supramolecular hydrogels of a nonsteroidal anti-inflammatory drug (NSAID). J. Am. Chem. Soc. 2013, 135 (2), 542–545. 10.1021/ja310019x. 23136972 PMC3547157

[ref8] Panda J. J.; Mishra A.; Basu A.; Chauhan V. S. Stimuli responsive self-assembled hydrogel of a low molecular weight free dipeptide with potential for tunable drug delivery. Biomacromolecules 2008, 9 (8), 2244–2250. 10.1021/bm800404z. 18624454

[ref9] Castelletto V.; Cheng G.; Hamley I. W. Amyloid peptides incorporating a core sequence from the amyloid beta peptide and gamma amino acids: Relating bioactivity to self-assembly. Chem. Commun. 2011, 47 (46), 12470–12472. 10.1039/c1cc15493a. 22042055

[ref10] Bicker K. L.; Cobb S. L. Recent advances in the development of anti-infective peptoids. Chem. Commun. 2020, 56 (76), 11158–11168. 10.1039/D0CC04704J. 32870199

[ref11] Rick F. G.; Block N. L.; Schally A. V. An update on the use of degarelix in the treatment of advanced hormone-dependent prostate cancer. Onco Targets Ther 2013, 6, 391–402. 10.2147/OTT.S32426. 23620672 PMC3633549

[ref12] Wegner U.; Matthes F.; von Wirén N.; Lemke I.; Bode R.; Vorbrodt H. M.; Rauter M.; Kunze G. Enhancing a Sphaerobacter thermophilus ω-transaminase for kinetic resolution of β- and γ-amino acids. AMB Express 2023, 13 (1), 117 10.1186/s13568-023-01623-x. 37864072 PMC10589169

[ref13] Simon R. J.; Kania R. S.; Zuckermann R. N.; Huebner V. D.; Jewell D. A.; Banville S.; Ng S.; Wang L.; Rosenberg S.; Marlowe C. K. Peptoids: A modular approach to drug discovery. Proc. Natl. Acad. Sci. U. S. A. 1992, 89 (20), 9367–9371. 10.1073/pnas.89.20.9367. 1409642 PMC50132

[ref14] Cai B.; Li Z.; Chen C.-L. Programming amphiphilic peptoid oligomers for hierarchical assembly and inorganic crystallization. Acc. Chem. Res. 2021, 54 (1), 81–91. 10.1021/acs.accounts.0c00533. 33136361

[ref15] Zuckermann R. N. Peptoid origins. Peptide Science 2011, 96 (5), 545–555. 10.1002/bip.21573. 21184486

[ref16] Clapperton A. M.; Babi J.; Tran H. A Field Guide to Optimizing Peptoid Synthesis. ACS Polymers Au 2022, 2 (6), 417–429. 10.1021/acspolymersau.2c00036. 36536890 PMC9756346

[ref17] Saini A.; Verma G. Peptoids: tomorrow’s therapeutics. In Nanostructures for Novel Therapy, 1st ed.; Elsevier, 2017; pp 251–280.

[ref18] Patch J. A.; Kirshenbaum K.; Seurynck S. L.; Zuckermann R. N.; Barron A. E. Versatile oligo (N-substituted) glycines: The many roles of peptoids in drug discovery; Wiley Online Library, 2004.

[ref19] Li Z.; Cai B.; Yang W.; Chen C.-L. Hierarchical nanomaterials assembled from peptoids and other sequence-defined synthetic polymers. Chem. Rev. 2021, 121 (22), 14031–14087. 10.1021/acs.chemrev.1c00024. 34342989

[ref20] Jin H.; Jiao F.; Daily M. D.; Chen Y.; Yan F.; Ding Y.-H.; Zhang X.; Robertson E. J.; Baer M. D.; Chen C. L. Highly stable and self-repairing membrane-mimetic 2D nanomaterials assembled from lipid-like peptoids. Nat. Commun. 2016, 7 (1), 12252 10.1038/ncomms12252. 27402325 PMC4945955

[ref21] Robertson E. J.; Olivier G. K.; Qian M.; Proulx C.; Zuckermann R. N.; Richmond G. L. Assembly and molecular order of two-dimensional peptoid nanosheets through the oil-water interface. Proc. Natl. Acad. Sci. U. S. A. 2014, 111 (37), 13284–13289. 10.1073/pnas.1414843111. 25197049 PMC4169914

[ref22] Ma X.; Zhang S.; Jiao F.; Newcomb C. J.; Zhang Y.; Prakash A.; Liao Z.; Baer M. D.; Mundy C. J.; Pfaendtner J.; et al. Tuning crystallization pathways through sequence engineering of biomimetic polymers. Nat. Mater. 2017, 16 (7), 767–774. 10.1038/nmat4891. 28414316

[ref23] Chen C.-L.; Zuckermann R. N.; DeYoreo J. J. Surface-directed assembly of sequence-defined synthetic polymers into networks of hexagonally patterned nanoribbons with controlled functionalities. ACS Nano 2016, 10 (5), 5314–5320. 10.1021/acsnano.6b01333. 27136277

[ref24] Jin H.; Ding Y.-H.; Wang M.; Song Y.; Liao Z.; Newcomb C. J.; Wu X.; Tang X.-Q.; Li Z.; Lin Y.; et al. Designable and dynamic single-walled stiff nanotubes assembled from sequence-defined peptoids. Nat. Commun. 2018, 9 (1), 270 10.1038/s41467-017-02059-1. 29348551 PMC5773689

[ref25] Sun J.; Jiang X.; Lund R.; Downing K. H.; Balsara N. P.; Zuckermann R. N. Self-assembly of crystalline nanotubes from monodisperse amphiphilic diblock copolypeptoid tiles. Proc. Natl. Acad. Sci. U. S. A. 2016, 113 (15), 3954–3959. 10.1073/pnas.1517169113. 27035944 PMC4839393

[ref26] Merrill N. A.; Yan F.; Jin H.; Mu P.; Chen C.-L.; Knecht M. R. Tunable assembly of biomimetic peptoids as templates to control nanostructure catalytic activity. Nanoscale 2018, 10 (26), 12445–12452. 10.1039/C8NR03852J. 29926884

[ref27] Jin H.; Jian T.; Ding Y. H.; Chen Y.; Mu P.; Wang L.; Chen C. L. Solid-phase synthesis of three-armed star-shaped peptoids and their hierarchical self-assembly. Biopolymers 2019, 110 (4), e23258 10.1002/bip.23258. 30676654

[ref28] Armand P.; Kirshenbaum K.; Falicov A.; Dunbrack R. L. Jr; Dill K. A.; Zuckermann R. N.; Cohen F. E. Chiral *N*-substituted glycines can form stable helical conformations. Folding and Design 1997, 2 (6), 369–375. 10.1016/S1359-0278(97)00051-5. 9427011

[ref29] Nam K. T.; Shelby S. A.; Choi P. H.; Marciel A. B.; Chen R.; Tan L.; Chu T. K.; Mesch R. A.; Lee B.-C.; Connolly M. D.; et al. Free-floating ultrathin two-dimensional crystals from sequence-specific peptoid polymers. Nat. Mater. 2010, 9 (5), 454–454. 10.1038/nmat2742. 20383129

[ref30] Kudirka R.; Tran H.; Sanii B.; Nam K. T.; Choi P. H.; Venkateswaran N.; Chen R.; Whitelam S.; Zuckermann R. N. Folding of a single-chain, information-rich polypeptoid sequence into a highly ordered nanosheet. Peptide Science 2011, 96 (5), 586–595. 10.1002/bip.21590. 22180906

[ref31] Wu Z.; Tan M.; Chen X.; Yang Z.; Wang L. Molecular hydrogelators of peptoid-peptide conjugates with superior stability against enzyme digestion. Nanoscale 2012, 4 (12), 3644–3646. 10.1039/c2nr30408b. 22581113

[ref32] Gao Y.; Kuang Y.; Guo Z. F.; Guo Z.; Krauss I. J.; Xu B. Enzyme-instructed molecular self-assembly confers nanofibers and a supramolecular hydrogel of taxol derivative. J. Am. Chem. Soc. 2009, 131 (38), 13576–13577. 10.1021/ja904411z. 19731909

[ref33] British Pharmacopoeial Commission. British Pharmacopoeia, Monograph: Zidovudine. The Stationery Office, 2024. https://www-pharmacopoeia-com.queens.ezp1.qub.ac.uk/bp-2024/monographs/zidovudine.html?date=2024-07-01&text=zidovudine (accessed 13th December 2023).

[ref34] World Health Organization. WHO HIV Fact Sheets. 2021. https://www.who.int/news-room/fact-sheets/detail/hiv-aids (accessed 15th September 2022).

[ref35] UNAIDS. Fast-Track Ending the AIDS Epidemic by 2030. 2014; Vol. 2019. https://www.unaids.org/sites/default/files/media_asset/JC2686_WAD2014report_en.pdf.

[ref36] World Health Organization. Data on the HIV response. https://www.who.int/data/gho/data/themes/hiv-aids/data-on-the-hiv-aids-response (accessed 19th September 2022).

[ref37] Menéndez-Arias L.; Delgado R. Update and latest advances in antiretroviral therapy. Trends Pharmacol. Sci. 2022, 43 (1), 16–29. 10.1016/j.tips.2021.10.004. 34742581

[ref38] Hogg R. S.; Heath K.; Bangsberg D.; Yip B.; Press N.; O’Shaughnessy M. V.; Montaner J. S. G. Intermittent use of triple-combination therapy is predictive of mortality at baseline and after 1 year of follow-up. Aids 2002, 16 (7), 1051–1058. 10.1097/00002030-200205030-00012. 11953472

[ref39] Prieto P.; Podzamczer D. Switching strategies in the recent era of antiretroviral therapy. Expert Review of Clinical Pharmacology 2019, 12 (3), 235–247. 10.1080/17512433.2019.1575728. 30691315

[ref40] Kerrigan D.; Mantsios A.; Gorgolas M.; Montes M. L.; Pulido F.; Brinson C.; deVente J.; Richmond G. J.; Beckham S. W.; Hammond P.; et al. Experiences with long acting injectable ART: A qualitative study among PLHIV participating in a Phase II study of cabotegravir + rilpivirine (LATTE-2) in the United States and Spain. PloS one 2018, 13 (1), e0190487 10.1371/journal.pone.0190487. 29304154 PMC5755771

[ref41] Graham S. M.; Barthold D.; Hauber B.; Brah A. T.; Saldarriaga E.; Collier A. C.; Ho R. J. Y.; Marconi V. C.; Simoni J. M. U.S. patient preferences for long-acting HIV treatment: a discrete choice experiment. J. Int. AIDS Soc. 2023, 26 (S2), e26099 10.1002/jia2.26099. 37439051 PMC10338996

[ref42] Orkin C.; Oka S.; Philibert P.; Brinson C.; Bassa A.; Gusev D.; Degen O.; García J. G.; Morell E. B.; Tan D. H. Long-acting cabotegravir plus rilpivirine for treatment in adults with HIV-1 infection: 96-week results of the randomised, open-label, phase 3 FLAIR study. Lancet HIV 2021, 8 (4), e185 10.1016/S2352-3018(20)30340-4. 33794181

[ref43] Landovitz R. J.; Hanscom B. S.; Clement M. E.; Tran H. V.; Kallas E. G.; Magnus M.; Sued O.; Sanchez J.; Scott H.; Eron J. J.; et al. Efficacy and safety of long-acting cabotegravir compared with daily oral tenofovir disoproxil fumarate plus emtricitabine to prevent HIV infection in cisgender men and transgender women who have sex with men 1 year after study unblinding: a secondary analysis of the phase 2b and 3 HPTN 083 randomised controlled trial. Lancet HIV 2023, 10 (12), e767 10.1016/S2352-3018(23)00261-8. 37952550 PMC11375758

[ref44] Flexner C.; Owen A.; Siccardi M.; Swindells S. Long-acting drugs and formulations for the treatment and prevention of HIV infection. Int. J. Antimicrob. Agents 2021, 57 (1), 106220 10.1016/j.ijantimicag.2020.106220. 33166693 PMC7790856

[ref45] Shi Y.; Lu A.; Wang X.; Belhadj Z.; Wang J.; Zhang Q. A review of existing strategies for designing long-acting parenteral formulations: Focus on underlying mechanisms, and future perspectives. Acta Pharm. Sin B 2021, 11 (8), 2396–2415. 10.1016/j.apsb.2021.05.002. 34522592 PMC8424287

[ref46] Wilkinson J.; Ajulo D.; Tamburrini V.; Gall G. L.; Kimpe K.; Holm R.; Belton P.; Qi S. Lipid based intramuscular long-acting injectables: Current state of the art. European Journal of Pharmaceutical Sciences 2022, 178, 106253 10.1016/j.ejps.2022.106253. 35793750

[ref47] Palomba S.; Falbo A.; Di Cello A.; Materazzo C.; Zullo F. Nexplanon: The new implant for long-term contraception. A comprehensive descriptive review. Gynecological Endocrinology 2012, 28 (9), 710–721. 10.3109/09513590.2011.652247. 22339096

[ref48] Verma S.; Kumar S.; Gokhale R.; Burgess D. J. Physical stability of nanosuspensions: investigation of the role of stabilizers on Ostwald ripening. Int. J. Pharm. 2011, 406 (1–2), 145–152. 10.1016/j.ijpharm.2010.12.027. 21185926

[ref49] Gao Y.; Li Z.; Sun M.; Li H.; Guo C.; Cui J.; Li A.; Cao F.; Xi Y.; Lou H.; et al. Preparation, characterization, pharmacokinetics, and tissue distribution of curcumin nanosuspension with TPGS as stabilizer. Drug Dev. Ind. Pharm. 2010, 36 (10), 1225–1234. 10.3109/03639041003695139. 20545506

[ref50] Madras G.; McCoy B. J. Temperature effects during Ostwald ripening. J. Chem. Phys. 2003, 119 (3), 1683–1693. 10.1063/1.1578617.

[ref51] Zuckermann R. N.; Kerr J. M.; Kent S. B. H.; Moos W. H. Efficient method for the preparation of peptoids [oligo (*N*-substituted glycines)] by submonomer solid-phase synthesis. J. Am. Chem. Soc. 1992, 114 (26), 10646–10647. 10.1021/ja00052a076.

[ref52] Proulx C.; Yoo S.; Connolly M. D.; Zuckermann R. N. Accelerated submonomer solid-phase synthesis of peptoids incorporating multiple substituted *N*-aryl glycine monomers. Journal of Organic Chemistry 2015, 80 (21), 10490–10497. 10.1021/acs.joc.5b01449. 26280152

[ref53] Robertson E. J.; Battigelli A.; Proulx C.; Mannige R. V.; Haxton T. K.; Yun L.; Whitelam S.; Zuckermann R. N. Design, synthesis, assembly, and engineering of peptoid nanosheets. Acc. Chem. Res. 2016, 49 (3), 379–389. 10.1021/acs.accounts.5b00439. 26741294

[ref54] Yu A. C.; Chen H.; Chan D.; Agmon G.; Stapleton L. M.; Sevit A. M.; Tibbitt M. W.; Acosta J. D.; Zhang T.; Franzia P. W.; et al. Scalable manufacturing of biomimetic moldable hydrogels for industrial applications. Proc. Natl. Acad. Sci. U. S. A. 2016, 113 (50), 14255–14260. 10.1073/pnas.1618156113. 27911849 PMC5167152

[ref55] Sebastian S.; Yadav E.; Bhardwaj P.; Maruthi M.; Kumar D.; Gupta M. K. Facile one-pot multicomponent synthesis of peptoid based gelators as novel scaffolds for drug incorporation and pH-sensitive release. J. Mater. Chem. B 2023, 11 (41), 9975–9986. 10.1039/D3TB01527K. 37823277

[ref56] Yang Z.; Liang G.; Guo Z.; Guo Z.; Xu B. Intracellular hydrogelation of small molecules inhibits bacterial growth. Angew. Chem. 2007, 46 (43), 8216–8219. 10.1002/anie.200701697. 17705321

[ref57] Yang Z.; Liang G.; Ma M.; Gao Y.; Xu B. *In vitro* and *in vivo* enzymatic formation of supramolecular hydrogels based on self-assembled nanofibers of a beta-amino acid derivative. Small 2007, 3 (4), 558–562. 10.1002/smll.200700015. 17323399

[ref58] Sedighi M.; Shrestha N.; Mahmoudi Z.; Khademi Z.; Ghasempour A.; Dehghan H.; Talebi S. F.; Toolabi M.; Préat V.; Chen B. Multifunctional self-assembled peptide hydrogels for biomedical applications. Polymers 2023, 15 (5), 1160 10.3390/polym15051160. 36904404 PMC10007692

[ref59] Hartgerink J. D.; Beniash E.; Stupp S. I. Self-assembly and mineralization of peptide-amphiphile nanofibers. Science 2001, 294 (5547), 1684–1688. 10.1126/science.1063187. 11721046

[ref60] Liu L.; Xu K.; Wang H.; Tan P. K.; Fan W.; Venkatraman S. S.; Li L.; Yang Y. Y. Self-assembled cationic peptide nanoparticles as an efficient antimicrobial agent. Nat. Nanotechnol. 2009, 4 (7), 457–463. 10.1038/nnano.2009.153. 19581900

[ref61] Taraballi F.; Natalello A.; Campione M.; Villa O.; Doglia S. M.; Paleari A.; Gelain F. Glycine-spacers influence functional motifs exposure and self-assembling propensity of functionalized substrates tailored for neural stem cell cultures. Front Neuroeng 2010, 3, 1–1. 10.3389/neuro.16.001.2010. 20162033 PMC2821182

[ref62] Wang H.; Yang C.; Tan M.; Wang L.; Kong D.; Yang Z. A structure-gelation ability study in a short peptide-based ‘Super Hydrogelator’ system. Soft Matter 2011, 7 (8), 3897–3905. 10.1039/c0sm01405b.

[ref63] Coulter S. M.; Pentlavalli S.; Vora L. K.; An Y.; Cross E. R.; Peng K.; McAulay K.; Schweins R.; Donnelly R. F.; McCarthy H. O.; Laverty G. Enzyme-triggered L-α/D-peptide hydrogels as a long-acting injectable platform for systemic delivery of HIV/AIDS Drugs. Adv. Healthcare Mater. 2023, 12 (18), 2203198 10.1002/adhm.202203198. PMC11469249 36880399

[ref64] Li J.; Gao Y.; Kuang Y.; Shi J.; Du X.; Zhou J.; Wang H.; Yang Z.; Xu B. Dephosphorylation of d-Peptide Derivatives to form biofunctional, supramolecular nanofibers/hydrogels and their potential applications for intracellular imaging and intratumoral chemotherapy. J. Am. Chem. Soc. 2013, 135 (26), 9907–9914. 10.1021/ja404215g. 23742714 PMC3730259

[ref65] Yang Z.; Gu H.; Fu D.; Gao P.; Lam J. K.; Xu B. enzymatic formation of supramolecular hydrogels. Adv. Mater. 2004, 16 (16), 1440–1444. 10.1002/adma.200400340.

[ref66] Wang H.; Feng Z.; Wu D.; Fritzsching K. J.; Rigney M.; Zhou J.; Jiang Y.; Schmidt-Rohr K.; Xu B. Enzyme-regulated supramolecular assemblies of cholesterol conjugates against drug-resistant ovarian cancer cells. J. Am. Chem. Soc. 2016, 138 (34), 10758–10761. 10.1021/jacs.6b06075. 27529637 PMC5010010

[ref67] Rafferty J.; Nagaraj H.; McCloskey A. P.; Huwaitat R.; Porter S.; Albadr A.; Laverty G. Peptide therapeutics and the pharmaceutical industry: Barriers encountered translating from the laboratory to patients. Curr. Med. Chem. 2016, 23 (37), 4231–4259. 10.2174/0929867323666160909155222. 27633684

[ref68] Ulijn R. V. Peptide-based materials via molecular self-assembly. In Self-Assembled Peptide Nanostructures: Advances and Applications in Nanobiotechnology; Castillo J. S. L, Svendsen W. E., Ed.; 2013.

[ref69] Swanson H. W. A.; Lau K. H. A.; Tuttle T. Minimal peptoid dynamics inform self-assembly propensity. J. Phys. Chem. B 2023, 127 (49), 10601–10614. 10.1021/acs.jpcb.3c03725. 38038956 PMC10726364

[ref70] Frederix P. W.; Scott G. G.; Abul-Haija Y. M.; Kalafatovic D.; Pappas C. G.; Javid N.; Hunt N. T.; Ulijn R. V.; Tuttle T. Exploring the sequence space for (tri-)peptide self-assembly to design and discover new hydrogels. Nat. Chem. 2015, 7 (1), 30–37. 10.1038/nchem.2122. 25515887

[ref71] Porter S. L.; Coulter S. M.; Pentlavalli S.; Thompson T. P.; Laverty G. Self-assembling diphenylalanine peptide nanotubes selectively eradicate bacterial biofilm infection. Acta Biomaterialia 2018, 77, 96–105. 10.1016/j.actbio.2018.07.033. 30031161

[ref72] Cai X.; Xu W.; Ren C.; Zhang L.; Zhang C.; Liu J.; Yang C. Recent progress in quantitative analysis of self-assembled peptides. Exploration 2024, 20230064 10.1002/EXP.20230064. PMC11335468 39175887

[ref73] Shi J.; Du X.; Yuan D.; Zhou J.; Zhou N.; Huang Y.; Xu B. D-amino acids modulate the cellular response of enzymatic-instructed supramolecular nanofibers of small peptides. Biomacromolecules 2014, 15 (10), 3559–3568. 10.1021/bm5010355. 25230147 PMC4195520

[ref74] Feng Z.; Wang H.; Chen X.; Xu B. Self-assembling ability determines the activity of enzyme-instructed self-assembly for inhibiting cancer cells. J. Am. Chem. Soc. 2017, 139 (43), 15377–15384. 10.1021/jacs.7b07147. 28990765 PMC5669277

[ref75] Kalafatovic D.; Nobis M.; Son J.; Anderson K. I.; Ulijn R. V. MMP-9 triggered self-assembly of doxorubicin nanofiber depots halts tumor growth. Biomaterials 2016, 98, 192–202. 10.1016/j.biomaterials.2016.04.039. 27192421

[ref76] Ren C.; Chu L.; Huang F.; Yang L.; Fan H.; Liu J.; Yang C. A novel H_2_O_2_ responsive supramolecular hydrogel for controllable drug release. RSC Adv. 2017, 7 (3), 1313–1317. 10.1039/C6RA26536G.

[ref77] Adams D. J. Personal perspective on understanding low molecular weight gels. J. Am. Chem. Soc. 2022, 144 (25), 11047–11053. 10.1021/jacs.2c02096. 35713375 PMC9248009

[ref78] Draper E. R.; Adams D. J. Controlling supramolecular gels. Nat. Mater. 2024, 23 (1), 13–15. 10.1038/s41563-023-01765-0. 38172550

[ref79] Burgess K. A.; Frati C.; Meade K.; Gao J.; Castillo Diaz L.; Madeddu D.; Graiani G.; Cavalli S.; Miller A. F.; Oceandy D.; et al. Functionalised peptide hydrogel for the delivery of cardiac progenitor cells. Materials Science and Engineering: C 2021, 119, 111539 10.1016/j.msec.2020.111539. 33321610

[ref80] Ling Y.; Gao Y.; Shu C.; Zhou Y.; Zhong W.; Xu B. Using a peptide segment to covalently conjugate doxorubicin and taxol for the study of drug combination effect. RSC Adv. 2015, 5, 101475–101479. 10.1039/C5RA14156G.

[ref81] Ashworth J. C.; Thompson J. L.; James J. R.; Slater C. E.; Pijuan-Galitó S.; Lis-Slimak K.; Holley R. J.; Meade K. A.; Thompson A.; Arkill K. P.; et al. Peptide gels of fully-defined composition and mechanics for probing cell-cell and cell-matrix interactions *in vitro*. Matrix Biology 2020, 85–86, 15–33. 10.1016/j.matbio.2019.06.009. PMC7610915 31295578

[ref82] Nagy-Smith K.; Beltramo P. J.; Moore E.; Tycko R.; Furst E. M.; Schneider J. P. Molecular, local, and network-level basis for the enhanced stiffness of hydrogel networks formed from coassembled racemic peptides: Predictions from Pauling and Corey. ACS Central Science 2017, 3 (6), 586–597. 10.1021/acscentsci.7b00115. 28691070 PMC5492410

[ref83] Chen Z.; Xing L.; Fan Q.; Cheetham A. G.; Lin R.; Holt B.; Chen L.; Xiao Y.; Cui H. Drug-bearing supramolecular filament hydrogels as anti-inflammatory agents. Theranostics 2017, 7 (7), 2003–2003. 10.7150/thno.19404. 28656057 PMC5485419

[ref84] Suh M. S.; Kastellorizios M.; Tipnis N.; Zou Y.; Wang Y.; Choi S.; Burgess D. J. Effect of implant formation on drug release kinetics of *in situ* forming implants. Int. J. Pharm. 2021, 592, 120105 10.1016/j.ijpharm.2020.120105. 33232755

[ref85] Thornton K.; Abul-Haija Y. M.; Hodson N.; Ulijn R. V. Mechanistic insights into phosphatase triggered self-assembly including enhancement of biocatalytic conversion rate. Soft Matter 2013, 9 (39), 9430–9439. 10.1039/c3sm51177d.

[ref86] Hu X.; Liao M.; Gong H.; Zhang L.; Cox H.; Waigh T. A.; Lu J. R. Recent advances in short peptide self-assembly: from rational design to novel applications. Curr. Opin. Colloid Interface Sci. 2020, 45, 1–13. 10.1016/j.cocis.2019.08.003.

[ref87] Laverty G.; McCloskey A. P.; Gilmore B. F.; Jones D. S.; Zhou J.; Xu B. Ultrashort cationic naphthalene-derived self-assembled peptides as antimicrobial nanomaterials. Biomacromolecules 2014, 15 (9), 3429–3439. 10.1021/bm500981y. 25068387

[ref88] McCloskey A. P.; Gilmore S. M.; Zhou J.; Draper E. R.; Porter S.; Gilmore B. F.; Xu B.; Laverty G. Self-assembling ultrashort NSAID-peptide nanosponges: Multifunctional antimicrobial and anti-inflammatory materials. See DOI: 10.1039/c6ra20282a. RSC Adv. 2016, 6 (115), 114738–114749. 10.1039/C6RA20282A.

[ref89] Draper E. R.; Dietrich B.; McAulay K.; Brasnett C.; Abdizadeh H.; Patmanidis I.; Marrink S. J.; Su H.; Cui H.; Schweins R.; et al. Using small-angle scattering and contrast matching to understand molecular packing in low molecular weight gels. Matter 2020, 2 (3), 764–778. 10.1016/j.matt.2019.12.028.

[ref90] McDowall D.; Adams D. J.; Seddon A. M. Using small angle scattering to understand low molecular weight gels. Soft Matter 2022, 18 (8), 1577–1590. 10.1039/D1SM01707A. 35147629

[ref91] Frackenpohl J.; Arvidsson P. I.; Schreiber J. V.; Seebach D. The outstanding biological stability of β-and γ-peptides toward proteolytic enzymes: an in vitro investigation with fifteen peptidases. ChemBioChem. 2001, 2 (6), 445–455. 10.1002/1439-7633(20010601)2:6<445::AID-CBIC445>3.0.CO;2-R. 11828476

[ref92] Bomb K.; Zhang Q.; Ford E. M.; Fromen C. A.; Kloxin A. M. Systematic D-amino acid substitutions to control peptide and hydrogel degradation in cellular microenvironments. ACS Macro Lett. 2023, 12, 725–732. 10.1021/acsmacrolett.3c00144. 37195203 PMC10560456

[ref93] Liang G.; Yang Z.; Zhang R.; Li L.; Fan Y.; Kuang Y.; Gao Y.; Wang T.; Lu W. W.; Xu B. Supramolecular hydrogel of a D-amino acid dipeptide for controlled drug release *in vivo*. Langmuir 2009, 25 (15), 8419–8422. 10.1021/la804271d. 20050040

[ref94] Thakur R. R. S.; McMillan H. L.; Jones D. S. Solvent induced phase inversion-based in situ forming controlled release drug delivery implants. J. Controlled Release 2014, 176, 8–23. 10.1016/j.jconrel.2013.12.020. 24374003

[ref95] Li Q.; Li X.; Zhao C. Strategies to obtain encapsulation and controlled release of small hydrophilic molecules. Front. Bioeng. Biotechnol. 2020, 8, 437 10.3389/fbioe.2020.00437. 32478055 PMC7237580

[ref96] Stojkov G.; Niyazov Z.; Picchioni F.; Bose R. K. Relationship between structure and rheology of hydrogels for various applications. Gels 2021, 7 (4), 255 10.3390/gels7040255. 34940315 PMC8700820

[ref97] Caccavo D. An overview on the mathematical modeling of hydrogels’ behavior for drug delivery systems. Int. J. Pharm. 2019, 560, 175–190. 10.1016/j.ijpharm.2019.01.076. 30763681

[ref98] Qureshi D.; Nayak S. K.; Maji S.; Anis A.; Kim D.; Pal K. Environment sensitive hydrogels for drug delivery applications. Eur. Polym. J. 2019, 120, 109220 10.1016/j.eurpolymj.2019.109220.

[ref99] Vigata M.; Meinert C.; Hutmacher D. W.; Bock N. Hydrogels as drug delivery systems: A review of current characterization and evaluation techniques. Pharmaceutics 2020, 12 (12), 1188 10.3390/pharmaceutics12121188. 33297493 PMC7762425

[ref100] Lakshani N.; Wijerathne H. S.; Sandaruwan C.; Kottegoda N.; Karunarathne V. Release kinetic models and release mechanisms of controlled-release and slow-release fertilizers. ACS Agricultural Science & Technology 2023, 3 (11), 939–956. 10.1021/acsagscitech.3c00152.

[ref101] Bubpamala T.; Viravaidya-Pasuwat K.; Pholpabu P. Injectable poly(ethylene glycol) hydrogels cross-linked by metal-phenolic complex and albumin for controlled drug release. ACS Omega 2020, 5 (31), 19437–19445. 10.1021/acsomega.0c01393. 32803037 PMC7424574

[ref102] Rezaeian Shiadeh S. N.; Hadizadeh F.; Khodaverdi E.; Gorji Valokola M.; Rakhshani S.; Kamali H.; Nokhodchi A. Injectable *in-situ* forming depot based on PLGA and PLGA-PEG-PLGA for sustained-release of risperidone: *In vitro* evaluation and pharmacokinetics in rabbits. Pharmaceutics 2023, 15 (4), 1229 10.3390/pharmaceutics15041229. 37111714 PMC10143068

[ref103] Mikac U.; Sepe A.; Gradišek A.; Kristl J.; Apih T. Dynamics of water and xanthan chains in hydrogels studied by NMR relaxometry and their influence on drug release. Int. J. Pharm. 2019, 563, 373–383. 10.1016/j.ijpharm.2019.04.014. 30965122

[ref104] Choe R.; Yun S. I. Fmoc-diphenylalanine-based hydrogels as a potential carrier for drug delivery. e-Polym. 2020, 20 (1), 458–468. 10.1515/epoly-2020-0050.

[ref105] Papadopoulou V.; Kosmidis K.; Vlachou M.; Macheras P. On the use of the Weibull function for the discernment of drug release mechanisms. Int. J. Pharm. 2006, 309 (1), 44–50. 10.1016/j.ijpharm.2005.10.044. 16376033

[ref106] Yang Z.; Liang G.; Wang L.; Xu B. Using a kinase/phosphatase switch to regulate a supramolecular hydrogel and forming the supramolecular hydrogel *in vivo*. J. Am. Chem. Soc. 2006, 128 (9), 3038–3043. 10.1021/ja057412y. 16506785

[ref107] Niss F.; Rosenmai A. K.; Mandava G.; Örn S.; Oskarsson A.; Lundqvist J. Toxicity bioassays with concentrated cell culture media—a methodology to overcome the chemical loss by conventional preparation of water samples. Environmental Science and Pollution Research 2018, 25 (12), 12183–12188. 10.1007/s11356-018-1656-4. 29525858 PMC5940719

[ref108] International Organization for Standardization. E. N. 10993–5, Biological evaluation of medical devices-Part 5: Tests for in vitro cytotoxicity; International Organization for Standardization: Geneva, 2009.

[ref109] Bhana N.; Ormrod D.; Perry C. M.; Figgitt D. P. Zidovudine: A review of its use in the management of vertically-acquired pediatric HIV infection. Pediatric Drugs 2002, 4 (8), 515–553. 10.2165/00128072-200204080-00004. 12126455

[ref110] Young I. C.; Pallerla A.; Cottrell M. L.; Maturavongsadit P.; Prasher A.; Shrivastava R.; De la Cruz G.; Montgomery S. A.; Schauer A.; Sykes C.; et al. Long-acting injectable multipurpose prevention technology for prevention of HIV and unplanned pregnancy. J. Controlled Release 2023, 363, 606–620. 10.1016/j.jconrel.2023.10.006. PMC10841820 37797892

[ref111] Vora L. K.; Tekko I. A.; Zanutto F. V.; Sabri A.; Choy R. K. M.; Mistilis J.; Kwarteng P.; Jarrahian C.; McCarthy H. O.; Donnelly R. F. A bilayer microarray patch (MAP) for HIV pre-exposure prophylaxis: The role of MAP designs and formulation composition in enhancing long-acting drug delivery. Pharmaceutics 2024, 16 (1), 142 10.3390/pharmaceutics16010142. 38276512 PMC10819247

[ref112] Slama L.; Porcher R.; Linard F.; Chakvetadze C.; Cros A.; Carillon S.; Gallardo L.; Viard J.-P.; Molina J.-M. Injectable long acting antiretroviral for HIV treatment and prevention: Perspectives of potential users. BMC Infectious Diseases 2023, 23 (1), 98 10.1186/s12879-023-08071-9. 36803606 PMC9936705

[ref113] Nachega J. B.; Scarsi K. K.; Gandhi M.; Scott R. K.; Mofenson L. M.; Archary M.; Nachman S.; Decloedt E.; Geng E. H.; Wilson L.; et al. Long-acting antiretrovirals and HIV treatment adherence. Lancet HIV 2023, 10 (5), e332 10.1016/S2352-3018(23)00051-6. 37062293 PMC10734401

[ref114] Geddes L.; Themistou E.; Burrows J. F.; Buchanan F. J.; Carson L. Evaluation of the *in vitro* cytotoxicity and modulation of the inflammatory response by the bioresorbable polymers poly(D,L-lactide-co-glycolide) and poly(L-lactide-co-glycolide). Acta Biomaterialia 2021, 134, 261 10.1016/j.actbio.2021.07.049. 34329786

[ref115] Farage M. A.; Maibach H. I.; Andersen K. E.; Lachapelle J. M.; Kern P.; Ryan C.; Ely J.; Kanti A. Historical perspective on the use of visual grading scales in evaluating skin irritation and sensitization. Contact Dermatitis 2011, 65 (2), 65–75. 10.1111/j.1600-0536.2011.01912.x. 21668861

[ref116] Chen B.; Zhu D.; Li Q.; Wang C.; Cui J.; Zheng Z.; Wang X. Mechanically reinforced and injectable universal adhesive based on a PEI-PAA/Alg dual-network hydrogel designed by topological entanglement and catechol chemistry. ACS Appl. Mater. Interfaces 2023, 15 (51), 59826–59837. 10.1021/acsami.3c14743. 38098133

[ref117] Mendyk A. J. R.; Jachowicz R. Unified methodology of neural analysis in decision support systems built for pharmaceutical technology. Expert Syst. Appl. 2007, 32, 1124–1131. 10.1016/j.eswa.2006.02.019.

[ref118] Zhang Y.; Huo M.; Zhou J.; Xie S. PKSolver: An add-in program for pharmacokinetic and pharmacodynamic data analysis in Microsoft Excel. Computer Methods and Programs in Biomedicine 2010, 99 (3), 306–314. 10.1016/j.cmpb.2010.01.007. 20176408

