# Supplementary material for: In Situ Forming, Enzyme-Responsive Peptoid-Peptide Hydrogels: An Advanced Long-Acting Injectable Drug Delivery System
Source: J Am Chem Soc. 2024 Jun 26;146(31):21401–16. doi: 10.1021/jacs.4c03751 (PMC11311241; doi:10.1021/jacs.4c03751)
Supplement: Supplementary file 1 — ja4c03751_si_001.pdf [file ja4c03751_si_001.pdf]

Supporting Information

In situ forming, enzyme-responsive peptoid-peptide hydrogels: An advanced long-acting injectable drug delivery system.

Sophie M. Coulter,[a] Sreekanth Pentlavalli,[a] Yuming An,[a] Lalitkumar K. Vora,[a] Emily R. Cross,[a] Jessica V. Moore,[a] Han Sun,[a] Ralf Schweins,[b] Helen O. McCarthy,[a] Garry Laverty[a]*

[a]Biofunctional Nanomaterials Group, School of Pharmacy, Queen's University Belfast, Medical Biology Centre, 97 Lisburn Road, Belfast, Co. Antrim, BT9 7BL, N. Ireland.

[b]Large Scale Structures Group, Institut Laue – Langevin, 71 Avenue des Martyrs, CS 20156, Grenoble Cedex 9, 38042 France.

E-mail: garry.laverty@qub.ac.uk

S.1. Materials and apparatus

S.1.1. Synthesis: Sintered glass funnels and round bottomed flasks VWR (VWR, Lutterworth, Leicestershire, UK). Resins, including Wang resin (100/200 mesh particle size, 1.1 mmol/g loading) and Fmoc-Gly-Wang resin (100/200 mesh particle size, 0.4 – 0.8 mmol/g loading), *n*-butanol and Fmoc protected amino acids including L and D-Fmoc-Tyr-OH were purchased from Sigma Aldrich (Sigma Aldrich, Gillingham, Dorset, UK). Dimethylformamide (DMF), anhydrous DMF, 1-hydroxybenzotriazole hydrate (HOBt), 2-(1H-benzotriazol-1-yl)-1,1,3,3-tetramethyluronium hexafluorophosphate (HBTU), diisopropyl ethylamine (DIPEA), methanol, anhydrous pyridine, tert-butylammonium fluoride (TBAF), hexane, triethylamine (TEA), imidazole, dichlorobenzoylchloride, *N*,*N*'- diisopropylcarbodiimide (DIC), bromoacetic acid, piperidine, potassium cyanide, ninhydrin, phenol, 4- dimethyl-aminopyridine (DMAP), trifluoroacetic acid (TFA), dichloromethane (DCM), diethyl ether,

acetone, sodium bicarbonate, anhydrous sodium sulfate, chloroform, hydrochloric acid, ethyl acetate and *N*-hydroxysuccinimide (NHS), were obtained from Fluorochem Ltd. (Hadfield, UK). Succinic anhydride, benzylamine, triisopropylsilane (TIPS) and thioanisole were purchased from Alfa Aesar (Alfa Aesar, Heysham, Lancashire, UK). Zidovudine (1-[(2R,4S,5S)-4-azido-5-(hydroxymethyl)oxolan-2-yl]-5-methylpyrimidine-2,4-dione) was obtained from TCI Chemicals (Tokyo Chemical Industry UK Ltd, Oxford, UK). Büchi rotavapor (Büchi UK Ltd., Chadderton, Oldham, UK).

S.2. Synthesis methods and analysis of peptoid-peptides

S.2.1. Synthesis: Solid-phase peptide synthesis proceeded as a series of deprotection and addition reactions as per standard solid phase protocols,[1] and anchoring of Fmoc-amino acids to the resin via the dichlorobenzoyl chloride method,[2] described in the supporting information of our previously reported work.[3] The required peptoid sequence was then added using a solid-phase submonomer synthetic protocol described below involving a series of repeated bromoacetylation and displacement reactions.[4]

The structures of all the synthesized peptoid-peptide sequences are shown in Figure S1. L and D enantiomers of Fmoc-O-phosphotyrosine were utilized to provide a phosphate grouping that triggered hydrogel formation upon removal.[2] Zidovudine was attached to the side chain ε-amino group of L- or D-lysine by the addition and subsequent activation of succinic anhydride which enabled direct attachment of the drug to the peptoid-peptide's ε-amino group via ester linkage as previously described.[3, 5] The purity of each product was analyzed by HPLC (> 95%) as shown in Figures S19 – S22. Peptoid-peptides were lyophilized in an Edwards freeze drier with an RV8 pump (Davidson and Hardy, Belfast, Co. Antrim, UK) and successful synthesis (identity) was confirmed using ^{1}H-NMR (Bruker Ultrashield Plus 400 MHz, Bruker, Coventry,

UK; Figures S2 – S8) and electrospray ionization mass spectrometry (ESI-MS) (Waters LCT Premier, Waters, Hertfordshire, UK; Figures S16 – S18). ^{31}P-NMR was also performed to confirm presence of phosphate groups on the tyrosine residue (Figures S9 – S15).

Bromoacetylation: The secondary amine on the amino terminal of the peptide sequence is first acetylated using bromoacetic acid and DIC. 4 molar equivalents (meq) of bromoacetic acid and 4 meq of DIC were added to the synthesized peptide sequence in DMF and reacted for 2 hours by bubbling under nitrogen. This was followed by three DMF rinses.

Displacement: The bromine is then displaced by the primary amine to afford chain elongation. 4 meq of benzylamine in DMF was added and reacted for 2 hours by bubbling under nitrogen to afford chain elongation. This was followed by three DMF rinses.

These two steps were repeated until the desired sequence was synthesized, then rinsed three times with DMF, five times with DCM, five times with methanol and dried overnight in a desiccator. The desired sequence was then cleaved from the resin as previously described.[3]

Zidovudine conjugation: Zidovudine was modified in a two stage process to generate a functional group enabling direct attachment to the lysine side chain via ester linkage with the desired sequence as previously described.[3]

Table S1. Peptoid/peptoid-peptide sequences synthesized, the resin employed as the support for synthesis, the percentage yield and whether propensity to gelate was observed or not. Gelation propensity was tested by the addition of alkaline phosphatase at a concentration of 2 U with overnight incubation at 37°C, formulated as outlined in Table S2.

**Sequence**	**Resin**	**Yield (%)**	**Gelation propensity**
(*N*Phe)$_4$(*N*Lys)Y(p)-OH	2-Chlorotrityl Chloride (100 mg, 0.3 – 0.6 mmol)	25.3	No*
(*N*Phe)$_4$(*N*Lys)Y(p)-OH	Wang (500 mg, 0.3 – 0.8 mmol)	8	No*
(*N*Phe)$_4$(*N*Lys)Y(p)G-OH	Fmoc-Gly-Wang (1g, 0.3 – 0.8 mmol)	25	No*
(*N*Phe)$_4$(*N*Lys)Y(p)GGG-OH	Fmoc-Gly-Wang (1 g, 0.3 – 0.8 mmol)	91	No*
(*N*Phe)$_4$GGGGKY(p)-OH	Wang (1g, 1 mmol)	88	Yes
(*N*Phe)$_4$GGGGky(p)-OH	Wang (1g, 1 mmol)	80.5	Yes

* In case of non-gelation upon the addition of 2 U of alkaline phosphatase and overnight incubation at 37°C, fresh samples were heated to 70°C and then cooled to room temperature (25°C) prior to the addition of alkaline phosphatase to assess propensity to gelate.

a)

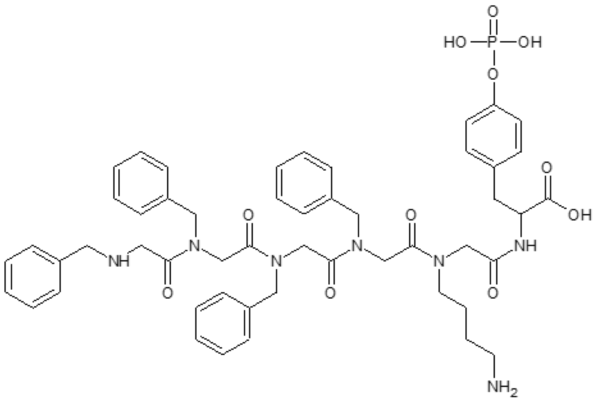

b)

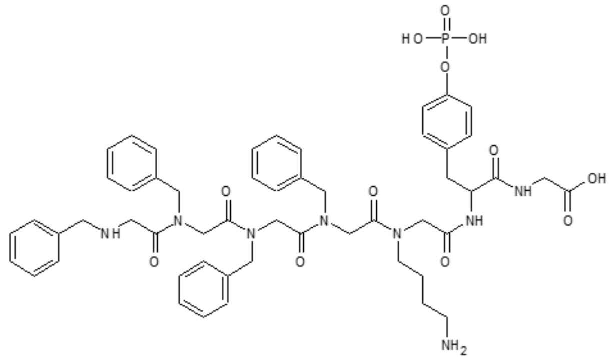

c)

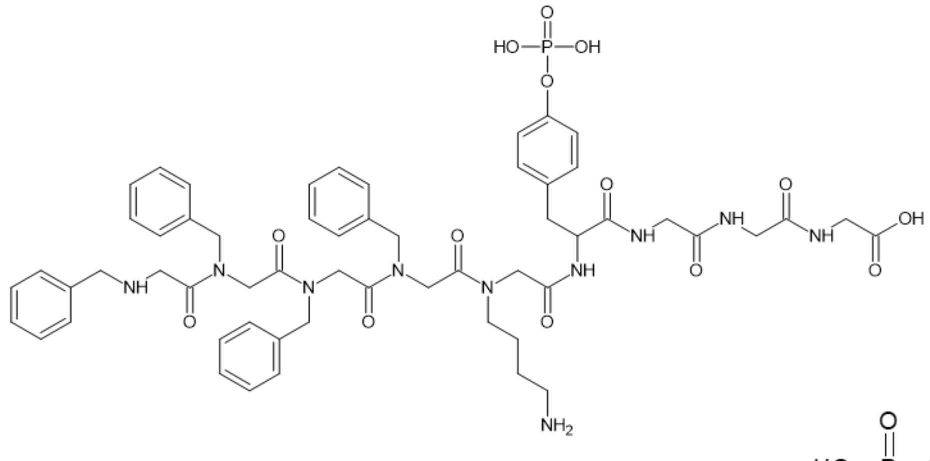

d)

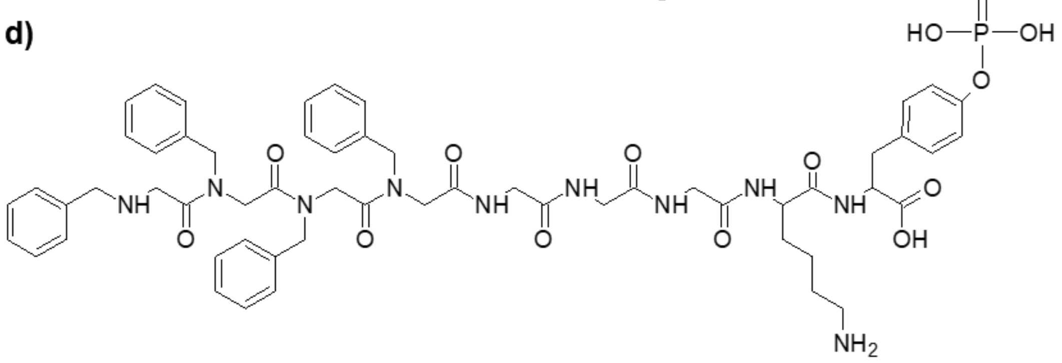

Figure S1. Peptoid-peptide sequences synthesized. Single letter amino acid nomenclature employed. a) (*N*Phe)$_4$(*N*Lys)Y(p)-OH, b) (*N*Phe)$_4$(*N*Lys)Y(p)G-OH, c) (*N*Phe)$_4$(*N*Lys)Y(p)GGG-OH d) (*N*Phe)$_4$GGGGKY(p)-OH.

(*N*Phe)$_4$(*N*Lys)Y(p)-OH

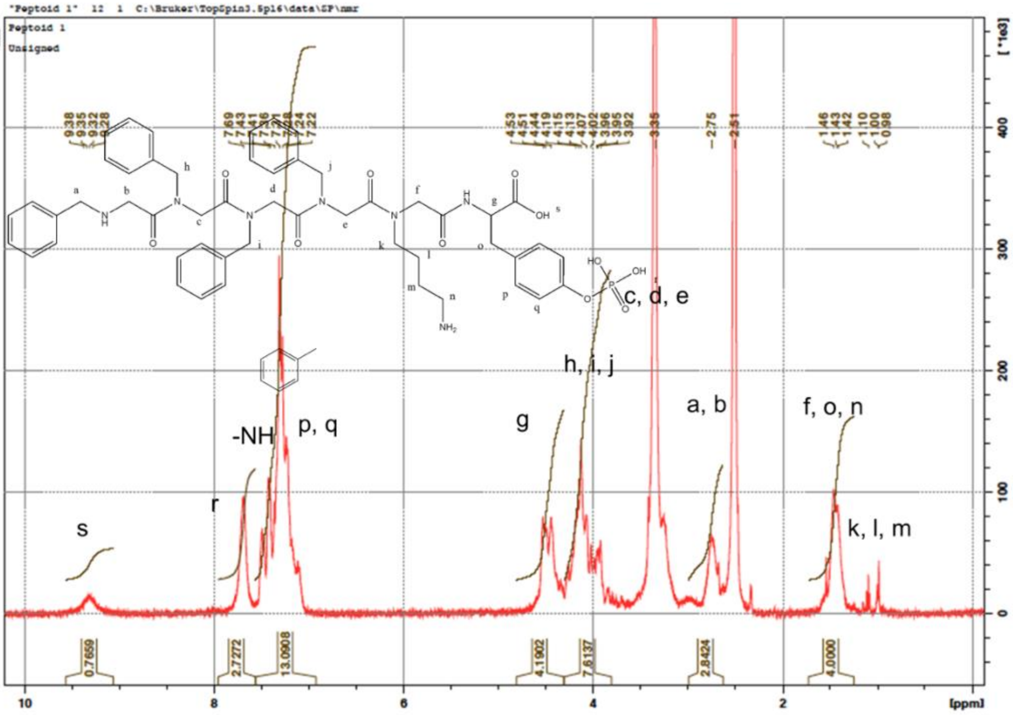

Figure S2. ^{1}H NMR trace for (*N*Phe)$_4$(*N*Lys)Y(p)-OH in DMSO-d_6.

^{1}H NMR (C_2D_6OS, TMS standard, 400 MHZ): δ 1.47-1.70 (4H, 1.54 (tt, J = 7.4, 7.3 Hz), 1.54 (tt, J = 7.4, 7.3 Hz), 1.64 (tt, J = 7.4, 7.2 Hz), 1.64 (tt, J = 7.4, 7.2 Hz)), 2.57-2.82 (6H, 2.63 (t, J = 7.3 Hz), 2.63 (t, J = 7.3 Hz), 2.74 (d, J = 7.7 Hz), 2.74 (d, J = 7.7 Hz), 2.76 (d, J = 7.5 Hz), 2.76 (d, J = 7.5 Hz)), 2.92 (1H, tt, J = 7.7, 7.5 Hz), 3.25-3.37 (2H, 3.31 (t, J = 7.2 Hz), 3.31 (t, J = 7.2 Hz)), 3.64-3.74 (2H, 3.69 (s), 3.69 (s)), 3.76-3.95 (8H, 3.81 (s), 3.81 (s), 3.89 (s), 3.89 (s), 3.90 (s), 3.90 (s), 3.90 (s), 3.90 (s)), 3.95-4.05 (2H, 4.00 (s), 4.00 (s)), 4.49-4.62 (6H, 4.54 (s), 4.54 (s), 4.55 (s), 4.55 (s), 4.57 (s), 4.57 (s)), 6.90-7.14 (10H, 6.97 (ddd, J = 8.3, 1.6, 0.5 Hz), 7.08 (dddd, J = 7.7, 1.3, 1.2, 0.5 Hz), 7.08 (dddd, J = 7.7, 1.3, 1.2, 0.5 Hz), 7.08 (dddd, J = 7.7, 1.3, 1.2, 0.5 Hz), 7.08 (ddd, J = 8.3, 1.2, 0.5 Hz)), 7.20-7.38 (14H, 7.26 (tt, J = 7.7, 1.3 Hz), 7.26 (tt, J = 7.7, 1.3 Hz), 7.27 (tdd, J = 7.7, 1.9, 0.5 Hz), 7.27 (tdd, J = 7.7, 1.9, 0.5 Hz), 7.28 (dddd, J = 7.7, 1.4, 0.9, 0.5 Hz), 7.29 (tdd, J = 7.7, 1.8, 0.5 Hz), 7.31 (tdd, J = 7.7, 1.8, 0.5 Hz), 7.31 (tt, J = 7.7, 1.4 Hz), 7.32 (tt, J = 7.7, 1.3 Hz)).

(*N*Phe)$_4$(*N*Lys)Y(p)G-OH

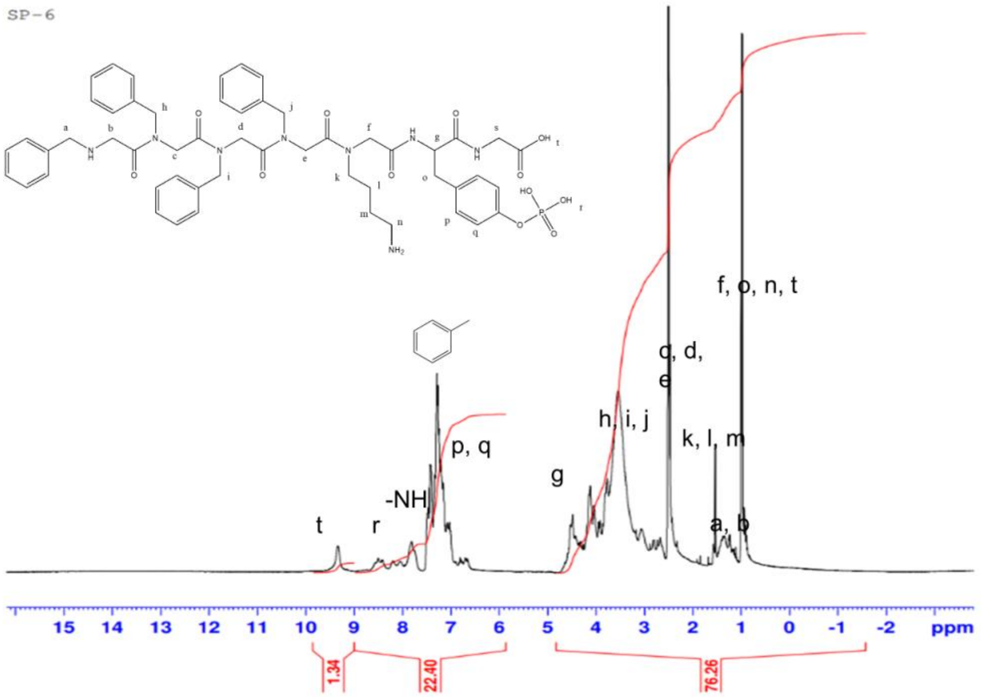

Figure S3. ^{1}H NMR trace for (*N*Phe)$_4$(*N*Lys)Y(p)G-OH in DMSO-d_6.

^{1}H NMR (C_2D_6OS, TMS standard, 400 MHZ): δ 1.47-1.70 (4H, 1.54 (tt, *J* = 7.4, 7.3 Hz), 1.54 (tt, *J* = 7.4, 7.3 Hz), 1.64 (tt, *J* = 7.4, 7.2 Hz), 1.64 (tt, *J* = 7.4, 7.2 Hz)), 2.57-2.97 (7H, 2.63 (t, *J* = 7.3 Hz), 2.63 (t, *J* = 7.3 Hz), 2.73 (d, *J* = 7.7 Hz), 2.73 (d, *J* = 7.7 Hz), 2.79 (d, *J* = 7.5 Hz), 2.79 (d, *J* = 7.5 Hz), 2.90 (tt, *J* = 7.7, 7.5 Hz)), 3.25-3.37 (2H, 3.31 (t, *J* = 7.2 Hz), 3.31 (t, *J* = 7.2 Hz)), 3.64-3.95 (12H, 3.69 (s), 3.69 (s), 3.74 (s), 3.74 (s), 3.81 (s), 3.81 (s), 3.89 (s), 3.89 (s), 3.90 (s), 3.90 (s), 3.90 (s), 3.90 (s)), 3.96-4.05 (2H, 4.00 (s), 4.00 (s)), 4.49-4.62 (6H, 4.54 (s), 4.54 (s), 4.57 (s), 4.57 (s), 4.57 (s), 4.57 (s)), 6.90-7.14 (10H, 6.97 (ddd, *J* = 8.3, 1.6, 0.5 Hz), 7.07 (ddd, *J* = 8.3, 1.2, 0.5 Hz), 7.08 (dddd, *J* = 7.7, 1.3, 1.2, 0.5 Hz), 7.08 (dddd, *J* = 7.7, 1.3, 1.2, 0.5 Hz), 7.08 (dddd, *J* = 7.7, 1.3, 1.2, 0.5 Hz)), 7.20-7.38 (14H, 7.26 (tt, *J* = 7.7, 1.3 Hz), 7.26 (tt, *J* = 7.7, 1.3 Hz), 7.27 (tdd, *J* = 7.7, 1.9, 0.5 Hz), 7.27 (tdd, *J* = 7.7, 1.9, 0.5 Hz), 7.28 (dddd, *J* = 7.7, 1.4, 0.9, 0.5 Hz), 7.29 (tdd, *J* = 7.7, 1.8, 0.5 Hz), 7.31 (tdd, *J* = 7.7, 1.8, 0.5 Hz), 7.31 (tt, *J* = 7.7, 1.4 Hz), 7.32 (tt, *J* = 7.7, 1.3 Hz)).

(NPhe)$_4$(NLys)Y(p)GGG-OH

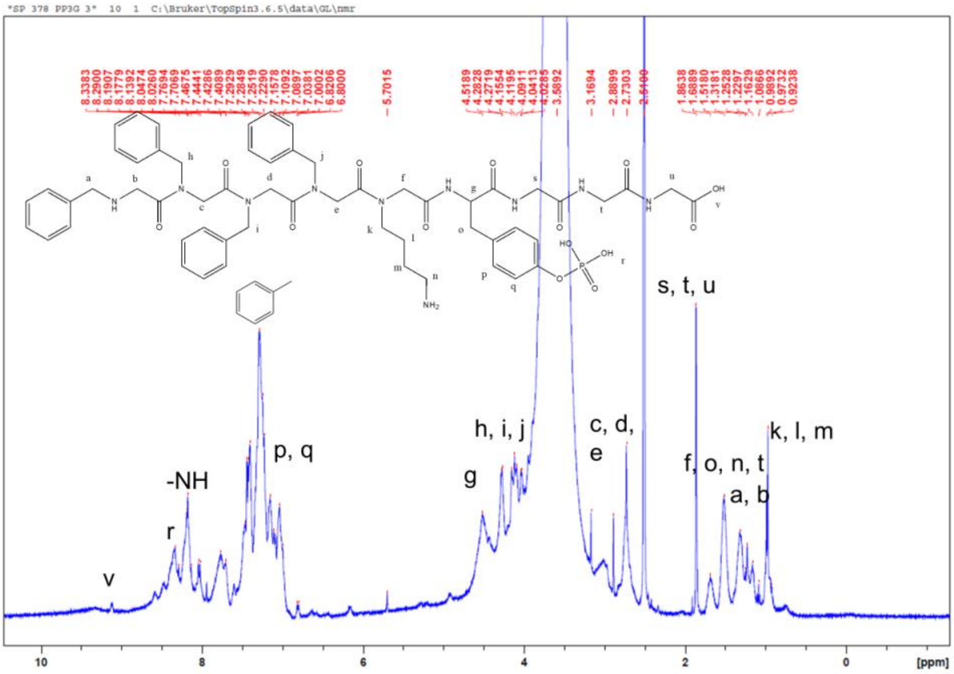

Figure S4. ^{1}H NMR trace for (NPhe)$_4$(NLys)Y(p)GGG-OH in DMSO-d_6.

^{1}H NMR (C_2D_6OS, TMS standard, 400 MHZ): δ 1.47-1.70 (4H, 1.54 (tt, J = 7.4, 7.3 Hz), 1.54 (tt, J = 7.4, 7.3 Hz), 1.64 (tt, J = 7.4, 7.2 Hz), 1.64 (tt, J = 7.4, 7.2 Hz)), 2.57-2.97 (7H, 2.63 (t, J = 7.3 Hz), 2.63 (t, J = 7.3 Hz), 2.73 (d, J = 7.7 Hz), 2.73 (d, J = 7.7 Hz), 2.79 (d, J = 7.5 Hz), 2.79 (d, J = 7.5 Hz), 2.90 (tt, J = 7.7, 7.5 Hz)), 3.25-3.37 (2H, 3.31 (t, J = 7.2 Hz), 3.31 (t, J = 7.2 Hz)), 3.64-3.95 (16H, 3.69 (s), 3.69 (s), 3.72 (s), 3.72 (s), 3.73 (s), 3.73 (s), 3.75 (s), 3.75 (s), 3.81 (s), 3.81 (s), 3.89 (s), 3.89 (s), 3.90 (s), 3.90 (s), 3.90 (s), 3.90 (s)), 3.96-4.05 (2H, 4.00 (s), 4.00 (s)), 4.49-4.62 (6H, 4.54 (s), 4.54 (s), 4.57 (s), 4.57 (s), 4.57 (s), 4.57 (s)), 6.90-7.14 (10H, 6.97 (ddd, J = 8.3, 1.6, 0.5 Hz), 7.07 (ddd, J = 8.3, 1.2, 0.5 Hz), 7.08 (dddd, J = 7.7, 1.3, 1.2, 0.5 Hz), 7.08 (dddd, J = 7.7, 1.3, 1.2, 0.5 Hz), 7.08 (dddd, J = 7.7, 1.3, 1.2, 0.5 Hz)), 7.20-7.38 (14H, 7.26 (tt, J = 7.7, 1.3 Hz), 7.26 (tt, J = 7.7, 1.3 Hz), 7.27 (tdd, J = 7.7, 1.9, 0.5 Hz), 7.27 (tdd, J = 7.7, 1.9, 0.5 Hz), 7.28 (dddd, J = 7.7, 1.4, 0.9, 0.5 Hz), 7.29 (tdd, J = 7.7, 1.8, 0.5 Hz), 7.31 (tdd, J = 7.7, 1.8, 0.5 Hz), 7.31 (tt, J = 7.7, 1.4 Hz), 7.32 (tt, J = 7.7, 1.3 Hz)).

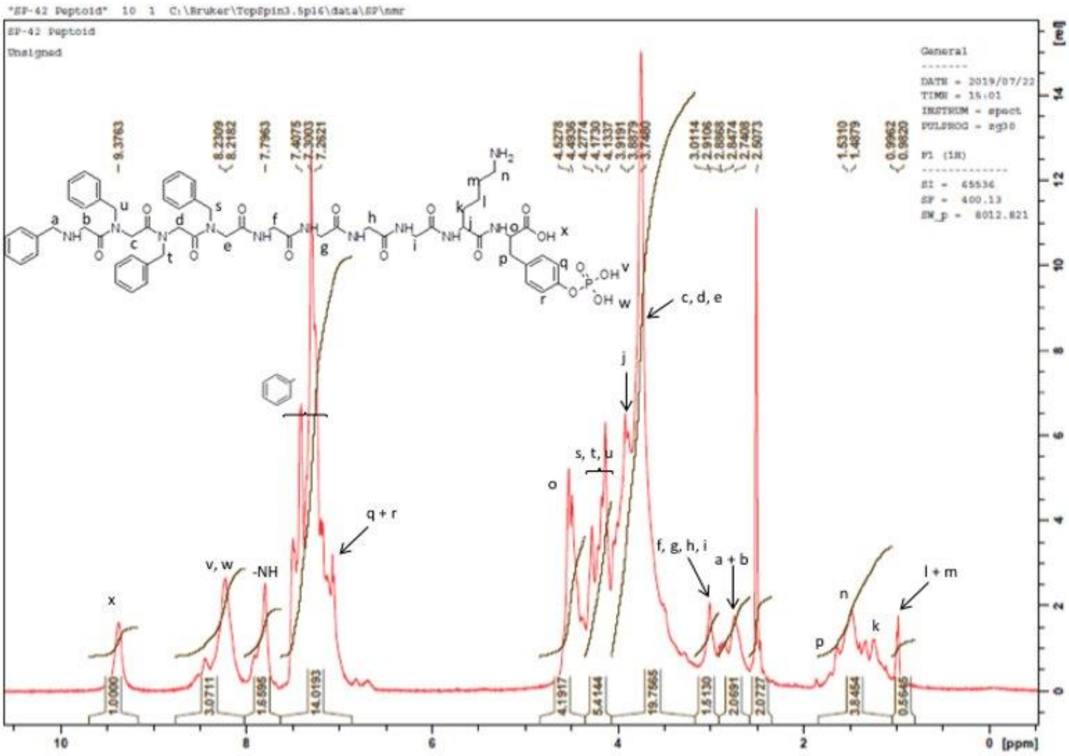

Figure S5. ^{1}H NMR trace assigned for (*N*Phe)$_4$GGGGKY(p)-OH in DMSO-d_6.

^{1}H NMR (C_2D_6OS, TMS standard, 400 MHZ): δ 1.32-1.58 (4H, 1.39 (tt, J = 7.8, 7.4 Hz), 1.39 (tt, J = 7.8, 7.4 Hz), 1.51 (tt, J = 7.4, 7.3 Hz), 1.51 (tt, J = 7.4, 7.3 Hz)), 1.85-1.99 (2H, 1.92 (td, J = 7.8, 7.5 Hz), 1.92 (td, J = 7.8, 7.5 Hz)), 2.57-2.69 (2H, 2.63 (t, J = 7.3 Hz), 2.63 (t, J = 7.3 Hz)), 2.74-2.85 (2H, 2.79 (d, J = 6.9 Hz), 2.79 (d, J = 6.9 Hz)), 3.64-3.95 (18H, 3.69 (s), 3.69 (s), 3.73 (s), 3.73 (s), 3.74 (s), 3.74 (s), 3.75 (s), 3.75 (s), 3.75 (s), 3.75 (s), 3.81 (s), 3.81 (s), 3.88 (s), 3.88 (s), 3.89 (s), 3.89 (s), 3.90 (s), 3.90 (s)), 4.35 (1H, t, J = 7.5 Hz), 4.49-4.73 (7H, 4.54 (s), 4.54 (s), 4.54 (s), 4.54 (s), 4.57 (s), 4.57 (s), 4.67 (t, J = 6.9 Hz)), 6.91-7.14 (10H, 6.97 (ddd, J = 8.3, 1.6, 0.5 Hz), 7.03 (ddd, J = 8.3, 1.2, 0.5 Hz), 7.08 (dddd, J = 7.7, 1.3, 1.2, 0.5 Hz), 7.08 (dddd, J = 7.7, 1.3, 1.2, 0.5 Hz), 7.08 (dddd, J = 7.7, 1.3, 1.2, 0.5 Hz)), 7.20-7.38 (14H, 7.26 (tt, J = 7.7, 1.3 Hz), 7.27 (tdd, J = 7.7, 1.9, 0.5 Hz), 7.28 (dddd, J = 7.7, 1.4, 0.9, 0.5 Hz), 7.29 (tdd, J = 7.7, 1.8, 0.5 Hz), 7.29 (tdd, J = 7.7, 1.8, 0.5 Hz), 7.31 (tdd, J = 7.7, 1.8, 0.5 Hz), 7.31 (tt, J = 7.7, 1.4 Hz), 7.32 (tt, J = 7.7, 1.3 Hz), 7.32 (tt, J = 7.7, 1.3 Hz)).

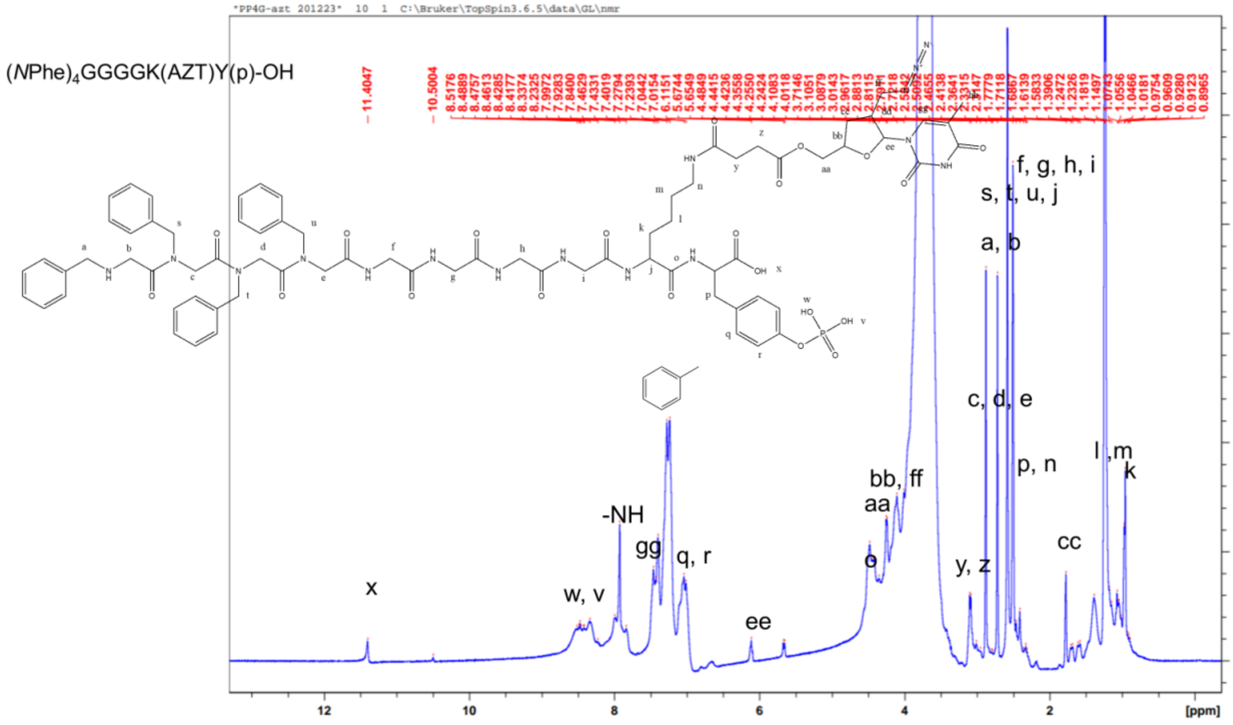

Figure S6. ^{1}H NMR trace assigned for (*N*Phe)$_4$GGGGK(AZT)Y(p)-OH in DMSO-d_6.

^{1}H NMR (C_2D_6OS, TMS standard, 400 MHZ): δ 1.21-1.34 (2H, 1.27 (tt, *J* = 7.4, 7.3 Hz), 1.27 (tt, *J* = 7.4, 7.3 Hz)), 1.49-1.63 (2H, 1.56 (tt, *J* = 7.3, 7.0 Hz), 1.56 (tt, *J* = 7.3, 7.0 Hz)), 1.79-2.11 (7H, 1.88 (ddd, *J* = 14.0, 9.3, 7.3 Hz), 1.86 (s), 1.92 (q, *J* = 7.5 Hz), 1.92 (q, *J* = 7.5 Hz), 2.03 (ddd, *J* = 14.0, 7.2, 5.5 Hz)), 2.66-2.85 (6H, 2.72 (t, *J* = 7.4 Hz), 2.72 (t, *J* = 7.4 Hz), 2.72 (t, *J* = 7.4 Hz), 2.72 (t, *J* = 7.4 Hz), 2.79 (d, *J* = 6.9 Hz), 2.79 (d, *J* = 6.9 Hz)), 2.97 (1H, ddddd, *J* = 8.1, 7.3, 7.2, 5.3, 5.1 Hz), 3.13-3.24 (2H, 3.19 (t, *J* = 7.0 Hz), 3.19 (t, *J* = 7.0 Hz)), 3.64-3.99 (21H, 3.69 (s), 3.69 (s), 3.73 (s), 3.73 (s), 3.74 (s), 3.74 (s), 3.75 (s), 3.75 (s), 3.75 (s), 3.75 (s), 3.81 (s), 3.81 (s), 3.86 (d, *J* = 5.1 Hz), 3.88 (s), 3.88 (s), 3.89 (s), 3.89 (s), 3.90 (s), 3.90 (s), 3.92 (ddt, *J* = 9.3, 5.5, 3.8 Hz), 3.91 (d, *J* = 5.3 Hz)), 4.35 (1H, t, *J* = 7.5 Hz), 4.43-4.73 (9H, 4.49 (d, *J* = 3.8 Hz), 4.49 (d, *J* = 3.8 Hz), 4.54 (s), 4.54 (s), 4.54 (s), 4.54 (s), 4.57 (s), 4.57 (s), 4.67 (t, *J* = 6.9 Hz)), 6.16 (1H, d, *J* = 8.1 Hz), 6.91-7.14 (10H, 6.97 (ddd, *J* = 8.3, 1.6, 0.5 Hz), 7.03 (ddd, *J* = 8.3, 1.2, 0.5 Hz), 7.08 (dddd, *J* = 7.7, 1.3, 1.2, 0.5 Hz), 7.08 (dddd, *J* = 7.7, 1.3, 1.2, 0.5 Hz), 7.08 (dddd, *J* = 7.7, 1.3, 1.2, 0.5 Hz)), 7.17-7.38 (14H, 7.25 (dddd, *J* = 7.5, 7.1, 1.4, 0.5 Hz), 7.26 (tt, *J* = 7.7, 1.3 Hz), 7.27 (tdd, *J* = 7.7, 1.9, 0.5 Hz), 7.28

(dddd, *J* = 7.5, 1.3, 0.9, 0.5 Hz), 7.29 (tdd, *J* = 7.7, 1.8, 0.5 Hz), 7.29 (tdd, *J* = 7.7, 1.8, 0.5 Hz), 7.31 (tdd, *J* = 7.1, 1.4, 1.2 Hz), 7.32 (tt, *J* = 7.7, 1.3 Hz), 7.32 (tt, *J* = 7.7, 1.3 Hz)), 7.58 (1H, s).

(*N*Phe)$_4$GGGGky(p)-OH

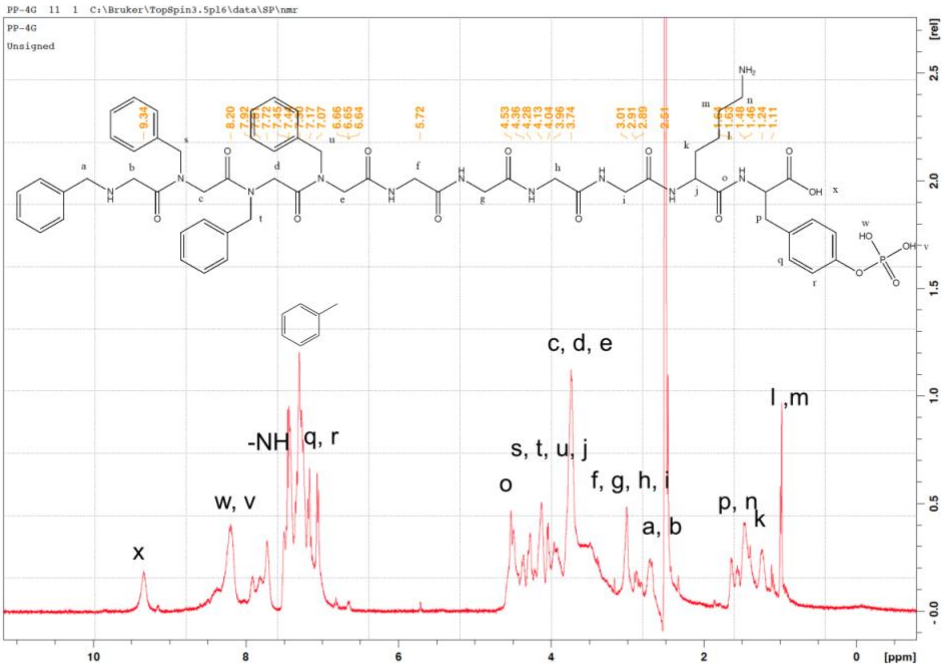

Figure S7. ^{1}H NMR trace assigned for (*N*Phe)$_4$GGGGky(p)-OH in DMSO-d_6.

^{1}H NMR(C_2D_6OS, TMS standard, 400 MHZ) : δ 1.32-1.58 (4H, 1.39 (tt, J = 7.8, 7.4 Hz), 1.39 (tt, J = 7.8, 7.4 Hz), 1.51 (tt, J = 7.4, 7.3 Hz), 1.51 (tt, J = 7.4, 7.3 Hz)), 1.85-1.99 (2H, 1.92 (td, J = 7.8, 7.5 Hz), 1.92 (td, J = 7.8, 7.5 Hz)), 2.57-2.69 (2H, 2.63 (t, J = 7.3 Hz), 2.63 (t, J = 7.3 Hz)), 2.74-2.85 (2H, 2.79 (d, J = 6.9 Hz), 2.79 (d, J = 6.9 Hz)), 3.64-3.95 (18H, 3.69 (s), 3.69 (s), 3.73 (s), 3.73 (s), 3.74 (s), 3.74 (s), 3.75 (s), 3.75 (s), 3.75 (s), 3.75 (s), 3.81 (s), 3.81 (s), 3.88 (s), 3.88 (s), 3.89 (s), 3.89 (s), 3.90 (s), 3.90 (s)), 4.35 (1H, t, J = 7.5 Hz), 4.49-4.73 (7H, 4.54 (s), 4.54 (s), 4.54 (s), 4.54 (s), 4.57 (s), 4.57 (s), 4.67 (t, J = 6.9 Hz)), 6.91-7.14 (10H, 6.97 (ddd, J = 8.3, 1.6, 0.5 Hz), 7.03 (ddd, J = 8.3, 1.2, 0.5 Hz), 7.08 (dddd, J = 7.7, 1.3, 1.2, 0.5 Hz), 7.08 (dddd, J = 7.7, 1.3, 1.2, 0.5 Hz), 7.08 (dddd, J = 7.7, 1.3, 1.2, 0.5 Hz)), 7.20-7.38 (14H, 7.26 (tt, J = 7.7, 1.3 Hz), 7.27 (tdd, J = 7.7, 1.9, 0.5 Hz), 7.28 (dddd, J = 7.7, 1.4, 0.9, 0.5

Hz), 7.29 (tdd, J = 7.7, 1.8, 0.5 Hz), 7.29 (tdd, J = 7.7, 1.8, 0.5 Hz), 7.31 (tdd, J = 7.7, 1.8, 0.5 Hz), 7.31 (tt, J = 7.7, 1.4 Hz), 7.32 (tt, J = 7.7, 1.3 Hz), 7.32 (tt, J = 7.7, 1.3 Hz)).

(*N*Phe)$_4$GGGGk(AZT)y(p)-OH

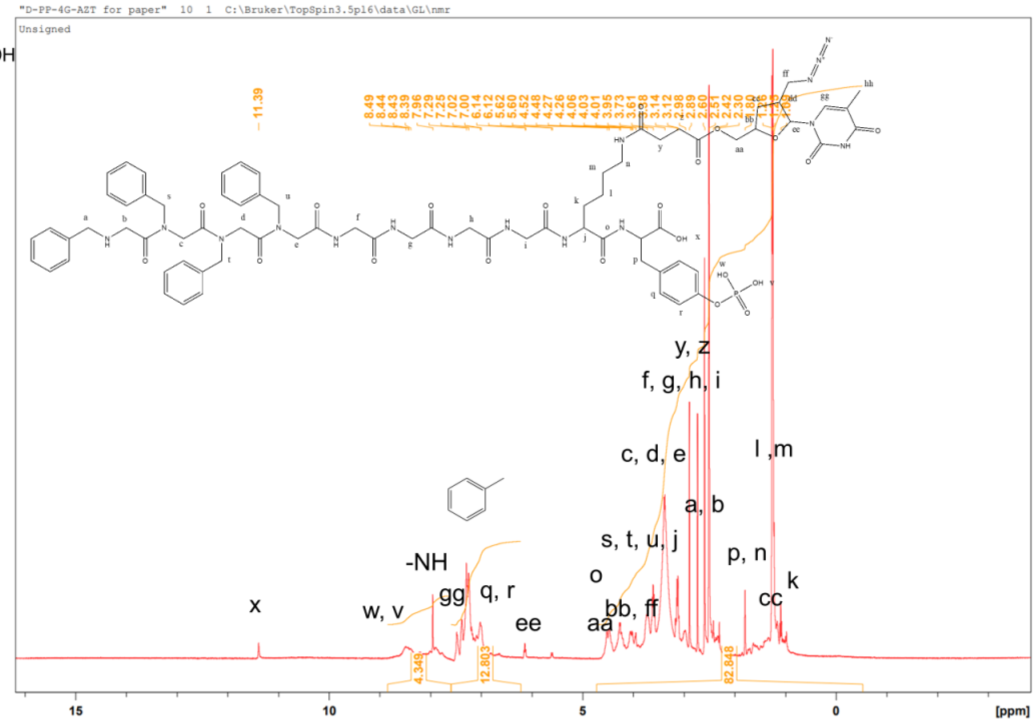

Figure S8. ^{1}H NMR trace assigned for (*N*Phe)$_4$GGGGk(AZT)y(p)-OH in DMSO-*d*$_6$.

^{1}H NMR (C_2D_6OS, TMS standard, 400 MHZ): δ 1.21-1.34 (2H, 1.27 (tt, *J* = 7.4, 7.3 Hz), 1.27 (tt, *J* = 7.4, 7.3 Hz)), 1.49-1.63 (2H, 1.56 (tt, *J* = 7.3, 7.0 Hz), 1.56 (tt, *J* = 7.3, 7.0 Hz)), 1.79-2.11 (7H, 1.88 (ddd, *J* = 14.0, 9.3, 7.3 Hz), 1.86 (s), 1.92 (q, *J* = 7.5 Hz), 1.92 (q, *J* = 7.5 Hz), 2.03 (ddd, *J* = 14.0, 7.2, 5.5 Hz)), 2.66-2.85 (6H, 2.72 (t, *J* = 7.4 Hz), 2.72 (t, *J* = 7.4 Hz), 2.72 (t, *J* = 7.4 Hz), 2.72 (t, *J* = 7.4 Hz), 2.79 (d, *J* = 6.9 Hz), 2.79 (d, *J* = 6.9 Hz)), 2.97 (1H, ddddd, *J* = 8.1, 7.3, 7.2, 5.3, 5.1 Hz), 3.13-3.24 (2H, 3.19 (t, *J* = 7.0 Hz), 3.19 (t, *J* = 7.0 Hz)), 3.64-3.99 (21H, 3.69 (s), 3.69 (s), 3.73 (s), 3.73 (s), 3.74 (s), 3.74 (s), 3.75 (s), 3.75 (s), 3.75 (s), 3.75 (s), 3.81 (s), 3.81 (s), 3.86 (d, *J* = 5.1 Hz), 3.88 (s), 3.88 (s), 3.89 (s), 3.89 (s), 3.90 (s), 3.90 (s), 3.92 (ddt, *J* = 9.3, 5.5, 3.8 Hz), 3.91 (d, *J* = 5.3 Hz)), 4.35 (1H, t, *J* = 7.5 Hz), 4.43-4.73 (9H, 4.49 (d, *J* = 3.8 Hz), 4.49 (d, *J* = 3.8 Hz), 4.54 (s), 4.54 (s), 4.54 (s), 4.54 (s), 4.57 (s), 4.57 (s), 4.67 (t, *J* = 6.9 Hz)), 6.16 (1H, d, *J* = 8.1 Hz), 6.91-7.14 (10H, 6.97 (ddd, *J* = 8.3,

1.6, 0.5 Hz), 7.03 (ddd, J = 8.3, 1.2, 0.5 Hz), 7.08 (dddd, J = 7.7, 1.3, 1.2, 0.5 Hz), 7.08 (dddd, J = 7.7, 1.3, 1.2, 0.5 Hz), 7.08 (dddd, J = 7.7, 1.3, 1.2, 0.5 Hz)), 7.17-7.38 (14H, 7.25 (dddd, J = 7.5, 7.1, 1.4, 0.5 Hz), 7.26 (tt, J = 7.7, 1.3 Hz), 7.27 (tdd, J = 7.7, 1.9, 0.5 Hz), 7.28 (dddd, J = 7.5, 1.3, 0.9, 0.5 Hz), 7.29 (tdd, J = 7.7, 1.8, 0.5 Hz), 7.29 (tdd, J = 7.7, 1.8, 0.5 Hz), 7.31 (tdd, J = 7.1, 1.4, 1.2 Hz), 7.32 (tt, J = 7.7, 1.3 Hz), 7.32 (tt, J = 7.7, 1.3 Hz)), 7.58 (1H, s).

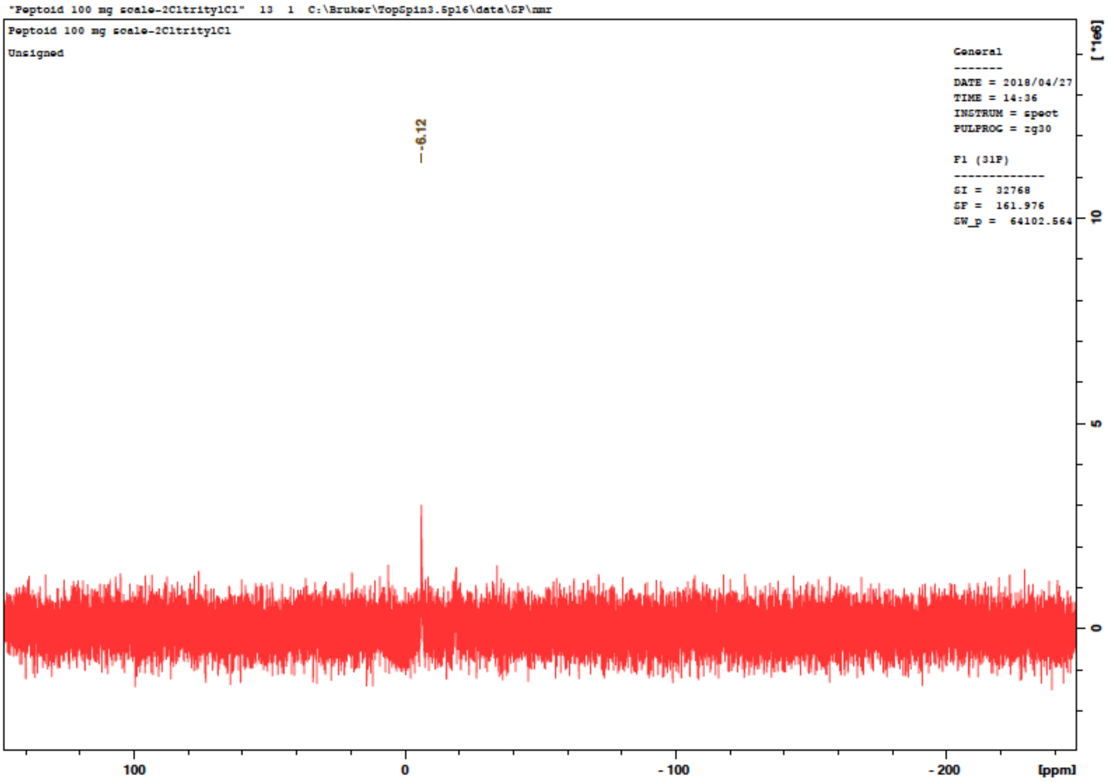

Figure S9. ^{31}P NMR trace for (NPhe)$_4$(NLys)Y(p)-OH, peak at -6.12 demonstrates the presence and retention of the phosphate grouping on the tyrosine motif.

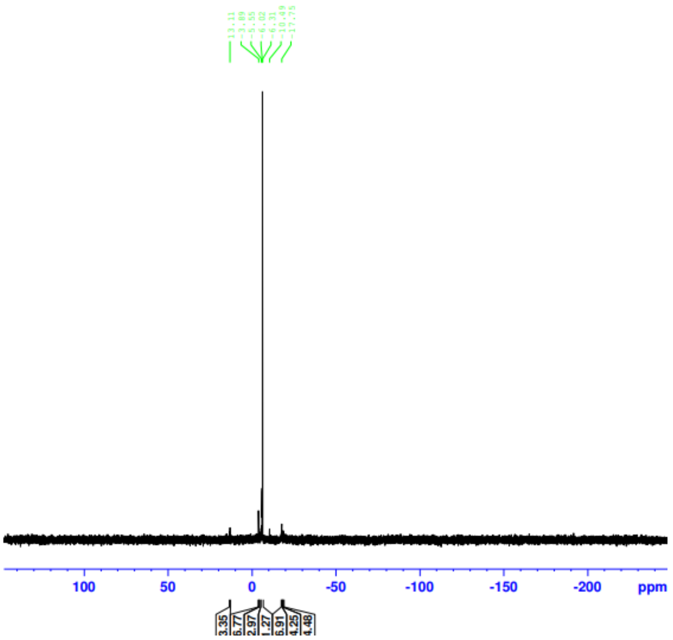

Figure S10. ^{31}P NMR trace for (*N*Phe)$_4$(*N*Lys)Y(p)G-OH, peak at -6.02 demonstrates the presence and retention of the phosphate grouping on the tyrosine motif.

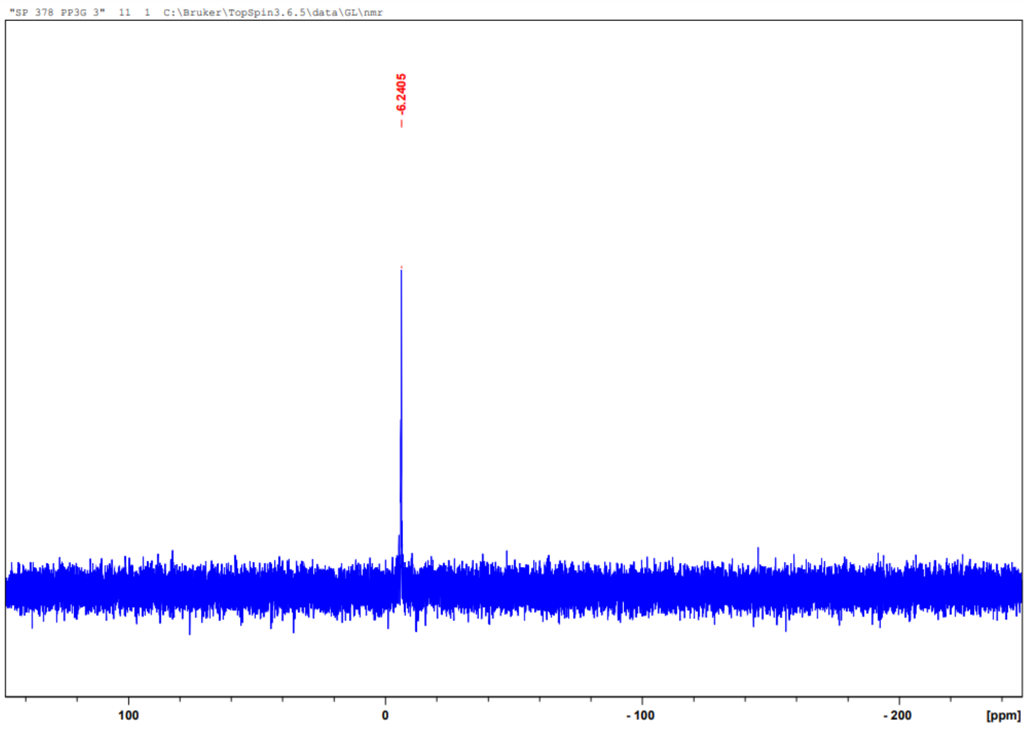

Figure S11. ^{31}P NMR trace for (*N*Phe)$_4$(*N*Lys)Y(p)GGG-OH, peak at 6.24 demonstrates the presence and retention of the phosphate grouping on the tyrosine motif.

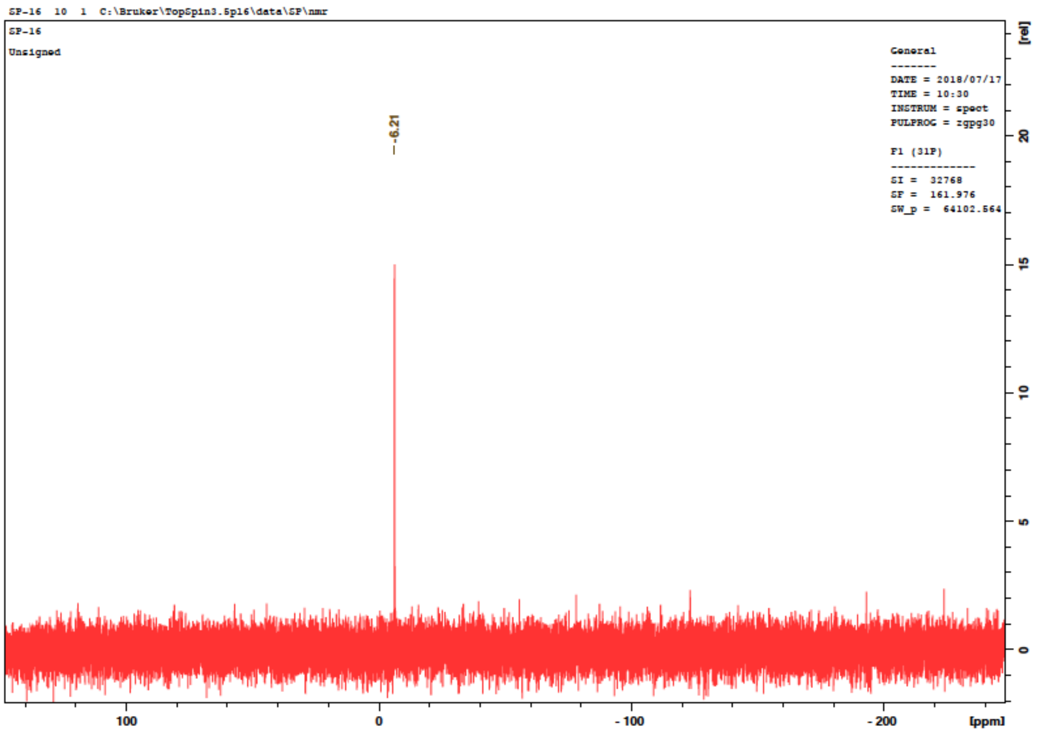

Figure S12. ^{31}P NMR trace for (*N*Phe)$_4$GGGGKY(p)-OH, peak at -6.21 demonstrates the presence and retention of the phosphate grouping on the tyrosine motif.

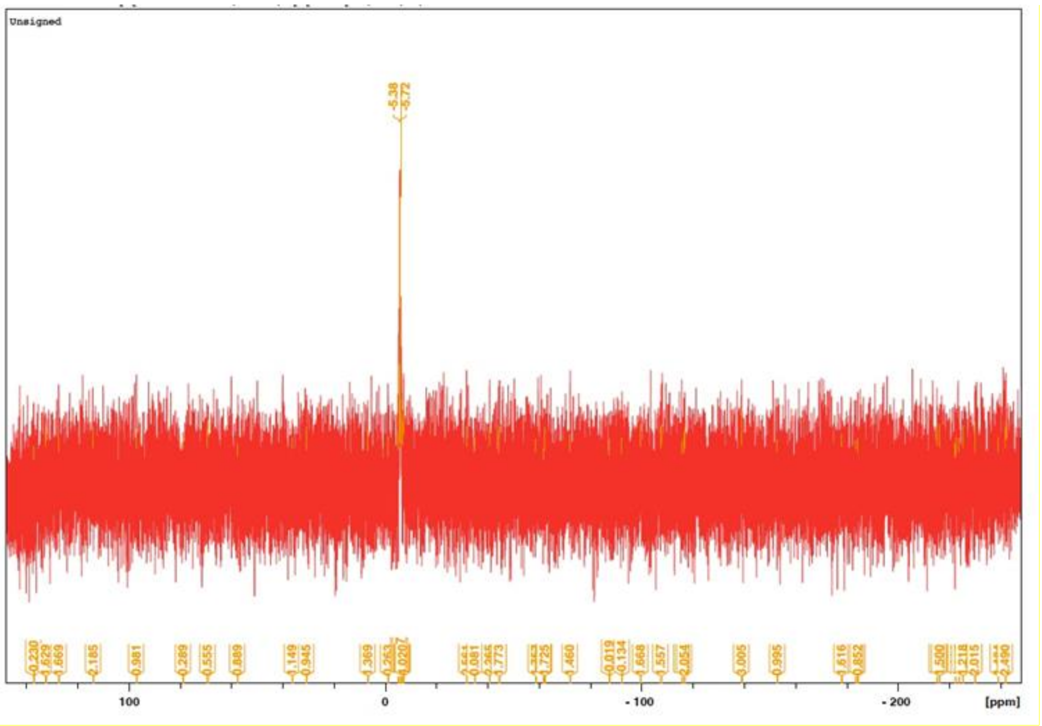

Figure S13. ^{31}P NMR trace for (*N*Phe)$_4$GGGGK(AZT)Y(p)-OH peak at -5.72 demonstrates the presence and retention of the phosphate grouping on the tyrosine motif.

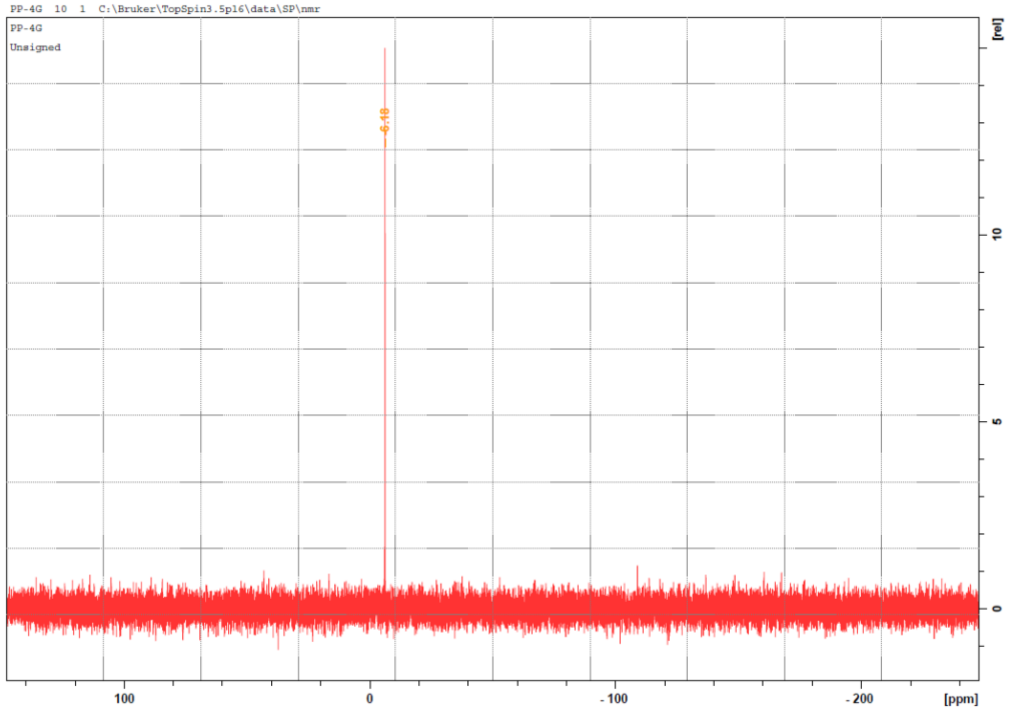

Figure S14. ^{31}P NMR trace (*N*Phe)$_4$GGGGky(p)-OH, peak at -6.18 demonstrates the presence and retention of the phosphate grouping on the tyrosine motif.

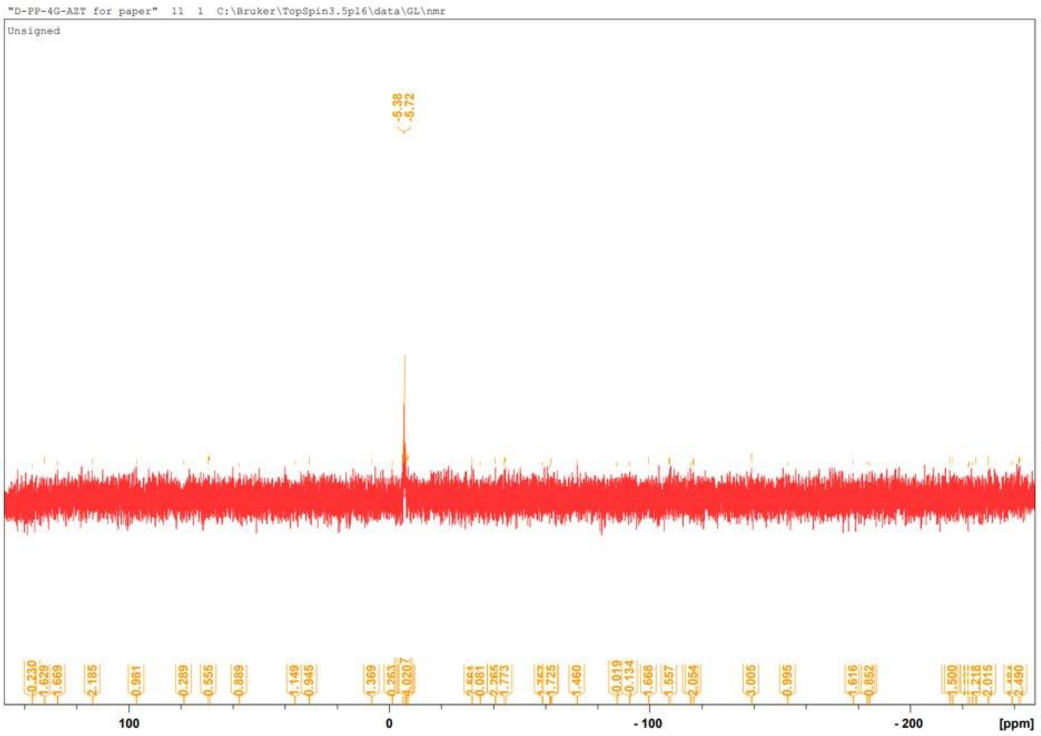

Figure S15. ^{31}P NMR trace (*N*Phe)$_4$GGGGk(AZT)y(p)-OH, peak at -5.72 demonstrates the presence and retention of the phosphate grouping on the tyrosine motif.

Electrospray ionization-mass spectrometry (ESI-MS)

ESI-MS (Waters LCT Premier, Waters, Hertfordshire, UK), with inbuilt Mass Lynx software to predict the theoretical mass was performed and analyzed by Analytical Services and Environmental Projects (ASEP) in the School of Chemistry, Queen's University Belfast. 10 mg of sample was dissolved in methanol prior to analysis.

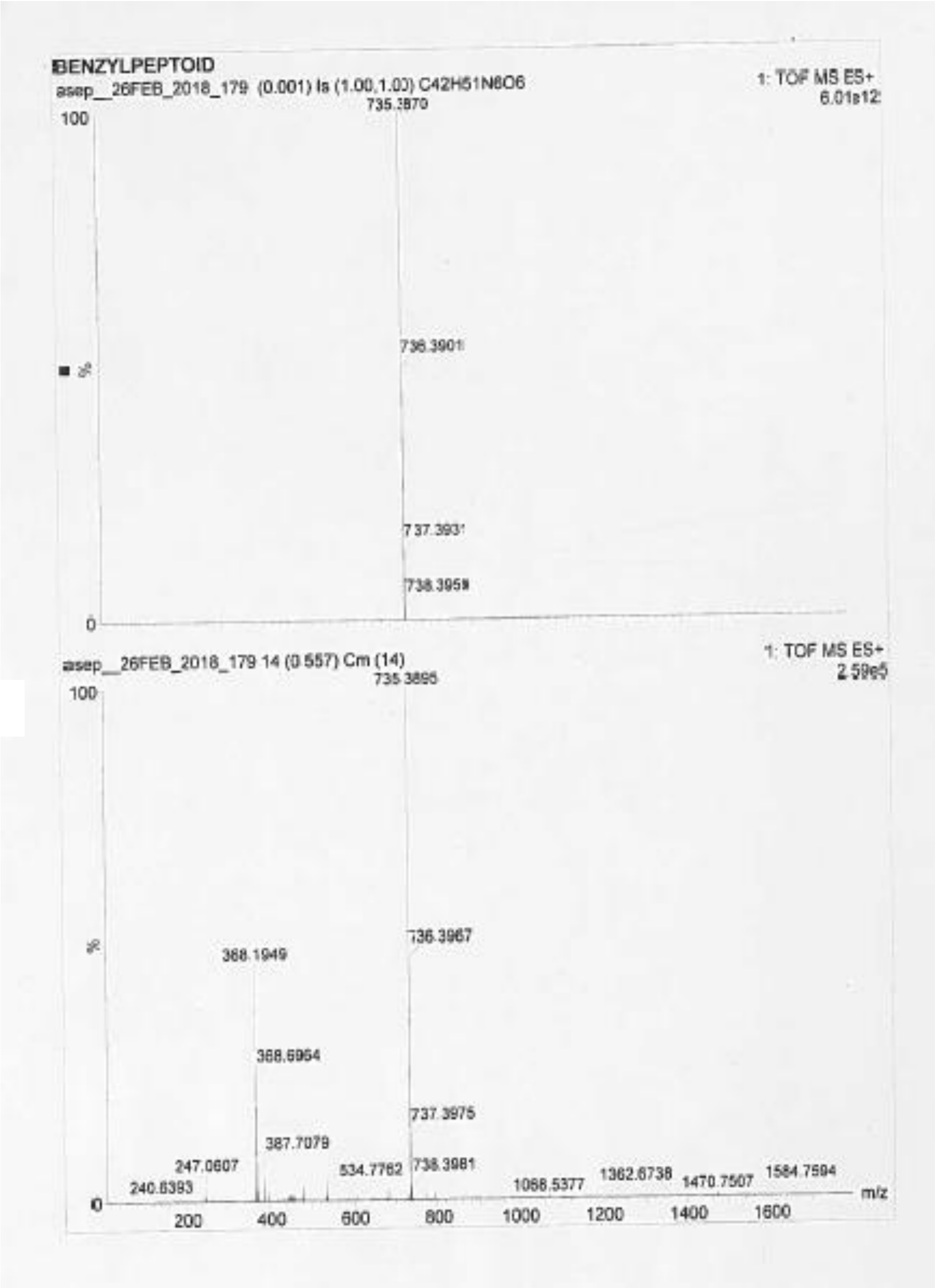

Figure S16. Expected (i) and observed (ii) ESI-MS traces for (*N*Phe)$_4$(*N*Lys)Y(p)-OH, identity confirmed via peak at 736 (M + H$^+$).

i

ii

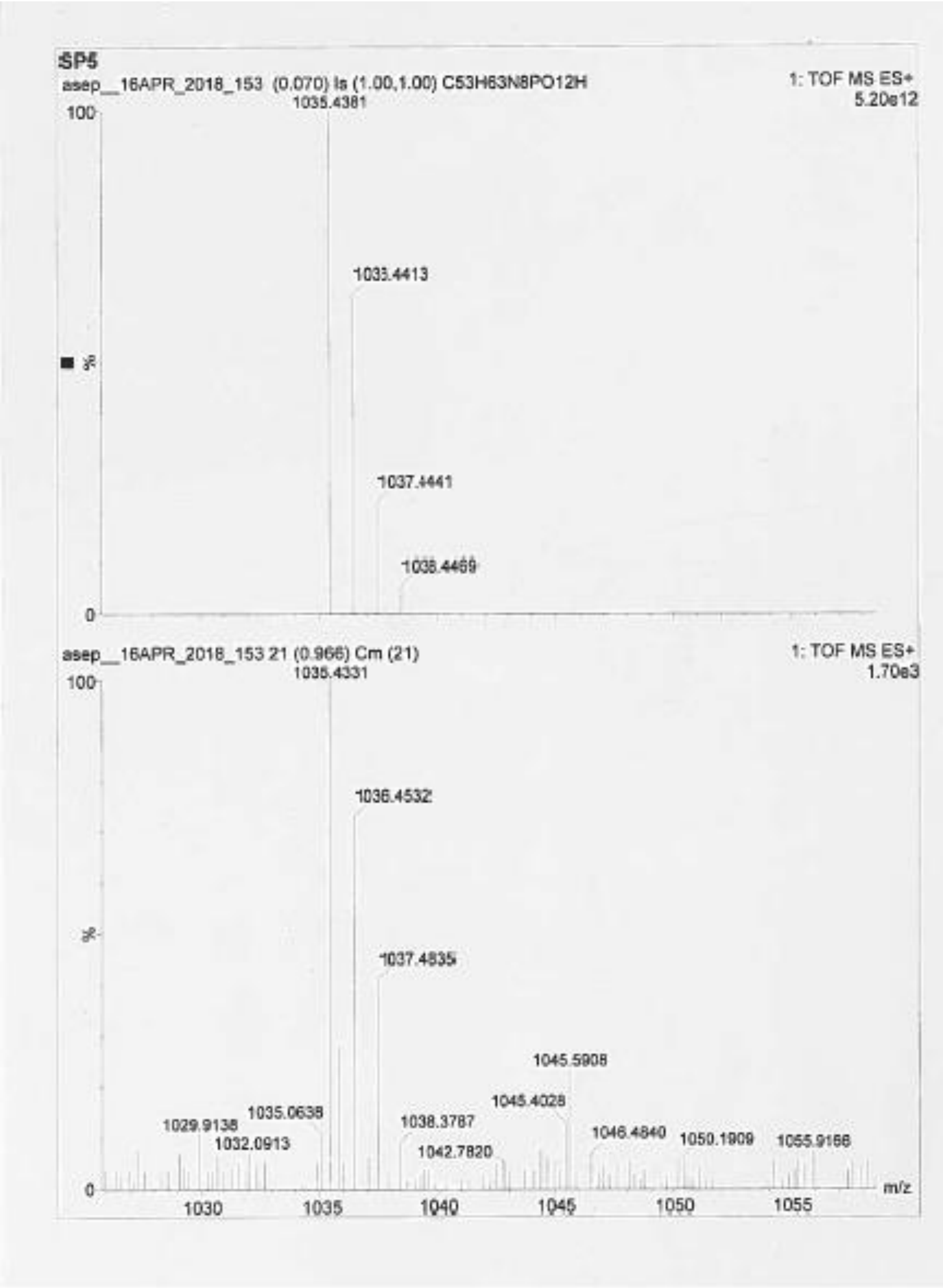

Figure S17. Expected (i) and observed (ii) ESI-MS traces for (*N*Phe)$_4$(*N*Lys)Y(p)G-OH, identity confirmed via peak at 1036 (M + H$^+$).

i

ii

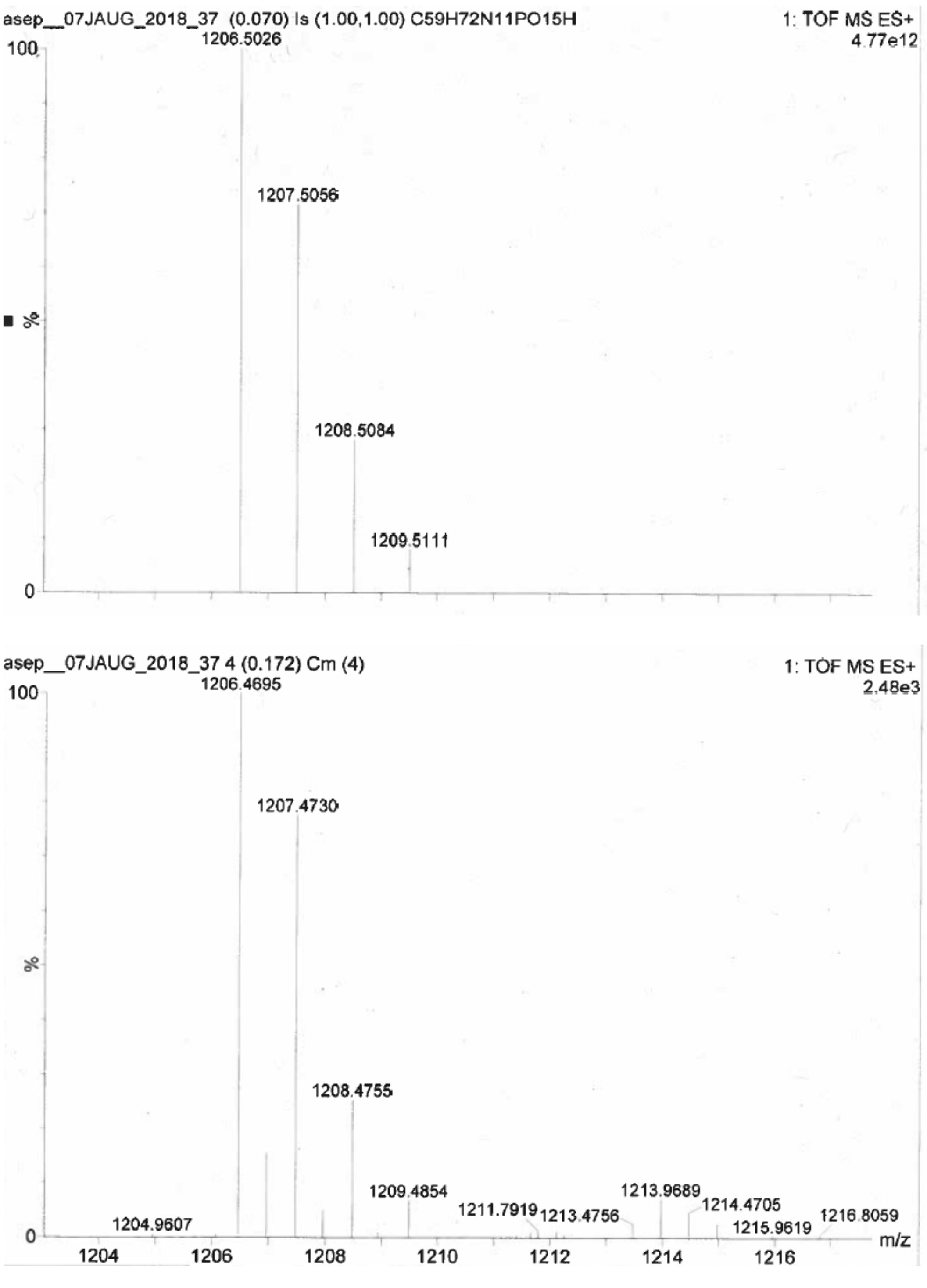

Figure S18. Expected (i) and observed (ii) ESI-MS trace for (*N*Phe)$_4$GGGGKY(p)-OH, identity confirmed via peak at 1207 (M + H$^+$).

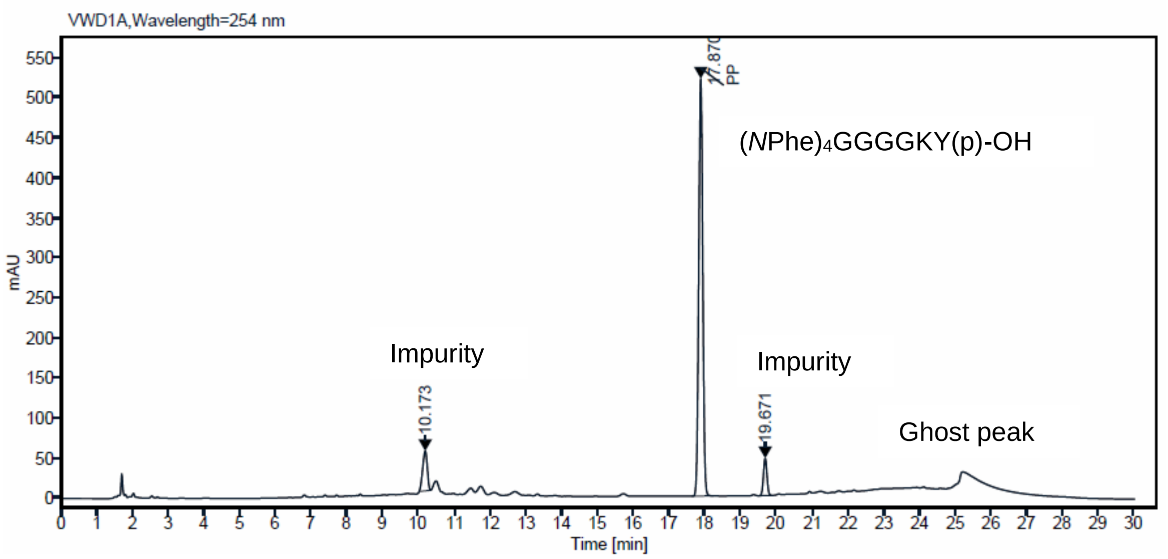

Figure S19. HPLC chromatogram for (*N*Phe)$_4$GGGGKY(p)-OH.

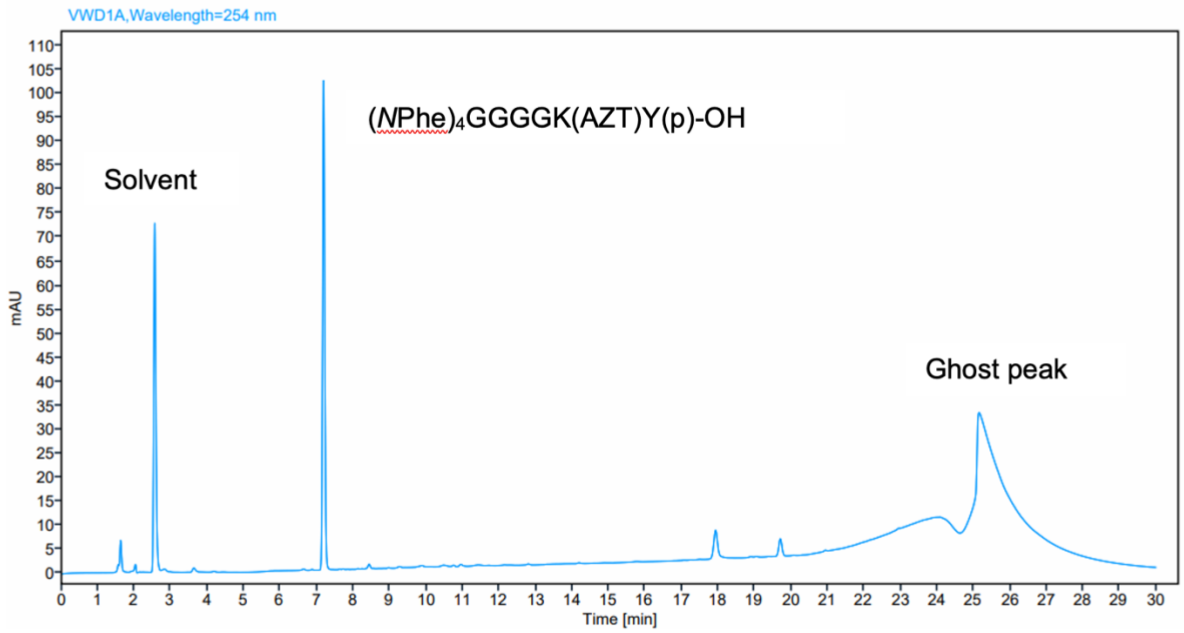

Figure S20. HPLC chromatogram for (*N*Phe)$_4$GGGGK(AZT)Y(p)-OH.

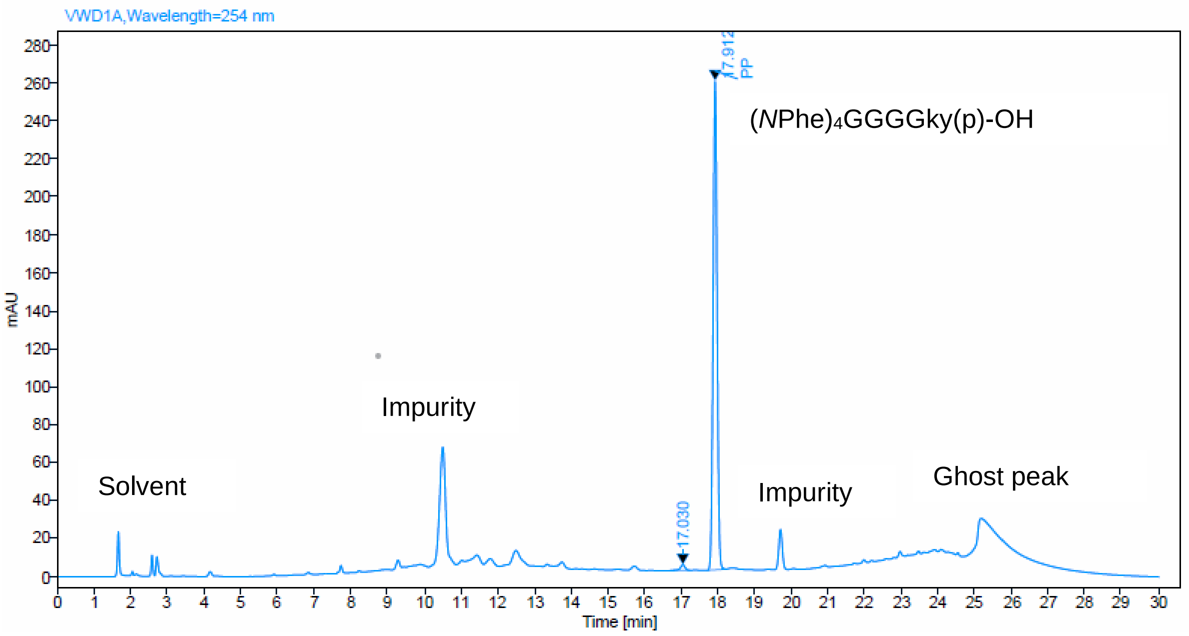

Figure S21. HPLC chromatogram for (*N*Phe)$_4$GGGGky(p)-OH.

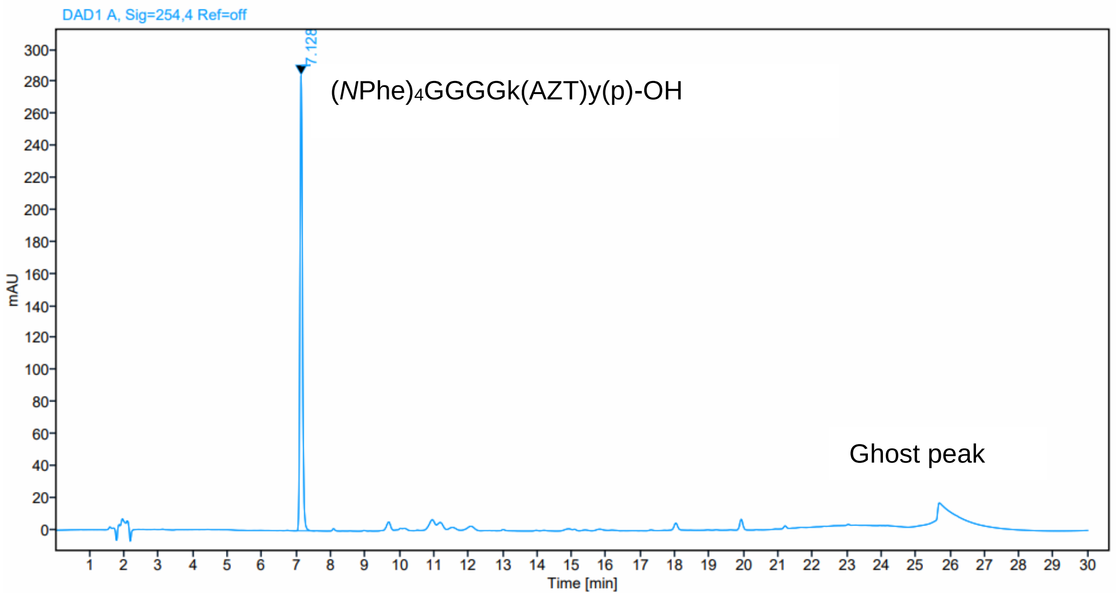

Figure S22. HPLC chromatogram for (*N*Phe)$_4$GGGGk(AZT)y(p)-OH.

S.3. Formulation of hydrogels and vial inversion assays

Vial inversion assay: The ability of peptoid-peptides to gelate in the presence of phosphatase enzyme was initially screened by adding 2 μL of a 1000 U mL^{-1} (total 2 U) alkaline phosphatase solution derived from bovine intestinal mucosa (Merck Life Science UK Ltd, Dorset, UK) to a known concentration of peptoid-peptide solution formulated in pH 7.4 phosphate-buffered saline PBS (Table S2). This mixture was incubated overnight at 37°C. In the case of no observed propensity to gelate, governed by a failure to self-suspend within the HPLC vial, fresh peptoid-peptide samples were heated to 70°C to ensure full dissolution and then cooled to room temperature.[6] Once again, 2 U of alkaline phosphatase was added and this was similarly observed for the propensity to gelate at 37°C. All peptoid-peptides that failed to gelate upon initial addition of phosphatase enzymes also failed to form gels when a heating/cooling step was introduced.

Microscopy: Hydrogels were formulated as outlined in Table S2 and 80 μL of each gel was pipetted onto an SEM sample mount at a concentration of 5% w/v. The samples were flash frozen in liquid nitrogen, lyophilized overnight and coated with an 8 nm layer of gold prior to imaging at 3 kV.[7] Transmission electron microscopy (TEM) was also performed using a JEOL JEM 1400 Plus TEM (JEOL, Freising, Germany). The hydrogels were once again formulated at 5% w/v (Table S2) and 3 μL of each hydrogel was pipetted onto a TEM copper grid. This mixture was left to air dry and then examined.

Table S2. Stepwise formulation of a self-assembling enzyme-triggered gelator using 2% w/v* peptoid-peptide as an example (final volume 500 µL).

Formulation Step	Constituent	Quantity added
1	Peptoid-peptide, or peptoid-peptide-drug	10 mg pre-weighed in HPLC vial
2	1.0 M NaOH	10 µL
3	PBS	200 µL[a)]
4	1.0 M NaOH	10 µL (according to pH, keep to 7.4)
5	PBS	200 µL[a)]
6	PBS	to final volume (500 µL)[a)]
7	Alkaline phosphatase	2 U (2 µL)[b)]

[a)] Sonicate (30 minutes) using a Branson 3510 sonic bath (Branson Ultrasonics Danbury, Connecticut, USA). Then the pH was monitored using a pH probe.

[b)] Overnight incubation at 37°C.

* For 5% w/v peptoid-peptide 25 mg of gelator is made up to a final volume of 500 µL.

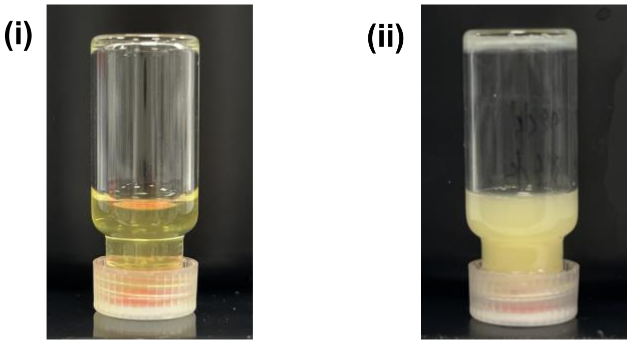

Figure S23. Vial inversion assay demonstrating non-gelation of 5% w/v (*N*Phe)$_4$(*N*Lys)Y(p)GGG-OH, (i) dissolved in PBS prior to addition of 2 U alkaline phosphatase, (ii) after 2 U alkaline phosphatase addition and formulation as outlined in Table S2.

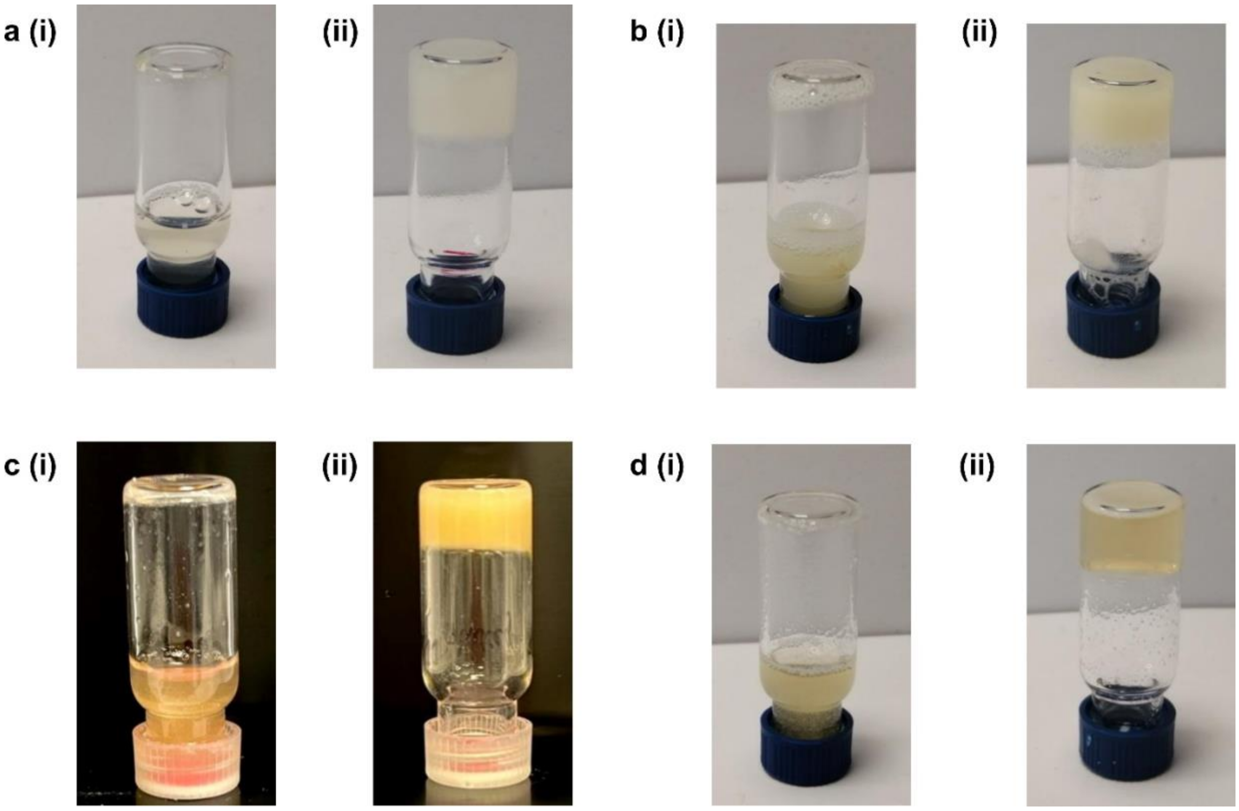

Figure S24. Vial inversion assay for a) 2% w/v (*N*Phe)$_4$GGGGKY(p)-OH, b) 5% w/v (*N*Phe)$_4$GGGGKY(p)-OH, c) 2% w/v (*N*Phe)$_4$GGGGk(AZT)y(p)-OH and d) 5% w/v (*N*Phe)$_4$GGGGK(AZT)Y(p)-OH. (i) peptoid-peptide sequence dissolved in PBS prior to addition of 2 U alkaline phosphatase, (ii) after 2 U alkaline phosphatase addition and formulation as outlined in Table S2.

S.4. Oscillatory rheology

Oscillatory rheology: When gelation propensity was demonstrated, the mechanical properties of each peptoid-peptide ± zidovudine were characterized via oscillatory rheology using an Anton Paar MCR302 rheometer (Anton Paar, St Albans, UK). Gels were formulated at a 2 mL volume in 7 mL Sterilin™ vials as previously described.[8] A vane and cup geometry was used to perform both frequency and strain sweeps. Frequency sweeps were performed at 37°C from 1 – 100 rad s^{-1} at a strain of 0.5%. Stress sweeps were performed at 37°C, 2.5 rad s^{-1} and a strain of 0.1 – 1000%. Gel strength was measured by determining the strain at which the gels

broke (breakage strain or flow point) when the loss modulus was greater than or equal to the storage modulus ($G'' \geq G'$). The time required for the peptoid-peptides to gelate in the presence of phosphatase enzymes was characterized by rheological time sweeps, performed using a sandblasted parallel plate (PP50/S) with a 0.5 mm measuring gap at 37°C for a period of up to 24 hours. Each rheological test was performed in triplicate for 5% w/v gels.

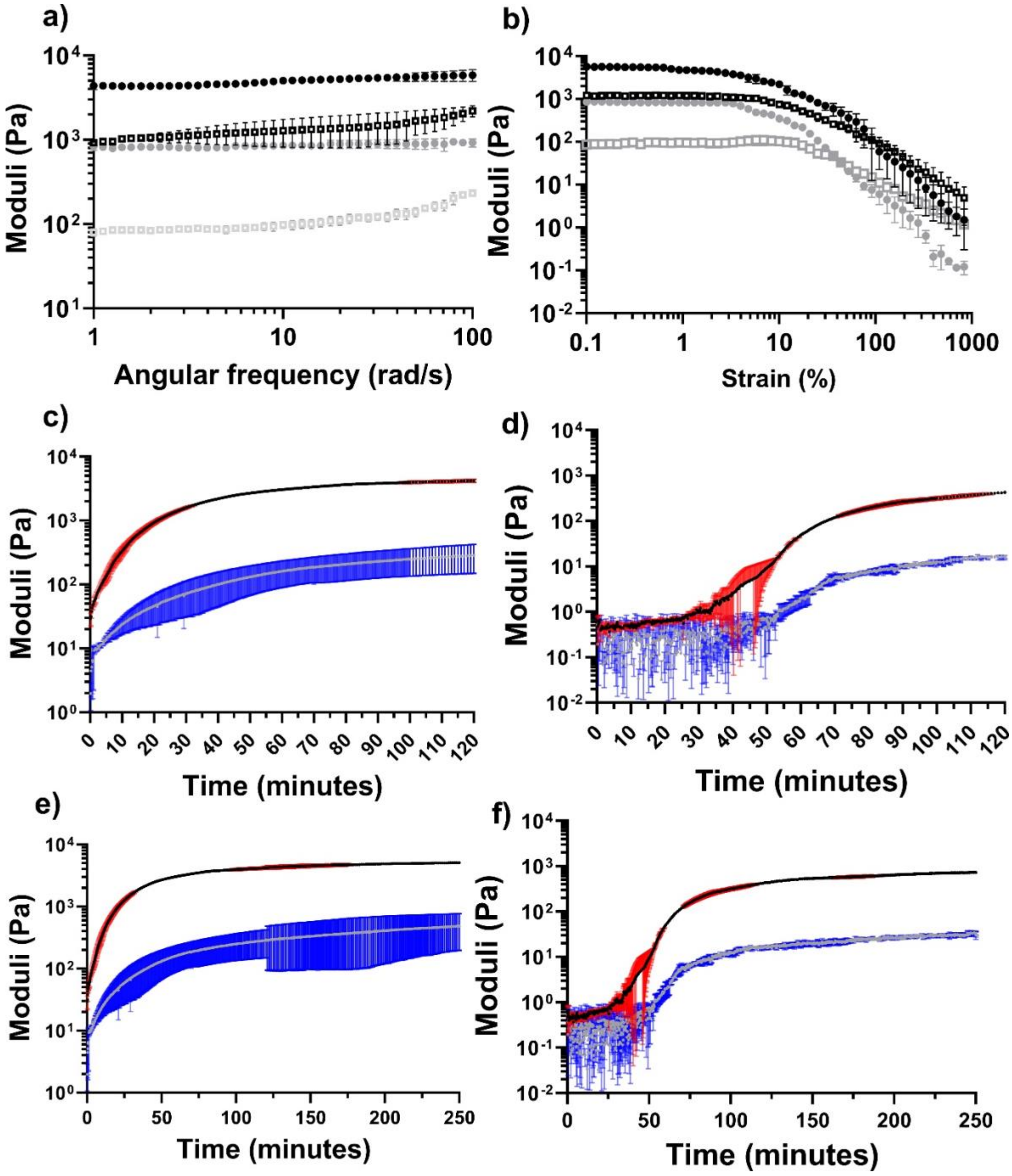

Figure S25. Rheological data relating to peptoid-L-peptides, 5% w/v (*N*Phe)$_4$GGGGKY(p)-OH and 5% w/v (*N*Phe)$_4$GGGGK(AZT)Y(p)-OH. Means ± standard deviations (SDs) plotted for each (n = 3). a) Frequency sweeps for 5% w/v peptoid-L-peptide hydrogels. b) Strain sweeps for 5% w/v peptoid-L-peptide gels. In a) and b), the peptoid-L-peptide (*N*Phe)$_4$GGGGKY-OH is presented in black, and the peptoid-D-peptide with zidovudine-attached (*N*Phe)$_4$GGGGK(AZT)Y-OH is presented in gray. The filled circles represent the

storage modulus (G′), and the open squares represent the loss modulus (G″). Rheological time sweeps to 120 min for c) 5% w/v (*N*Phe)$_4$GGGGKY(p)-OH and d) 5% w/v (*N*Phe)$_4$GGGGK(AZT)Y(p)-OH. In c) and d), the black lines represent the storage modulus (G′), and the gray lines represent the loss modulus (G″) with the red and blue areas donating SDs for G′ and G″ respectively. e) and f) demonstrate rheological time sweeps across 250 minutes for e) 5% w/v (*N*Phe)$_4$GGGGKY(p)-OH and f) 5% w/v (*N*Phe)$_4$GGGGK(AZT)Y(p)-OH.

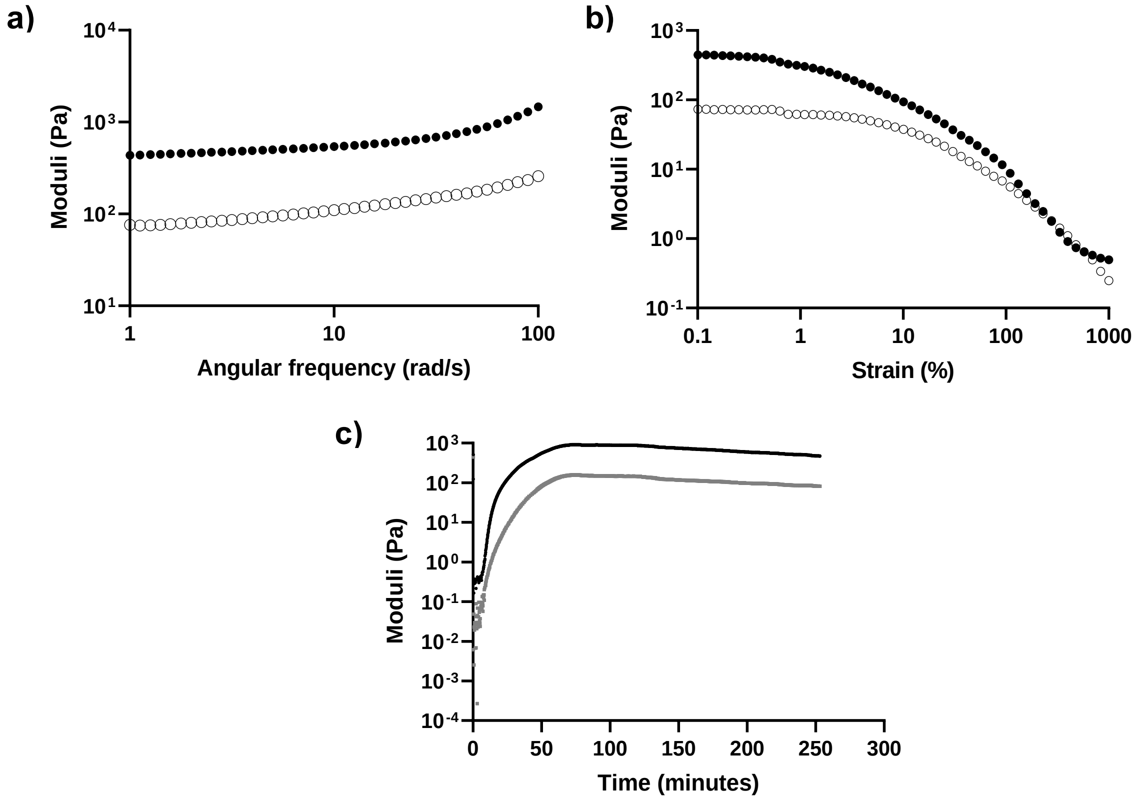

Figure S26. a) Frequency sweep, b) strain sweep and c) time sweep showing the rheological properties of 2% w/v (*N*Phe)$_4$GGGGky(p)-OH (n = 1). In a) and b) black filled circles represent storage modulus (G′), and black open circles represent loss modulus (G″). In c) black line represents G′ and gray line represents G″.

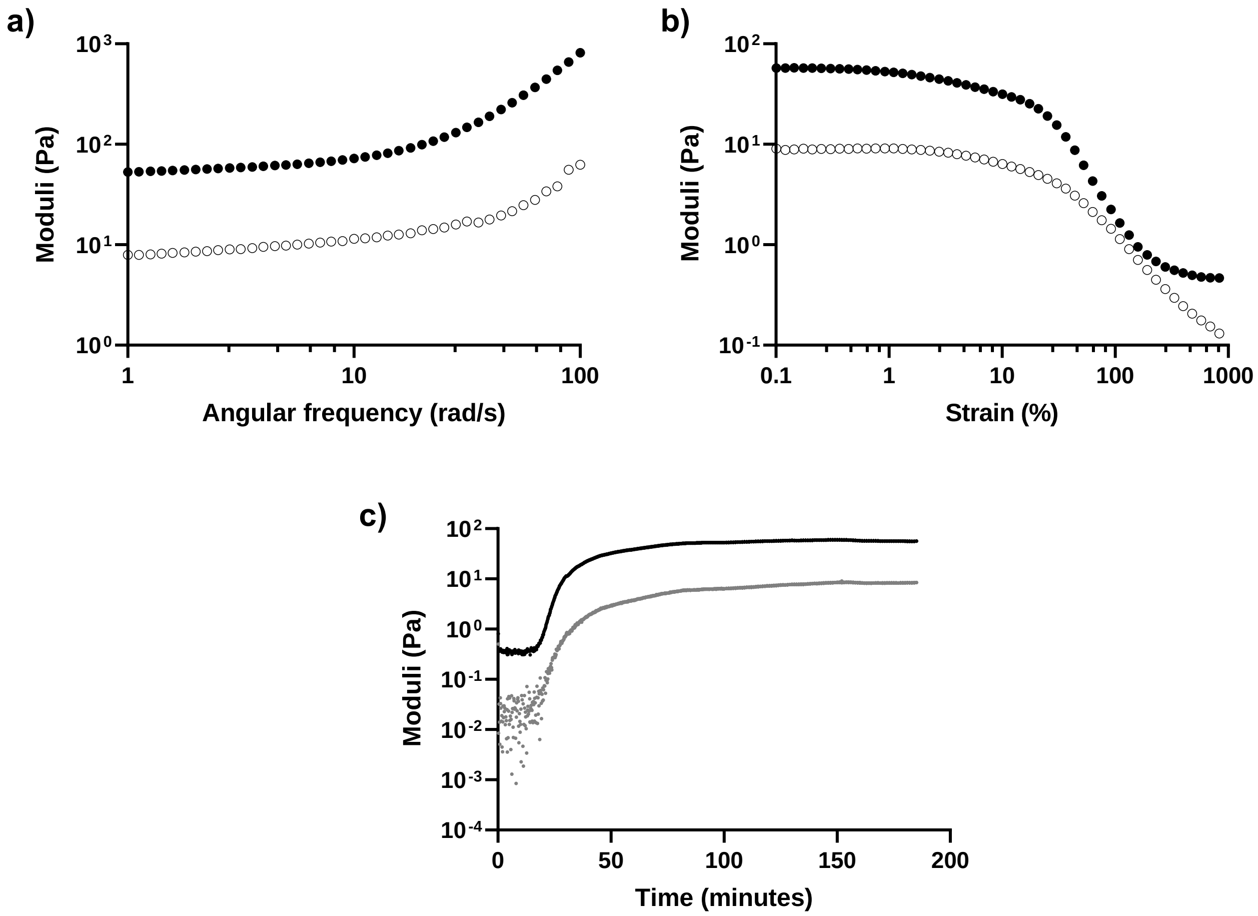

Figure S27. a) Frequency sweep, b) strain sweep and c) time sweep showing the rheological properties of 2% w/v (*N*Phe)$_4$GGGGk(AZT)y(p)-OH (n = 1). In a) and b) black filled circles represent storage modulus (G′), and black open circles represent loss modulus (G″). In c) black line represents G′ and gray line represents G″.

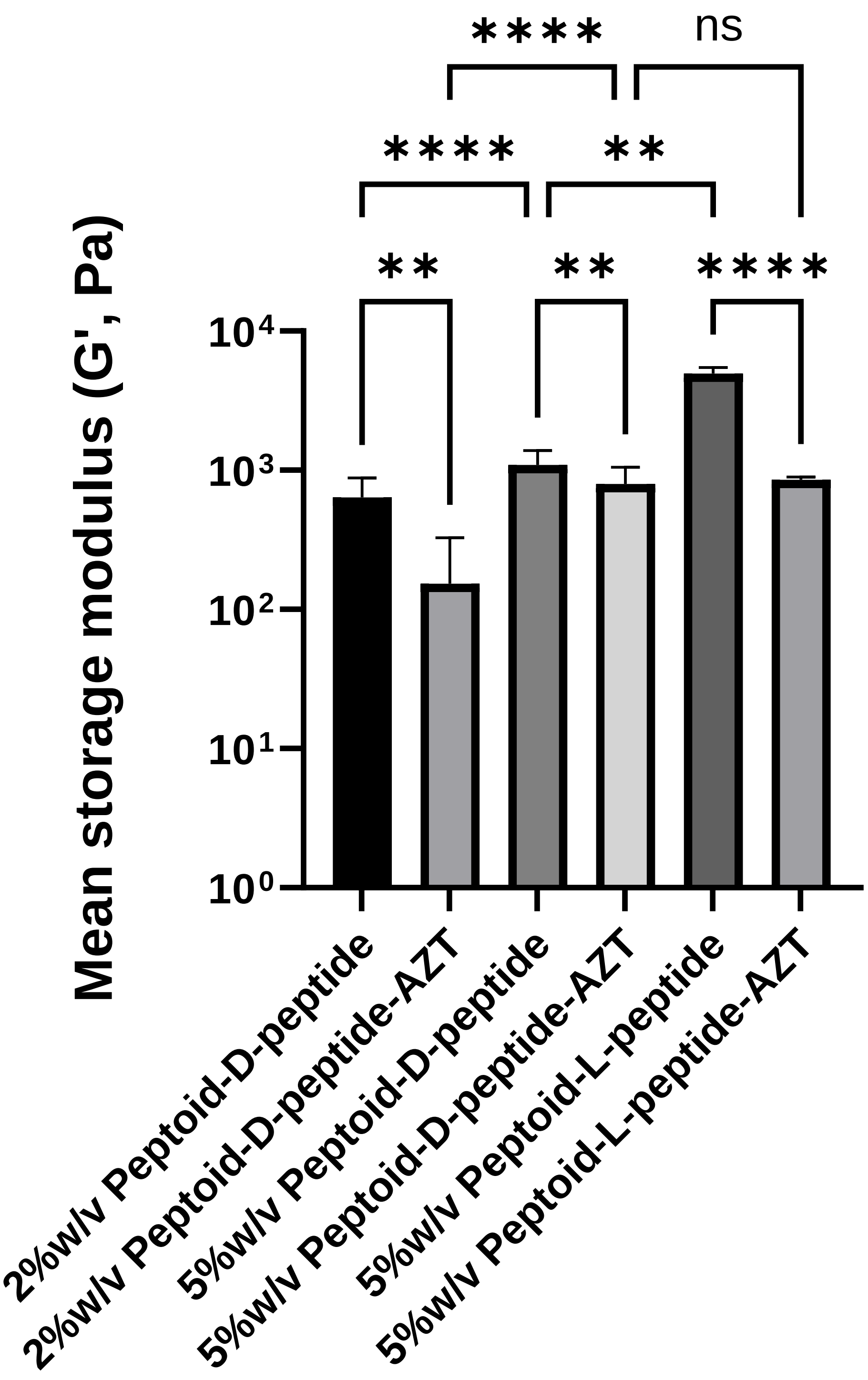

Figure S28. Mean value of storage modulus (G′) for each peptoid-peptide derived from individual frequency sweeps (1 – 100 rad s^{-1}, strain = 0.5%) providing a comparison of gel stiffness. ns: no significant ($p > 0.05$), *$p \leq 0.05$, ****$p \leq 0.0001$ difference in G′.

Table S3. Gelation times for peptoid-L-peptide and peptoid-D-peptide hydrogels upon addition of alkaline phosphatase enzyme derived from data in Figures S25 – S27 S40 and Figures 2e, f.

**Sequence**	**G′ and G″ cross**	**G′ > 2x G″**	**Time for G′ and G″ to stabilize**	*** Tan δ = G″/G′ from freq sweeps**
**Peptoid-L-peptides**				
5% w/v (*N*Phe)$_4$GGGGKY(p)-OH	Within 10 seconds	Within 10 seconds	80 min	1340.27/ 4964.56 = 0.270
5% w/v (*N*Phe)$_4$GGGGK(AZT)Y(p)-OH	Within 1 min	Within 1 min	135 min	112.18/856.11 = 0.131
**Peptoid-D-peptides**				
2% w/v (*N*Phe)$_4$GGGGky(p)-OH	Within 30 seconds	Within 1 min	70 min	124.76/635.92 = 0.196
5% w/v (*N*Phe)$_4$GGGGky(p)-OH	Within 1 min	Within 1 min	70 min	177.90/1090.68 = 0.163
2% w/v (*N*Phe)$_4$GGGGk(AZT)y(p)-OH	Within 30 seconds	Within 1 min	80 min	131.71/796.70 = 0.165

5% w/v (*N*Phe)$_4$GGGGk(AZT)y(p)-OH	Within 30 seconds	Within 30 seconds	90 min	138.84/835.57 = 0.166

* Mean value of storage modulus (G′) and loss modulus (G″) for each peptoid-peptide derived from individual frequency sweeps (1 – 100 rad s^{-1}, strain = 0.5%)

S.5. Small angle neutron scattering (SANS)

Hydrogels were formulated within a Hellma 2 mm path length UV spectrophotometer grade quartz cuvette (Hellma UK Ltd, Southend-on-Sea, UK) at 5% w/v as described in Table S2. Deuterated water (D_2O) was used as a solvent rather than PBS to enhance contrast.[9] Neutron scattering from gels was collected over a wide Q range ($Q = 4\pi \sin(\theta/2)/\lambda$) of 0.001 to 0.5 Å^{-1} and across three different sample-detector distances (1.4, 8, and 39 m) as previously described.[10] The scattering data obtained from D11 were reduced to 1D scattering curves (intensity vs Q) utilizing software (GRASP) provided by the ILL facility. Background data (electronic background, D_2O control, empty cuvette) were subtracted from the scattering data and the full detector images were normalized. SasView software version 5.0.6 was used to fit the data to several models including a cylinder, an elliptical cylinder, a flexible cylinder, a flexible elliptical cylinder and hollow cylinders, all with and without the power law applied. Cylinder-based models were applied given that long anisotropic structures were observed via for both SEM and TEM.[11] A summary of the fitting parameters is shown in Table S4. The data and the fits are shown in Figure 3c and d.

Table S4. Summary of the SANS fitting parameters for 5% w/v formulated hydrogels.

**Parameter**	**(*N*Phe)$_4$GGGGKY(p)-OH**	**(*N*Phe)$_4$GGGGK(AZT)Y(p)-OH**
Scale	0.00047142 +/- 5.1299e-6	0.00047142 +/- 5.1299e-6
Background (cm^{-1})	0.035416 +/- 2.9883e-5	0.091411 ± 4.4435e-05
Length (Å)	36559 +/- 9290	2.3175e+13 ± 1e+08
Khun length (Å)	221.98 +/- 2.3367	129.94 ± 1.0299
Radius (Å)	18.246 +/- 0.038767	7.8128 ± 0.14457
Axis ratio	8.7413 +/- 0.091765	2.4808 ± 0.061651
SLD (x10^{-6} Å^{-2})	2.461	2.461
SLD solvent (x10^{-6} Å^{-2})	6.39	6.39
X^2	15.367	7.7379

S.6. Biostability

Fully solubilized peptoid-peptides (1 mL, PBS, 37°C) were tested below the critical gelation concentration (0.02% w/v) in Eppendorf tubes to ensure that the gel strength and diffusion of proteinase K (3 U mL^{-1}) did not influence biostability.[12] Samples (100 µL) were removed at several time points (t = 0, 0.5, 1, 3, 6 and 24 hours; 3, 7, 14, 21 and 28 days), and the protease was inactivated via the addition of 50 µL of concentrated acetic acid. The samples were subsequently analyzed via RP-HPLC, and the percentage of (*N*Phe)$_4$GGGGKY(p)-OH or (*N*Phe)$_4$GGGGky(p)-OH remaining at each time point was calculated as outlined in Equation 1.

$$\text{\% Peptoid-peptide remaining} = \frac{[\text{Area of peptoid-peptide peak at test timepoint}]}{[\text{Area of peptoid-peptide peak at t = 0}]} \text{ x } 100$$

(Equation 1)

S.7. Cell cytotoxicity

The cells were initially cultured and grown at 37°C/5% CO_2 in minimum essential medium (MEM) supplemented with 10% fetal bovine serum and 1 mM sodium pyruvate (Invitrogen, Paisley, UK). NCTC 929 cells were subcultured at 80 – 90% confluence by removing the media and washing with sterile PBS, after which the cell monolayers were detached using 0.05% trypsin/0.53 mM EDTA.4Na solution (Invitrogen, Paisley, UK). Cells were cultured until at least the third passage and seeded at 1×10^4 cells per well (for 6 hours and 24 hours of treatment) and 5×10^3 cells per well (for 72 hours of treatment) in sterile Nunc™ 96-well microtiter plates (Sigma-Aldrich, Dorset, UK). After incubating the cells for 24 hours, the media was removed, and the cells were exposed to 100 µL of each solubilized peptoid-peptide with or without the attached drug at several different concentrations (20 – 500 µM). The peptoid-peptides was replaced with 100 µL of 70% v/v ethanol (100% kill, positive control) or media (100% viability, negative control) for the control samples.

The MTS-based CellTiter 96 AQueous One Solution Cell Proliferation Assay (Promega, Southampton, UK) was used to analyze the metabolic activity of treated cells.[3] UV absorption was measured at 490 nm using a Tecan Sunrise plate reader (Tecan UK Ltd, Reading, UK) and cell viability/percentage metabolic activity was calculated as outlined in Equation 2 below. This was reported as the mean of nine measurements (three replicates, repeated three times).

$$\%\ \text{Metabolic activity} = \frac{[(\text{Abs}_{490\text{nm}}\ \text{experimental well - Abs}_{490\text{nm}}\ \text{media})]}{[\text{Abs}_{490\text{nm}}\ \text{negative control - Abs}_{490\text{nm}}\ \text{media})]}\ \text{x}\ 100$$

(Equation 2)

LDH release was quantified as a measure of cytotoxicity using a Cytotoxicity Detection KitPLUS assay (Sigma-Aldrich, Dorset, UK) as previously described.[13] The cells were incubated, with each solubilized peptoid-peptide (20 – 500 µM) in combination with NCTC 929 cells for 6 hours, after which 100 µL of test reagent was added to each well, and incubated

for a further 5 min. A stop reagent (50 μL) was then added, and the UV absorbance was measured at 490 nm using a Tecan Sunrise plate reader. Several experimental controls were used for the LDH release assay, all of which demonstrated no significant increase or decrease in absorbance: i) a high LDH release control representing 100% toxicity was generated by adding 5 μL of lysis solution to non-treated cells 15 min before the addition of the test reagent; ii) each peptoid-peptide was solubilized in media at the highest tested concentration (500 μM); iii) the LDH standard was added to the media; and iv) an equal volume of the highest solubilized peptoid-peptide concentration (500 μM) and the LDH standard were added to the media. The percentage of cell cytotoxicity was calculated using Equation 3 below and is reported once again as the mean of nine measurements (three replicates, repeated three times).

$$\%\ \text{Cell cytotoxicity} = \frac{[(\text{Abs}_{490\text{nm}}\ \text{experimental well - Abs}_{490\text{nm}}\ \text{low LDH release})]}{[\text{Abs}_{490\text{nm}}\ \text{high LDH release - Abs}_{490\text{nm}}\ \text{low LDH release})]}\ \text{x}\ 100$$

(Equation 3)

Finally, a Live/Dead® Viability/Cytotoxicity Fluorescence Assay (Thermo Fisher Scientific, Waltham, MA, USA) was used to quantify the viability of the NCTC 929 cells via fluorescence microscopy (EVOS FL microscope, Thermo Fisher Scientific, Waltham, MA, USA) after 24 hours of incubation with peptoid-peptide±drug mixture (20 – 500 μM), as previously described.[3] Following incubation, the cells were treated for 20 min with a mixture of 2 μM calcein AM and 4 μM ethidium homodimer-1 in PBS. Single filter images (green fluorescent protein or Texas Red) were taken, the background was subtracted, and the threshold was adjusted to remove any remaining background pixels, leaving only cells. The numbers of live and dead cells were imaged and calculated using ImageJ 1.54 h software (National Institutes of Health, Maryland, USA). Each peptoid-peptide was tested three times at each concentration. The image was converted to a mask, and the watershed was applied to separate touching cells, which were counted using the automated ImageJ analyze particles plugin. For dead cells, the

size of the pixels included 50 infinity pixels, and for live cells, 120 infinity pixels with 0.00 – 1.00 circularity were employed.

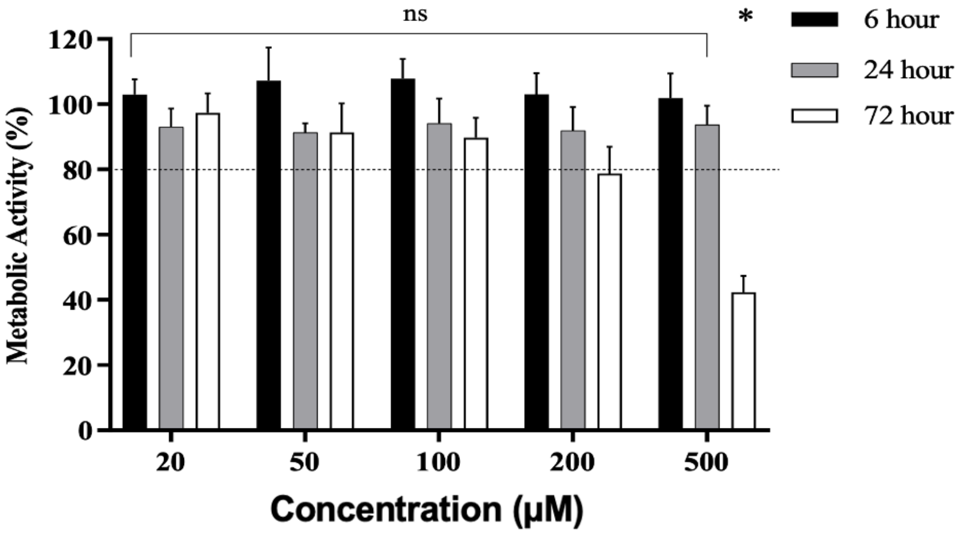

Figure S29. Cell metabolic activity of fully solubilized (*N*Phe)$_4$GGGGKY(p)-OH (μM range) using an MTS viability assay after 6, 24 and 72 hours. Means ± SD provided for nine replicates. ns: no significant ($p > 0.05$), $^*p \leq 0.05$ difference between the peptoid-L-peptide treatment and the negative control (media only).

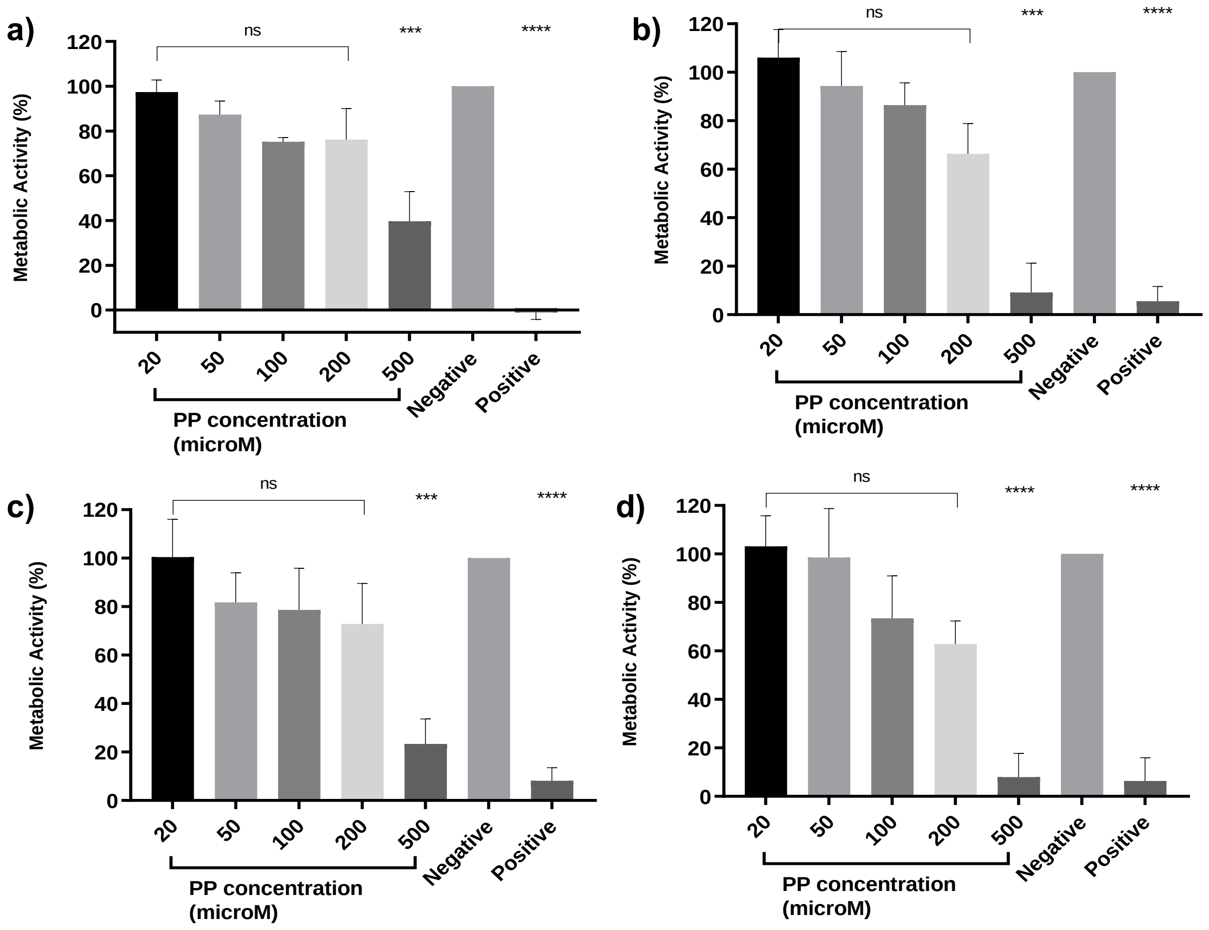

Figure S30. Cell metabolic activity of fully solubilized (*N*Phe)$_4$GGGGky(p)-OH (µM range) using an MTS viability assay after a) 6 h, b) 24 h, c) 48 h and d) 72 hours. Means ± SD provided for nine replicates. ns: no significant ($p > 0.05$), *$p \leq 0.05$, **$p \leq 0.01$, ***$p \leq 0.001$, ****$p \leq 0.0001$ difference between the peptoid-D-peptide (PP) treatment and the negative control (media only). 70% v/v ethanol acted as the positive control (100% kill).

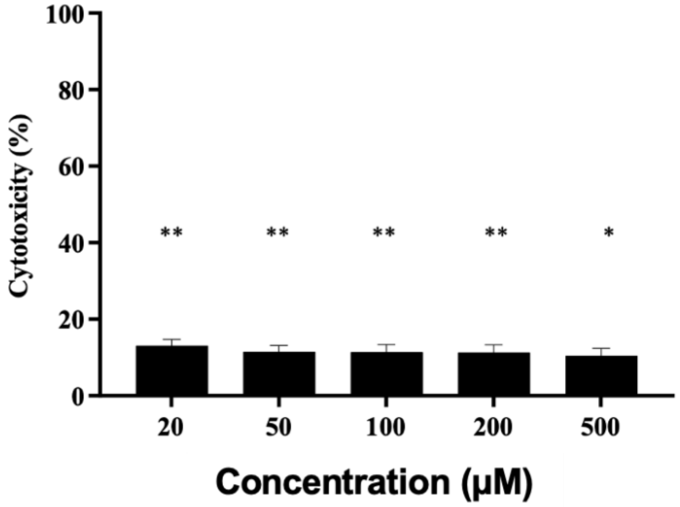

Figure S31. Cell cytotoxicity of fully solubilized (*N*Phe)4GGGGKY(p)-OH (µM range) using an LDH cytotoxicity assay after 6 hours. Means ± SD provided for nine replicates. NS: no significant ($p > 0.05$), *$p \leq 0.05$, **$p \leq 0.01$ difference between the peptoid-L-peptide and the negative control (media only).

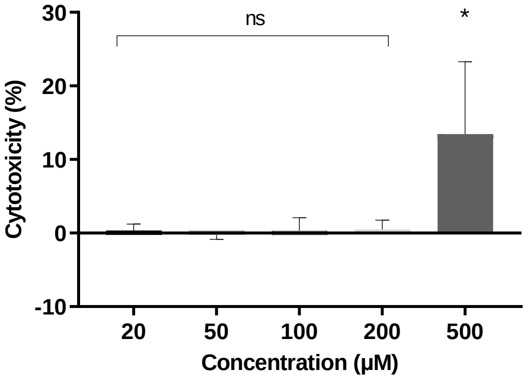

Figure S32. Cell cytotoxicity of fully solubilized (*N*Phe)4GGGGky(p)-OH (µM range) using an LDH cytotoxicity assay after 6 hours. Means ± SD provided for nine replicates. NS: no significant ($p > 0.05$), *$p \leq 0.05$, **$p \leq$ 0.01 difference between the peptoid-D-peptide and the negative control (media only).

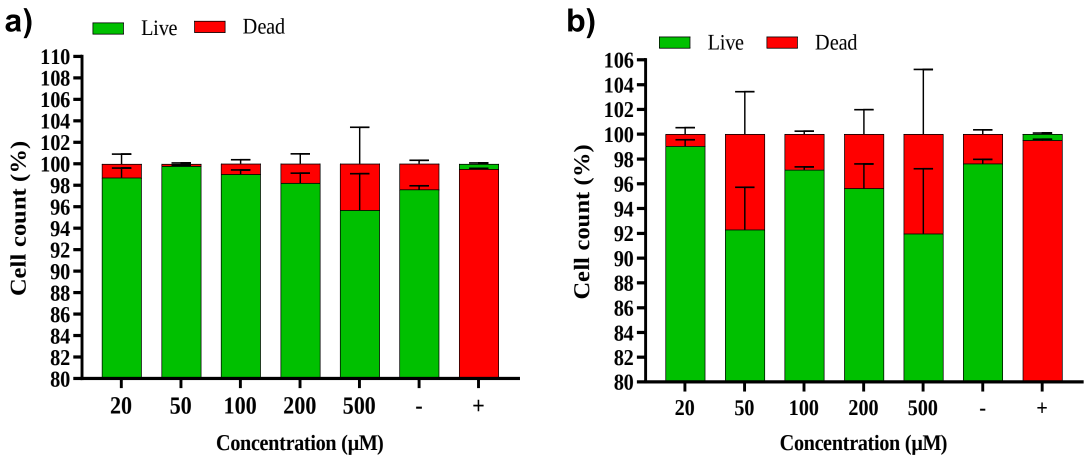

Figure S33. Live/Dead® cell counts for 24 hours treatment with peptoid-L-peptide conjugate in solubilized form using ImageJ analysis, where a) (*N*Phe)4GGGGKY(p)-OH and b) (*N*Phe)4GGGK(AZT)Y(p)-OH, +: positive control (70% ethanol), -: negative control (media only), green: % live cell count, red: % dead cell count. Data were collected from 10,000 cells per well using 96 well plates.

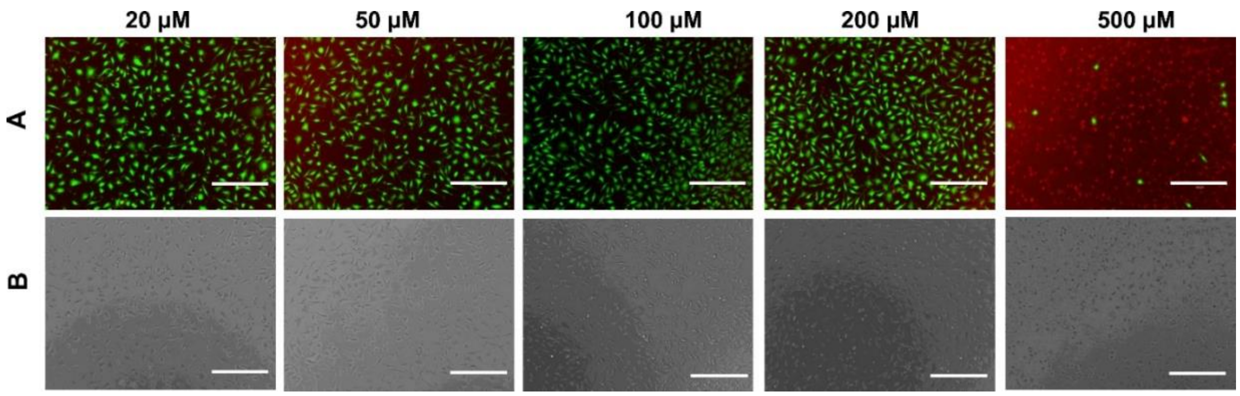

Figure S34. Cell cytotoxicity of peptides examined using Live/Dead® staining for a 24 hour treatment time with (*N*Phe)4GGGGky(p)-OH in solubilized form.

S.8. *In vitro* drug release

For the physically mixed formulations, lyophilized peptoid-peptide was mixed with zidovudine to a final concentration of 20% and 100 µL of the resulting mixture was used to form a 5% w/v hydrogel within glass vials after the addition of 2 U of alkaline phosphatase enzyme (Table S2). Peptoid-peptide hydrogels covalently attached to drugs were similarly formulated at a concentration of 5% w/v (Table S2). Once gels were formed, 1 mL of temperature-equilibrated (37°C) PBS was added to the top of each hydrogel. Glass vials were incubated in a thermostatic shaking water bath (37°C, 50 rpm). The supernatant (200 µL) was removed at each time point and replaced with 200 µL of fresh, temperature-equilibrated PBS in order to maintain sink conditions. Zidovudine release was determined by analytical HPLC, following British Pharmacopoeial methods (C_{18} gel column, UV spectrophotometry detection wavelength 270 nm).[14] The release properties of each peptoid-peptide was tested three times *in vitro*.

Table S5. Model fitting performed using KinetDS 3.0 rev. 2010 software with the r^2 value displayed for each model.

**Peptoid-peptide sequence**	**Model**					
	**Zero order**	**First order**	**Kormeyers-Peppas**	**Weibull**	**Hixson-Crowell**	**Higuchi**
	r^2	r^2	r^2	r^2	r^2	r^2
**(*N*Phe)$_4$GGGGKY-OH + AZT[a]**	0.1433	0.0328	0.8581	0.9327	0.0857	-2.3519
**(*N*Phe)$_4$GGGGK(AZT)Y-OH**	0.2544	0.0306	0.8410	0.8420	0.0823	-6.4988
(***N*Phe)$_4$GGGGky-OH + AZT[a]**	0.1420	0.0295	0.8438	0.8781	0.0663	-4.5942
**(*N*Phe)$_4$GGGGk(AZT)y-OH**	0.4666	0.0390	0.8660	0.8684	0.1828	-1.1992

[a] corresponds to physical encapsulation (Fig. 3f)

Table S6. The parameters fitted with the Kormeyers-Peppas model of drug release using KinetDS software for 28 day release profiles for each peptoid-peptide sequence.

**Peptoid-peptide sequence**	r^2	***k***	***n***
**(*N*Phe)$_4$GGGGKY-OH + AZT[a]**	0.8581	107.7±0.76	1.030±0.12
**(*N*Phe)$_4$GGGGK(AZT)Y-OH**	0.8410	17.35±0.75	0.9517±0.11
(***N*Phe)$_4$GGGGky-OH + AZT[a]**	0.8438	116.5±0.79	1.023±0.12
**(*N*Phe)$_4$GGGGk(AZT)y-OH**	0.8660	18.98±0.69	0.9701±0.11

[a] corresponds to physical encapsulation (Fig. 3f)

Table S7. The parameters fitted with the Weibull model of drug release using KinetDS software for 28 day release profiles for each peptoid-peptide sequence.

**Peptoid-peptide sequence**	**r^2**	***α***	***β***
**(*N*Phe)$_4$GGGGKY-OH + AZT[a]**	0.9327	0.1270±0.56	1.169±0.087
**(*N*Phe)$_4$GGGGK(AZT)Y-OH**	0.8420	5.362±0.75	0.9550±0.11
(***N*Phe)$_4$GGGGky-OH + AZT[a]**	0.8781	0.2762±0.73	1.088±0.11
**(*N*Phe)$_4$GGGGk(AZT)y-OH**	0.8684	4.836±0.68	0.9748±0.11

[a] corresponds to physical encapsulation (Fig. 3f)

S.9. *In vivo* drug release: An intravenous bolus of zidovudine was prepared aseptically in a laminar flow cabinet by dissolving the required mass of zidovudine in sterile water for injection BP. This treatment was then administered intravenously to the tail vein (8.46 mg kg^{-1}).[15] The intravenous zidovudine group was administered as a 100% bioavailability control, to validate our pharmaceutical analysis method and to prove the reliable detection and zidovudine extraction from blood plasma samples was possible. The peptoid-D-peptide experimental group received 300 µL of a 5% w/v (*N*Phe)$_4$GGGGk(AZT)y(p)-OH solution, containing 8.46 mg kg^{-1} of zidovudine, via subcutaneous injection. An (*N*Phe)$_4$GGGGk(AZT)y(p)-OH injectable solution was formed by dissolving lyophilized UV-sterilized (*N*Phe)$_4$GGGGk(AZT)y(p)-OH in sterile water for injection BP within a laminar flow cabinet, after which the pH was adjusted to 7.4 using 0.1 M sodium hydroxide. Blood samples (~0.2 mL) were obtained from the tail vein of each rat and collected in 1.5 mL preheparinized microtubes across 48 hour timepoints for the intravenously administered zidovudine group and across 35 day timepoints for the (*N*Phe)$_4$GGGGk(AZT)y(p)-OH subcutaneously administered

group. Centrifugation (13,400 rpm, 10 min) was utilized to separate blood plasma (supernatant), and the samples were stored at −80°C until the zidovudine concentration could be analyzed via analytical RP-HPLC and a validated calibration curve (Figure S35) as outlined below. Preliminary safety data were obtained by measuring the weight of each rat on day 0 and day 35. The pharmacokinetic parameters were calculated using PKSolver 2.0.[16, 17] The parameters studied were the drug half-life ($t_{1/2}$), the time to peak drug concentration (T_{max}), the maximum plasma concentration (C_{max}), the area under the plasma concentration – time curve from time zero to the last quantifiable concentration on day 35 ($AUC_{0–35}$), the observed area under the plasma concentration – time curve extrapolated from time zero to infinity ($AUC_{0-\infty,obs}$) and the mean residence time (MRT) of the drug extrapolated from time zero to infinity.

Sample size calculation: The La Mortes Power Calculation was employed as previously described, [3] with the following assumptions:

Control: 10 days with a standard deviation of 5 days

Treated: 30 days with a standard deviation of 10 days

La Mortes power calculation: [(Standard deviation A^2 - Standard deviation B^2) x $(Z1\text{-}alpha/2+Z1\text{-}beta)^2$]/$(Mean\ A - Mean\ B)^2$

The $(Z1\text{-}alpha/2+Z1\text{-}beta)^2$ was taken from standard tables, where alpha is the confidence level and beta is the statistical power. Therefore, for example based on 0.05 for both alpha and beta this value is 13. So: [(102 +52) x 13]/$(30\text{-}10)^2$ = 4.06 mice per group to attain 95% confidence levels. We have opted for 98% alpha confidence intervals where 6 mice are needed in each treatment group.

HPLC method: developed to determine the concentration of zidovudine in plasma using an Agilent 1260 Series system (UV detector, quaternary pump, auto sampling injector and Agilent

Chemstation) as previously described fitted with an ODS-3 analytical column (250 mm × 4.6 mm internal diameter, 5 µm packing; InertClone™, Phenomenex, USA).[3] The UV detector was operated at 266 nm at room temperature and a flow rate of 1.25 mL min^{-1}. The mobile phase was acetonitrile – 0.1% ortho-phosphoric acid in water (15 – 85 % v/v) was employed as the eluent.[15]

Preparation of zidovudine standards and zidovudine extraction from plasma: As described previously,[3] zidovudine stock solution (1 mg mL^{-1}) was prepared in Milli-Q water. This stock solution was diluted in Milli-Q water to the final concentrations required for the standard calibration curve, in the range 500 to 50000 ng mL^{-1}. 10 µL aliquots of each standard were added to 90 µL of rat plasma in 200 µL microtubes. The mixture was vortexed at 1400 rpm for 30 min at 25°C using an Eppendorf Theromomixer comfort and a final calibration standard concentration range of 50 to 5000 ng mL^{-1} was attained (Figure S35). Then, 50 µL of perchloric acid was added to each microtube to precipitate the plasma proteins. The mixture was vortexed at 1400 rpm for 20 min at 25°C. Finally, the samples were centrifuged at 13,400 rpm for 20 min at room temperature (Eppendorf minispin). The supernatants were collected into Supelco HPLC glass inserts inside HPLC vials. Nine calibrations were constructed over 3 days to validate the analytical method and ensure its reproducibility. Rat plasma samples were thawed to room temperature and 100 µL was transferred to microtubes. 50 µL of Perchloric acid was added to each microtube and vortexed at 1400 rpm for 30 min at 25°C to precipitate the plasma proteins. The samples were centrifuged at 13400 rpm for 20 min, and the supernatant collected in HPLC glass inserts inside HPLC vials. The pharmacokinetic parameters, area under the plasma concentration-time curve from time zero to the time of last measurable concentration (AUC_{o-t}), area under the plasma concentration – time curve from

time zero to infinity ($AUC_{0-\infty}$), plasma drug half-life ($t_{1/2}$) and mean residence time (MRT) were computed by a non-compartmental model applying the program PKSolver 2.0. [16]

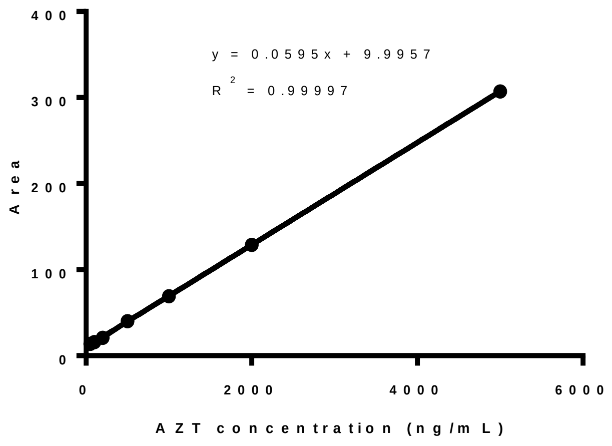

Figure S35. Calibration curve developed for zidovudine extracted from Sprague Dawley rat plasma (n = 9).

Table S8. Comparison of the *in vivo* pharmacokinetic parameters of zidovudine (AZT) after intravenous (IV) administration and subcutaneous (SC) administration of chemically conjugated (*N*Phe)$_4$GGGGk(AZT)y(p)-OH in Sprague Dawley rats. Means ± SEMs, displayed for each group (n = 6). The following parameters were calculated using PKSolver 2.0.[16] zidovudine drug half-life ($t_{1/2}$), time to peak drug concentration (T_{max}), the maximum plasma concentration (C_{max}), the area under the plasma concentration–time curve from time zero to the last sample at day 35 ($AUC_{0–35}$), the area under the plasma concentration vs time curve extrapolated from time zero to infinity ($AUC_{0-\infty,obs}$) and the mean residence time ($MRT_{0-\infty,obs}$) of the drug extrapolated from time zero to infinity.

**Parameter**	**Unit**	**Zidovudine (IV)**	**(*N*Phe)$_4$GGGGk(AZT)y(p)-OH (SC)**
$t_{1/2}$	hour	1.73 ± 0.53	2194.67 ± 1226.3
T_{max}	hour	0.58 ± 0.08	188.33 ± 104.38
C_{max}	µg mL^{-1}	6.48 ± 1.67	0.38 ± 0.19
AUC_{0-35}	µg mL^{-1} hour^{-1}	15.48 ± 2.60	53.30 ± 18.73
$AUC_{0-\infty,obs}$	µg mL^{-1} hour^{-1}	16.55 ± 2.73	341.25 ± 181.53
$MRT_{0-\infty,obs}$	hour	1.58 ± 0.50	3483.2 ± 1435.1

S.10. References

(1) Chan, W.; White, P. *Fmoc Solid Phase Peptide Synthesis: A Practical Approach*; Oxford University Press, 1999.

(2) Sieber, P. An improved method for anchoring of 9-fluorenylmethoxycarbonyl-amino acids to 4-alkoxybenzyl alcohol resins. *Tetrahedron Letters* **1987**, *28* (49), 6147-6150. DOI: https://doi.org/10.1016/S0040-4039(00)61832-4.

(3) Coulter, S. M.; Pentlavalli, S.; Vora, L. K.; An, Y.; Cross, E. R.; Peng, K.; McAulay, K.; Schweins, R.; Donnelly, R. F.; McCarthy, H. O. Enzyme-triggered L-α/D-peptide hydrogels as a long‐acting injectable platform for systemic delivery of HIV/AIDS drugs. *Advanced Healthcare Materials* **2023**, *12* (18), 2203198.

(4) Zuckermann, R. N.; Kerr, J. M.; Kent, S. B. H.; Moos, W. H. Efficient method for the preparation of peptoids [oligo (*N*-substituted glycines)] by submonomer solid-phase synthesis. *Journal of the American Chemical Society* **1992**, *114* (26), 10646-10647.

(5) Li, J.; Li, X.; Kuang, Y.; Gao, Y.; Du, X.; Shi, J.; Xu, B. Self-delivery multifunctional anti-HIV hydrogels for sustained release. *Advanced Healthcare Materials* **2013**, *2* (12), 1586-1590.

(6) Debnath, S.; Roy, S.; Abul-Haija, Y. M.; Frederix, P. W. J. M.; Ramalhete, S. M.; Hirst, A. R.; Javid, N.; Hunt, N. T.; Kelly, S. M.; Angulo, J.; et al. tunable supramolecular gel properties by varying thermal history. *Chemistry – A European Journal* **2019**, *25* (33), 7881-7887. DOI: https://doi.org/10.1002/chem.201806281.

(7) Porter, S. L.; Coulter, S. M.; Pentlavalli, S.; Thompson, T. P.; Laverty, G. Self-assembling diphenylalanine peptide nanotubes selectively eradicate bacterial biofilm infection. *Acta Biomaterialia* **2018**, *77*, 96-105. DOI: 10.1016/j.actbio.2018.07.033.

(8) McCloskey, A. P.; Draper, E. R.; Gilmore, B. F.; Laverty, G. Ultrashort self-assembling Fmoc-peptide gelators for anti-infective biomaterial applications. *Journal of Peptide Science* **2017**, *23* (2), 131-140. DOI: 10.1002/psc.2951.

(9) Draper, E. R.; Dietrich, B.; McAulay, K.; Brasnett, C.; Abdizadeh, H.; Patmanidis, I.; Marrink, S. J.; Su, H.; Cui, H.; Schweins, R. Using small-angle scattering and contrast matching to understand molecular packing in low molecular weight gels. *Matter* **2020**, *2* (3), 764-778.

(10) Cross, E. R.; Coulter, S. M.; Fuentes-Caparrós, A. M.; McAulay, K.; Schweins, R.; Laverty, G.; Adams, D. J. Tuning the antimicrobial activity of low molecular weight hydrogels using dopamine autoxidation. *Chemical Communications* **2020**, *56* (58), 8135-8138.

(11) McDowall, D.; Adams, D. J.; Seddon, A. M. Using small angle scattering to understand low molecular weight gels. *Soft Matter* **2022**, *18* (8), 1577-1590. DOI: 10.1039/D1SM01707A.

(12) Feng, Z.; Xu, B. Inspiration from the mirror: D-amino acid containing peptides in biomedical approaches. *Biomolecular concepts* **2016**, *7* (3), 179-187.

(13) McCloskey, A. P.; Gilmore, S. M.; Zhou, J.; Draper, E. R.; Porter, S.; Gilmore, B. F.; Xu, B.; Laverty, G. Self-assembling ultrashort NSAID-peptide nanosponges: Multifunctional antimicrobial and anti-inflammatory materials. *RSC Advances* **2016**, *6* (115), 114738-114749.

(14) British Pharmacopoeial Commission. *British Pharmacopoeia Monograph: Zidovudine.* The Stationery Office, 2024. https://www-pharmacopoeia-com.queens.ezp1.qub.ac.uk/bp-2024/monographs/zidovudine.html?date=2024-07-01&text=zidovudine (accessed 13th December 2023).

(15) Wannachaiyasit, S.; Chanvorachote, P.; Nimmannit, U. A novel anti-HIV dextrin–zidovudine conjugate improving the pharmacokinetics of zidovudine in rats. *AAPS PharmSciTech* **2008**, *9*, 840-850.

(16) Zhang, Y.; Huo, M.; Zhou, J.; Xie, S. PKSolver: An add-in program for pharmacokinetic and pharmacodynamic data analysis in Microsoft Excel. *Comput Methods Programs Biomed* **2010**, *99* (3), 306-314. DOI: 10.1016/j.cmpb.2010.01.007.
